# Synthesis and Electrochemistry of Formazan(ate) Re(I) Complexes: Ligand-Based Reactivity toward CO_2_

**DOI:** 10.1021/acs.inorgchem.5c03626

**Published:** 2025-10-17

**Authors:** Liliana Capulín Flores, Sander J. Mondria, Kai-Thorben Kuessner, Philipp Rohatschek, Inke Siewert, Noé Zúñiga-Villarreal, Edwin Otten

**Affiliations:** † Stratingh Institute for Chemistry, 3647 University of Groningen, Nijenborgh 3, 9747 AG Groningen, The Netherlands; ‡ Instituto de Química, Universidad Nacional Autónoma de México, Ciudad Universitaria, Circuito Exterior, 04510 México, D.F., Mexico; § Institut für Anorganische Chemie, 9375 Georg-August-Universität Göttingen, Tammannstr. 4, 37077 Göttingen, Germany

## Abstract

Re­(I) tricarbonyl complexes of the type *fac*-[ReX­(CO)_3_(L)]^*n*^ (*n* = −1, 0, +1) (X = Br^–^, MeCN), furnished with the redox-active formazan (L = Ph–N­(R)–N=CH–N=N–Ph; R = H, (**H5** ^***Br***^); R = Me, (**Me5** ^***X***^)) or formazanate (L = [Ph–N=N–C­(−Ph-4-R^1^)=N–N–Ph]^−^; R^1^ = H **[1** ^***Br***^ **]** ^**–**^, Me **[2** ^***Br***^ **]** ^**–**^, MeO **[3** ^***Br***^ **]** ^**–**^, F **[4** ^***X***^ **]** ^**–**^; L = [Ph–N=N–C­(−H)=N–N–Ph]^−^, Py = pyridine **5** ^***Py***^) ligands were prepared and characterized by spectroscopy and electrochemistry. *In situ* characterization of the reduced species by (spectroelectro)­chemical and computational methods revealed that the redox-active scaffold behaves as a two-electron sink, allowing two consecutive one-electron reductions to take place at the ligand. The reactivity of the reduced formazan­(ate) rhenium complexes toward CO_2_ was explored. (Spectroelectro)­chemical experiments along with DFT calculations suggested that CO_2_ reacts with the reduced formazanate Re­(I) complexes at low overpotentials forming a carbamate-type adduct. This ligand-based reactivity provides a thermodynamic sink for CO_2_ binding and hinders catalytic turnover via metal-centered CO_2_ activation. These findings provide new insights into the advantages and limitations of using catalysts with redox-active ligands to activate and convert small molecules.

## Graphical abstract

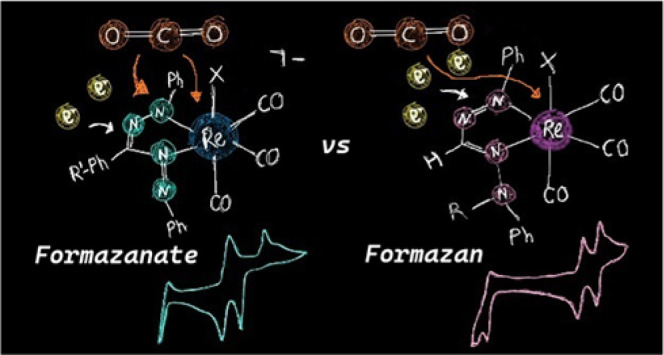

## Introduction

The use of fossil resources as the primary source of energy and transportation fuels in the past century has led to rapid increase in atmospheric CO_2_ levels and is linked to severe environmental impact due to global warming and climate change. Much effort is currently directed toward decarbonization, which requires large-scale electrification of industry, transport, and other sectors to achieve carbon neutrality. In this context, the catalytic conversion of CO_2_ via electrocatalytic reduction chemistry is a promising strategy to turn “waste” CO_2_ into valuable products for the chemical industry by using renewables (e.g., wind, solar power) as the energy input. The inert nature of CO_2_ necessitates the use of catalysts for substrate activation and to control the selectivity of reduction products that are formed. In particular, the initial transfer of an electron to CO_2_ is energetically costly, but the stabilization of the CO_2_^•–^ radical anion in the coordination sphere of a transition metal ion has been shown to facilitate electrocatalytic CO_2_ conversion to value-added chemicals such as the 2e^–^/2H^+^ reduction products CO (and H_2_O) and HCOOH,^1,2^ or more highly reduced C_1_ compounds,^3^ as well as C–C coupling products.^4–6^ The ability to control the reactivity of molecular (homogeneous) complexes by tailor-made ligands can be leveraged to develop catalysts that operate at lower overpotentials and have improved selectivity,^7,8^ making ligand design a cornerstone of catalyst development in this field.

After the discovery by Lehn and co-workers that (bipy)­Re­(CO)_3_Cl is an active catalyst for CO_2_ electroreduction to CO,^9,10^ there has been significant interest in complexes comprising the [Re­(CO)_3_]^+^ fragment with bidentate *N*-donor ligands. A key feature of such systems is their selectivity for CO_2_ reduction, which is attributed to the (partial) delocalization of the additional negative charge across the organic ligand^11^ thereby avoiding protonation at the metal center to form metal-hydride intermediates that lead to competing hydrogen evolution.^12^ The multielectron reduction of CO_2_ requires “pooling” of two (or more) redox-equivalents at the catalyst for productive turnover to occur, which can be facilitated by redox-active ligands^13^ that avoid the intermediacy of unstable metal oxidation states and allow catalysis at lower overpotential.^14–16^ In addition, ligands that are functionalized with peripheral acidic groups are known to improve catalytic performance by acting as proton-relay sites.^17–20^ Thus, synergetic effects between metal and (“non-innocent”) ligands have gained a prominent role in the development of new catalysts for CO_2_ reduction and related chemistries, using strategies that are often inspired by the mechanisms of enzymes in biological systems.^21^ Given the inherent complexity of multielectron/multiproton reaction pathways, the role of ligand-centered reactions in CO_2_ electrocatalysis is often still poorly understood. Various examples in the literature have demonstrated the ability of redox non-innocent fragments to capture electrons and drive the activation step, shifting the *locus* of reactivity to the organic scaffold (Chart 1a).^12,22–27^

**Chart 1:**
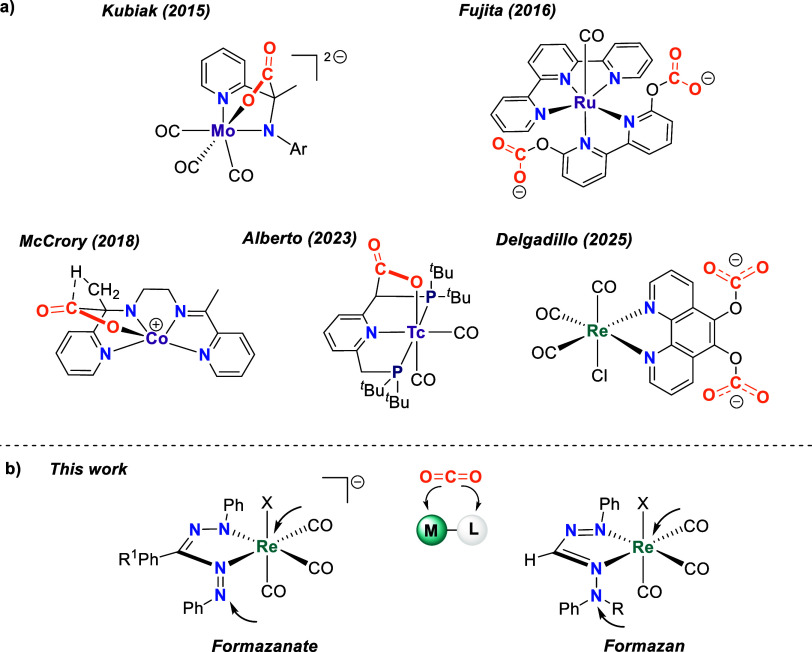
(a) Examples of Substrate Activation with Redox Non-innocent Ligands; (b) Complexes Explored in This Work, R^1^ = H (**[1** ^***Br***^ **]** ^**–**^), Me (**[2** ^***Br***^ **]** ^**–**^), MeO (**[3** ^***Br***^ **]** ^**–**^), F (**[4** ^***X***^ **]** ^**–**^), R = H (**H5** ^***Br***^), Me (**Me5** ^***X***^) and X = Br^–^, MeCN[Fn c1fn1]

a The arrows represent the potential reactive sites within these complexes that can lead the activation of CO_2_.

Formazanates are a class of redox-active anionic ligands comprising the core [−N=N–C­(−R′)=N–N−]^−^. Their metal complexes are known to exhibit ligand-centered, reversible two-electron redox chemistry, thereby potentially serving as a two-electron reservoir.^28^ Previously, our group reported the isolation and structural characterization of boron formazanate complexes with the ligand in 3 different oxidation states.^29^ The most reduced form of the ligand was furthermore shown to be reactive toward electrophiles.^30^ We hypothesized that the electrochemical properties of formazanate and related ligands could also be useful in other (catalytic) redox transformations, such as the conversion of CO_2_ to CO.

Recently, we reported the preparation and characterization of Re­(I) carbonyl complexes bearing the protonated formazan ligand. In these species, the ligand coordinates the metal center to form a five-membered chelate ring (i.e., the *open* coordination mode)^31,32^ leaving a pendant NH arm that could be further functionalized or exploited as a proton-responsive group, providing a ligand scaffold that combines flexible coordination behavior, (reversible) redox-chemistry, and proton-relay characteristics. Herein, we describe the synthesis of a series of *fac*-Re­(CO)_3_ complexes with neutral formazan ligands as well as their deprotonated (formazanate) counterparts (Chart 1b). Electrochemical studies, both in the absence and presence of CO_2_, are reported and the speciation of the reduction products is investigated using chemical synthesis, infrared-spectroelectrochemistry and DFT studies.

## Results and Discussion

### Complex Synthesis

Starting from the previously reported neutral formazan complexes **H1** ^***Br***^ **–H4** ^***Br***^,^32^ the anionic rhenium formazanate complexes **[1** ^***Br***^ **]^−^–[4** ^***Br***^ **]^−^** were synthesized upon deprotonation of complexes **H1** ^***Br***^ **–H4** ^***Br***^ with NEt_3_ (Scheme 1a). The rhenium formazanate complexes exhibit a characteristic dark green color that distinguishes them from their dark purple conjugate acids. Although complexes **[1** ^***Br***^ **]^−^** **–[4** ^***Br***^ **]^−^** differ in the *para*-substituent attached to the central ring (R^1^ = H, Me, MeO, F), their spectroscopic characterization by FT-IR, NMR, and UV–vis indicates that they are similar in molecular structure and electronic environment at the metal (see Figures S1 and S2). Therefore, we focused our subsequent studies on the *F*-containing derivative **[4** ^***Br***^ **]** ^**–**^, since its ^19^F NMR resonance provides a convenient spectroscopic handle to track its reactivity. *In situ* addition of one equivalent of either tetraphenylphosphonium bromide [PPh_4_]­[Br] or bis­(triphenylphosphine)­iminium chloride [PPN]­[Cl] to a solution of the triethylammonium formazanate **[NHEt**_**3**_ **]­[4** ^***Br***^ **]** resulted in cation exchange to provide the corresponding ionic compounds **[PPh**_**4**_ **]­[4** ^***Br***^ **]** and **[PPN]­[4** ^***Br***^ **]**, respectively, which were isolated as solid microcrystalline materials. Treatment of **H4** ^***Br***^ with AgPF_6_ in refluxing acetonitrile followed by deprotonation with DABCO yielded the neutral complex **4** ^***MeCN***^ (Figure S14).

**Scheme 1:**
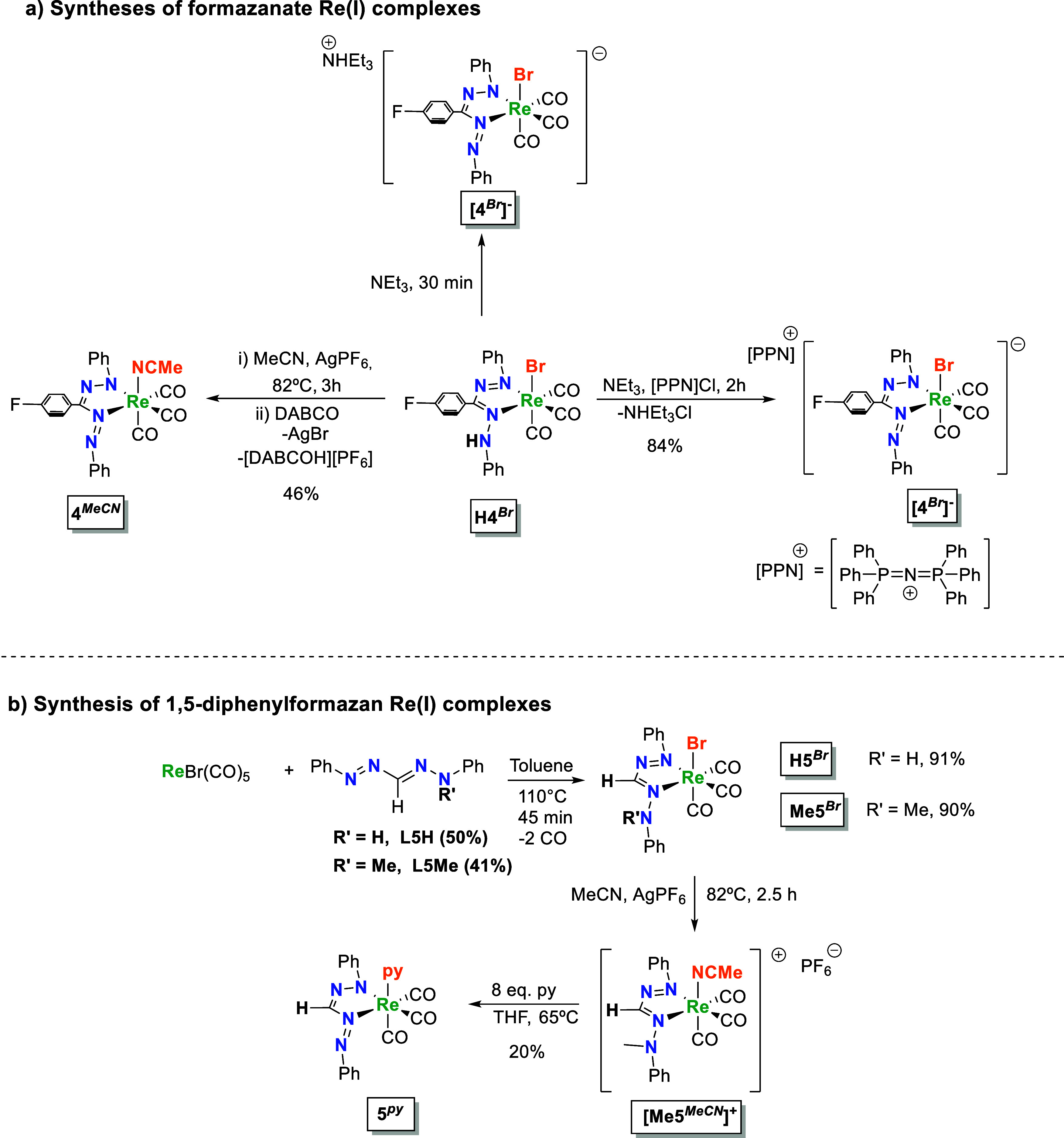
Synthesis of Re Complexes with (a) Formazanate and (b) Formazan Ligands

To assess the influence of the proton responsivity of the hydrazo/azo *N* atom we synthesized the *N*-methyl formazan Re­(I) complex **Me5** ^***Br***^ (Figures S17 and S18), by reacting equimolar amounts of the alkylated ligand *N*-methyl-1,5-diphenylformazan (**L5Me**) and ReBr­(CO)_5_. The change from a triarylformazan (**4**) to a diaryl derivative (**5**) is because of the follow-up reactivity of *N*-methyl triarylformazans: **L4Me** could not be isolated due to its rapid cyclization to the corresponding verdazyl compounds.^33^ Preparation of **L5Me** was carried out following the procedure reported by McConnachie and Neugebauer via methylation of 1,5-diphenyl formazan (**L5H**) in basic conditions.^33^ Following a similar route, compound **H5** ^***Br***^ was prepared (Figures S15 and S16). Both **H5** ^***Br***^ and **Me5** ^***Br***^ were obtained in good isolated yield (Scheme 1b). **Me5** ^***Br***^ was subsequently converted to **[Me5** ^***MeCN***^ **]** ^**+**^ by abstraction of the bromide ligand with AgPF_6_ in acetonitrile to provide a cationic complex with a weakly bound MeCN group that could potentially be displaced by other ligands (Figure S19). Accordingly, we attempted to effect ligand substitution by reacting a THF solution of **[Me5** ^***MeCN***^ **]** ^**+**^ with 8 equiv of pyridine at 65 °C. Unexpectedly, this led to demethylation of the ligand and isolation of the formazanate complex **5** ^***Py***^ instead (Figure S20). Although the isolated yield is only moderate (20–25%), **5** ^***Py***^ is the only Re-containing product that can be isolated from the mixture. The pathway that leads to this unusual transformation is currently unknown, but given the reaction conditions employed, it seems unlikely that conventional oxidative *N*-dealkylation is involved.^34–36^ Possibly, the formazanate ligand acts as a leaving group and the *N*-Me group is transferred to pyridine via nucleophilic substitution to form methylpyridinium and the formazanate complex **5** ^***Py***^.

### Characterization

Deprotonation of the neutral formazan species **H4** ^***Br***^ to give the formazanate complex **[4** ^***Br***^ **]** ^**–**^ upon treatment with NEt_3_ was confirmed by ^1^H NMR spectroscopy. The absence of the diagnostic resonance of the NH group at *ca*. 8.5 ppm indicated the reaction proceeded to completion. The IR spectra (in THF solution) of the anion **[4** ^***Br***^ **]** ^**–**^ are essentially identical regardless of the countercation [NHEt_3_]^+^, [PPN]^+^ or [PPh_4_]^+^, (Figures 1, S1, and S10), and exhibit three ν­(CO) bands as expected for a tricarbonyl complex with local *C*_3*v*_ symmetry. The carbonyl bands in the formazanate tricarbonyl anion are shifted to lower frequencies due to the increased donor ability of the anionic ligand compared to the protonated formazan complex (*e.g*. **H4** ^***Br***^ **=** 2031, 1955, 1918 cm^–1^; **[PPN]­[4** ^***Br***^ **]** = 2007, 1912, and 1880 cm^–1^, see Figure 1). Substitution of the axial bromide by a neutral acetonitrile ligand results in a blueshift of the CO vibrations (**4** ^***MeCN***^ = 2024, 1992­(br) cm^–1^, Figure S12). The infrared spectrum of the *N*-methyl formazan complex **Me5** ^***Br***^ exhibits carbonyl stretching vibrations (2031, 1952, 1915 cm^–1^, Figure 1) that are similar to the protonated parent compounds, making the methylated complex **Me5** ^***Br***^ a useful reference for comparison of reactivity studies (*vide infra*).

**Figure 1:**
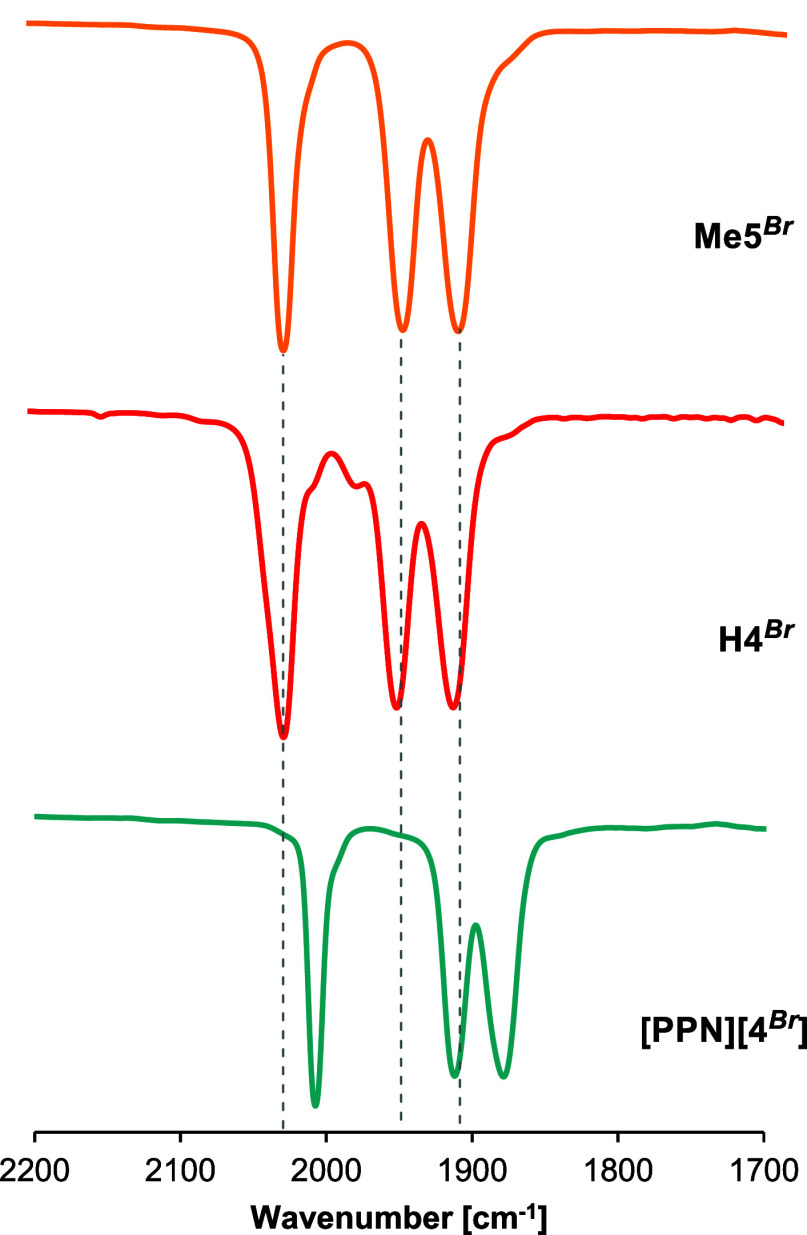
Comparative FT-IR spectra in THF solution between **H4** ^***Br***^ and **[PPN]­[4** ^***Br***^ **]**.

Analysis of the ^1^H NMR spectrum of the anionic complex **[4** ^***Br***^ **]** ^**–**^ showed that the formazanate ligand coordinates in an asymmetric (“*open*”) fashion, as two inequivalent *N*-Ph groups were found. The asymmetry of the ligand is retained at elevated temperatures (80 °C in toluene-*d*_8_). The absence of EXSY NMR cross-peaks between the two distinct sets of *N*-Ph resonances indicates that exchange via a symmetrical six-membered formazanate chelate does not occur at an appreciable rate, which contrasts with the behavior of zinc formazanate complexes (Figure S21).^31^ In the ^13^C NMR spectrum, the anionic Re complexes exhibit three CO resonances in the region 190–198 ppm, which are shifted downfield compared to their neutral precursors (185–193 ppm).

The (formal) negative charge at the ligand in the complexes **[4** ^***Br***^ **]** ^**–**^ and **4** ^***MeCN***^ substantially shifts the imine carbon upfield (155 ppm) compared to its protonated formazan analog (**H4** ^***Br***^: 164 ppm) (Figures S8 and S14 Supporting Information). For the complex with an alkylated formazan **Me5** ^***Br***^, the NMR signatures resemble those reported for the protonated formazan complexes, indicating that all compounds in this series have a similar coordination geometry, *i.e.*, an octahedral Re center with a five-membered formazan­(ate) chelate.

### Electronic Spectroscopy

The electronic spectrum of complex **[PPN]­[4** ^***Br***^ **]** was measured in THF at 25 °C (*c* ≈ 10^–5^ M). It exhibits a broad and intense absorption band at 605 nm (ε = 54 800 M^–1^ cm^–1^) (Figure 2, Table 1), which is red-shifted compared to the neutral precursor (**H4** ^***Br***^ = 515 nm). This feature is typical of metal complexes containing the formazanate ligand,^28^ and is assigned to the π–π* electronic transitions centered on the formazanate backbone. In the case of complex **H5** ^***Br***^ featuring a diarylformazan ligand, this band appears at higher energies (470 nm) (Figure 2). The low-energy absorption of **[4** ^***Br***^ **]** ^**–**^ undergoes negative solvatochromism and shifts to the red when the spectrum is measured in low polarity solvents (e.g., λ = 620 nm in toluene, Figure S13). Axial ligand exchange from anionic bromide to neutral acetonitrile does not perturb this π–π* transition, and it is observed at 611 nm in the acetonitrile complex **4** ^***MeCN***^ (Figure 2). The MeCN ligand proves to be labile, as within minutes after dissolution in THF, the main absorption band in **4** ^***MeCN***^ undergoes a shift to lower energy (Δλ ≈ 13 nm), suggesting displacement of MeCN by THF. In addition to the intense low-energy absorption, a less prominent band is observed in the visible range at 420–460 nm arising from MLCT excitations (Re­(d_π_) → formazanate (π*) based on TDDFT calculations (see Table S6). Similarly, the UV–vis spectrum of the formazanate complex **5** ^***Py***^ (Figure 2) exhibits the π–π* formazanate transition at 594 nm and another absorption at 387 nm that is assigned to ILCT involving the pyridine ligand (as TDDFT calculations indicate). In comparison to complex **H5** ^***Br***^, the low-energy absorption maximum of the *N*-methyl derivative **Me5** ^***Br***^ (449 nm) is blue-shifted by 21 nm (Figure 2).

**Figure 2:**
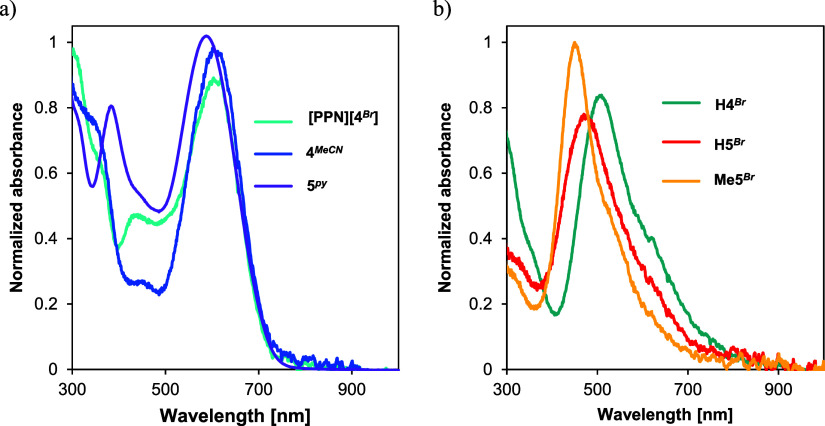
Absorption spectra of (a) formazanate complexes **[PPN]­[4** ^***Br***^ **]**, **4** ^***MeCN***^, **5** ^***Py***^, and (b) formazan complexes **H4** ^***Br***^, and **Me5** ^***Br***^ in THF.

**Table 1:** UV–Vis Spectrochemical Data in THF

compound	λ_max_ (nm)	ε (M^–1^·cm^–1^)	λ_max_ (nm)	ε (M^–1^·cm^–1^)
**[PPN][4** ^***Br***^ **]**	603	54 800	441	28 400
**[NHEt**_**3**_ **][4** ^***Br***^ **]** ^a^	615	26 300	462	13 600
**4** ^***MeCN***^	603	39 500	440	11 454
**4** ^***MeCN***^ ^a^	611	22 000	466	8 400
**H4** ^***Br***^	515	59 400		
**H5** ^***Br***^	470	22 600		
**Me5** ^***Br***^	449	40 700		
**5** ^***Py***^	594	27 716	387	13 090

a Measured in toluene.

The emission spectrum (λ_ex_ = 320 nm) of the deprotonated formazanate complex **[4** ^***Br***^ **]** ^**–**^ shows a weak band at 380 nm. The excited state decays in a biexponential manner (τ_1_ = 2.52 and τ_2_ = 8.72 ns under N_2_) and is hardly affected by the presence of O_2_ (τ_1_ = 2.28; τ_2_ = 8.71 ns in air), which suggests that it is not the typical ^3^MLCT emission observed in Re­(I) complexes with *N*-ligands but an emission from a singlet excited state (Figure S23).^37^ The emission is likely governed by the presence of low-lying π*-orbitals, highlighting the unique feature of the formazan ligand in such complexes. Similarly, the low-energy (singlet) emission features that are typical for 6-membered formazanate boron compounds^38,39^ are not observed here, which may be due to nonradiative pathways introduced by the less rigid geometry with a “free” uncoordinated terminal *N*-Ph group.^40^

### Structural Characterization

Attempts to obtain crystalline material from **[NHEt**_**3**_ **]­[4** ^***Br***^ **]** by ligand exchange with [PPN]^+^ or [PPh_4_]^+^ as countercations were unsuccessful. Ultimately, we were able to crystallize **[4** ^***Br***^ **]** ^**–**^ as decamethylcobaltocenium salt, [Co­(Cp*)_**2**_ **]­[4** ^***Br***^ **]**, upon slow evaporation of a THF solution. An X-ray structure determination confirmed **[4** ^***Br***^ **]** ^**–**^ to have a six-coordinate Re­(I) center with a formazanate ligand bound in the “*open*” fashion rendering a five-membered rhenacycle with the carbonyl ligands in a facial arrangement. The refinement showed substitutional disorder of the Br/CO fragments *trans* to each other, while the rest of the molecule is well-defined. The metrical parameters within the metallacycle indicate that the Re1–N1 bond length (2.112(4) Å) is similar to that in the neutral precursor **H4** ^***Br***^ (2.099(6) Å); however, it is shorter than Re–N­(azo) bond lengths (2.156(3) Å) in related species.^41^ The Re1–N3 bond length (2.192(4) Å) is typical for Re–N­(imine) bonds.^42^ Overall, the metrical parameters within the deprotonated formazanate backbone in **[4** ^*Br*^ **]** ^**–**^ are quite similar to that of the precursor, albeit a larger degree of delocalization is indicated by the equivalence of the N–N and C–N bonds, and the rhenium center is less displaced from the ligand plane (0.18 Å in **[4** ^***Br***^ **]** ^**–**^ vs 0.46 Å in **H4** ^***Br***^). The complexes with diphenylformazan or -formazanate ligands (**H5** ^***Br***^ **/Me5** ^***Br***^ and **5** ^***Py***^) are very similar to those bearing a triarylformazan­(ate) ligand, indicating that the substituent at the C7-atom or at the N4-atom of the ligand backbone has little influence on the structural features (Figure 3, Table 2).

**Figure 3:**
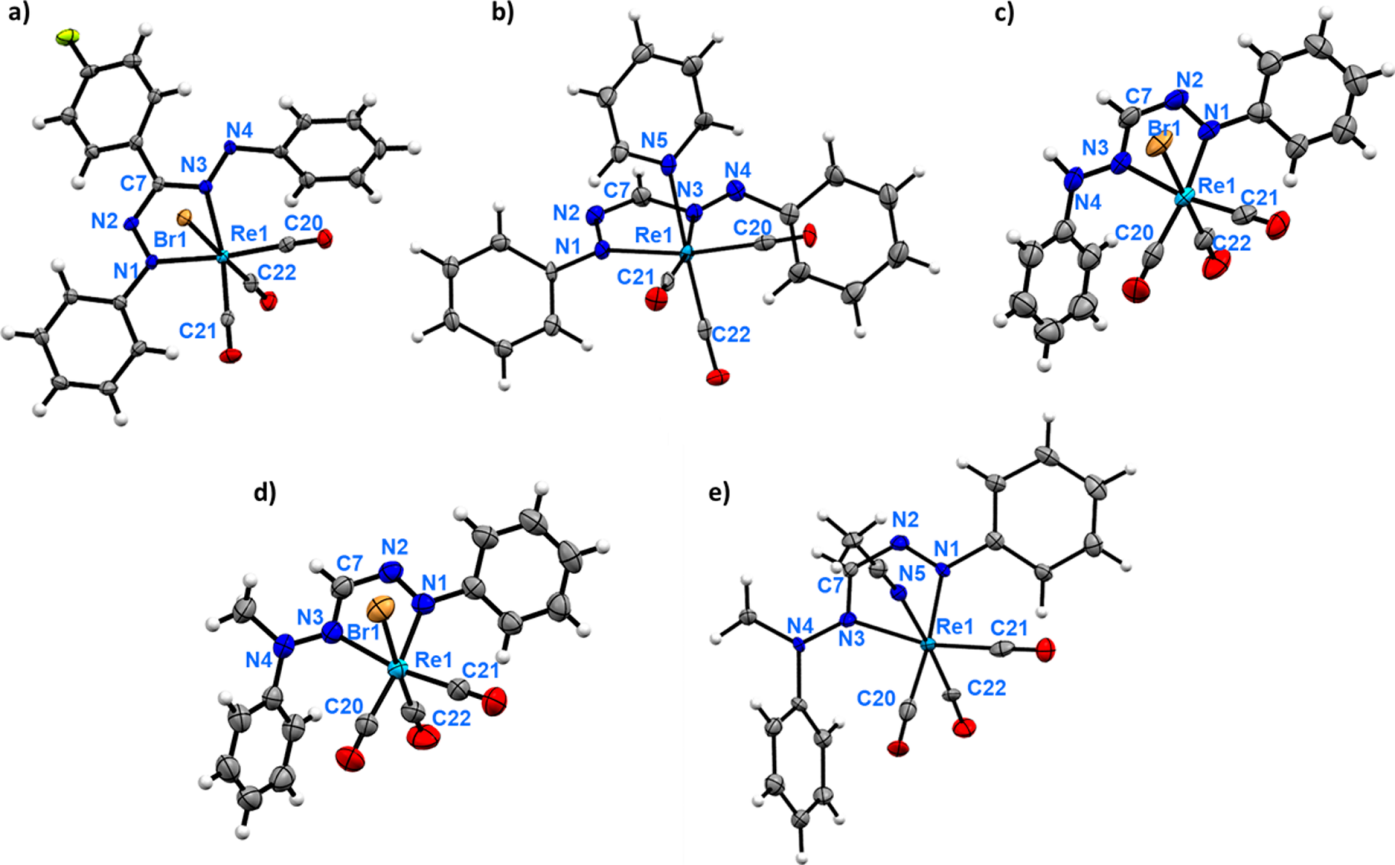
Molecular structure of (a) [Co­(Cp*)_**2**_ **]­[4** ^***Br***^ **]** (only the major disorder component is shown), (b) **5** ^***Py***^, (c) **H5** ^***Br***^, (d) **Me5** ^***Br***^ and (e) **[Me5** ^***MeCN***^ **]­[PF**_**6**_ **]** showing 50% probability ellipsoids; counterions in (a,e) omitted for clarity.

**Table 2:** Selected Metrical Parameters for [Co­(Cp*)_**2**_ **]­[4** ^***Br***^ **]**, **5** ^***Py***^, **H4** ^***Br***^, **H5** ^***Br***^, **Me5** ^***Br***^ and **[Me5** ^***MeCN***^ **]­[PF**_**6**_ **]** (Bond Lengths in Å)

	formazanate complexes		formazan complexes			
	[Co(Cp*)_**2**_ **][4** ^***Br***^ **]**	**5** ^***Py***^	**H4** ^***Br***^ ^a^	**H5** ^***Br***^	**Me5** ^***Br***^	**[Me5** ^***MeCN***^ **][PF**_**6**_ **]**
Re1–Br1	2.6000(14)^b^		2.6236(7)	2.6279(8)	2.6191(3)	
Re1–N1	2.112(4)	2.144(5)	2.099(6)	2.117(5)	2.124(2)	2.140(8)
Re1–N3	2.192(4)	2.186(5)	2.185(5)	2.161(4)	2.186(2)	2.201(9)
Re1–C20	1.939(5)	1.959(7)	1.955(6)	1.960(8)	1.941(3)	1.931(1)
Re1–C21	1.912(5)	1.916(7)	1.919(5)	1.936(6)	1.915(3)	1.909(9)
Re1–C22	1.887(13)^b^	1.899(6)	1.918(6)	1.917(5)	1.913(3)	1.93(1)
N1–N2	1.300(6)	1.325(7)	1.291(7)	1.293(6)	1.292(3)	1.30(1)
C7–N2	1.341(7)	1.32(1)	1.382(7)	1.347(8)	1.369(4)	1.35(1)
C7–N3	1.370(6)	1.37(1)	1.319(8)	1.316(8)	1.309(4)	1.32(1)
N3–N4	1.301(7)	1.30(8)	1.324(8)	1.347(7)	1.354(3)	1.34(1)

a Values taken from *Inorg. Chem*., **2022,** *61*, 13532–13542.

b These bond lengths were restrained in the refinement, values shown are for the major disorder component. The large standard uncertainty in these numbers means that a comparison with the other compounds is not meaningful.

### Electrochemical Characterization

Cyclic voltammograms under N_2_ were recorded at 0.05 V/s in a 0.1 M [NBu_4_]­[PF_6_] acetonitrile solution containing 1 mM of the analyte. Representative CVs are shown in Figures 4 and 5, with (peak) potentials listed in Table 3. Unless otherwise stated, the potentials were referenced internally versus ferrocene (Fc^+/0^). We first discuss the electrochemistry of the neutral methyl formazan derivative **Me5** ^***Br***^ since it exhibits the cleanest voltammetry data and provides a useful reference for the other compounds. In the first scan of a cyclic voltammetry experiment, three successive one-electron reductions were identified for **Me5** ^***Br***^ when scanning to negative potentials with peak potentials at *I*′ = −0.53, *II*′ = −0.84, and *III*′ = −2.83 V. The redox events *II* and *III* are *quasi*-reversible processes, but the ratio of cathodic and anodic peak currents is well below unity for the redox peak *I*, which suggests that a chemical step occurs after the initial electron transfer. On the reverse scan, besides the oxidations *I*″, *II*″ and *III*″ coupled to these reductions, an additional wave *IV*″ is observed (−0.38 V), which must arise from a new species formed under electrochemical conditions. Its corresponding reduction peak *IV*′ is detected only in the second cycle (Figure 4a). Notably, the initial two reductions appear at much less negative potentials than in Lehn’s Re­(CO)_3_(bpy)Cl complex (*cf.* −1.75 vs Fc^+/0^, −2.16 V vs SCE),^43,44^ indicating distinct ligand reduction for those processes, because the formazan ligand can be reduced already at moderate potentials.

**Figure 4:**
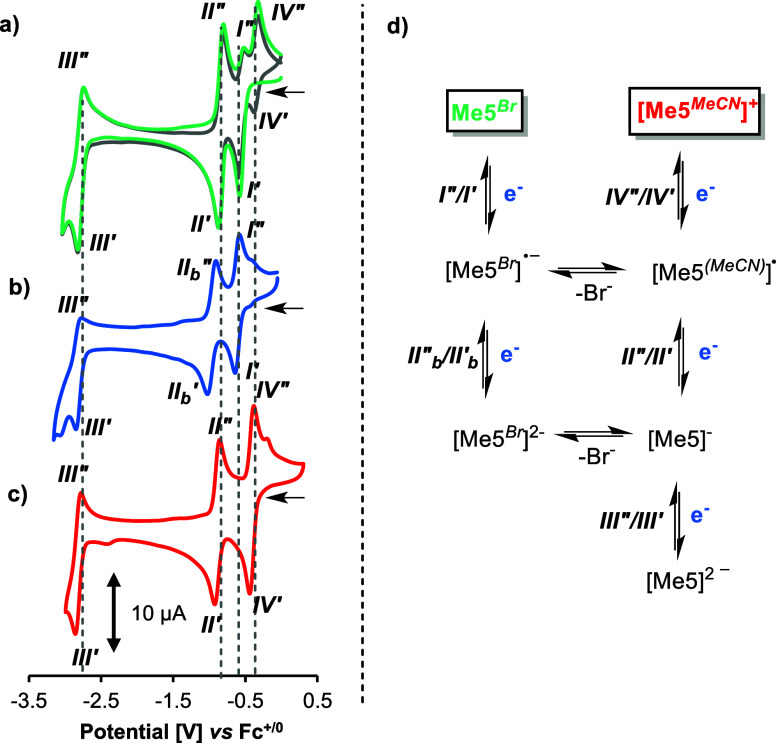
Voltammograms of complex (a) **Me5** ^***Br***^, (b) **Me5** ^***Br***^ + 50 equiv of Bu_4_NBr and, (c) **[Me5** ^***MeCN***^ **]­[PF**_**6**_ **]** in acetonitrile at 0.05 V/s. The gray trace in (a) corresponds to the second cycle. (d) Proposed pathway for the reduction of methylformazan complexes.

**Figure 5:**
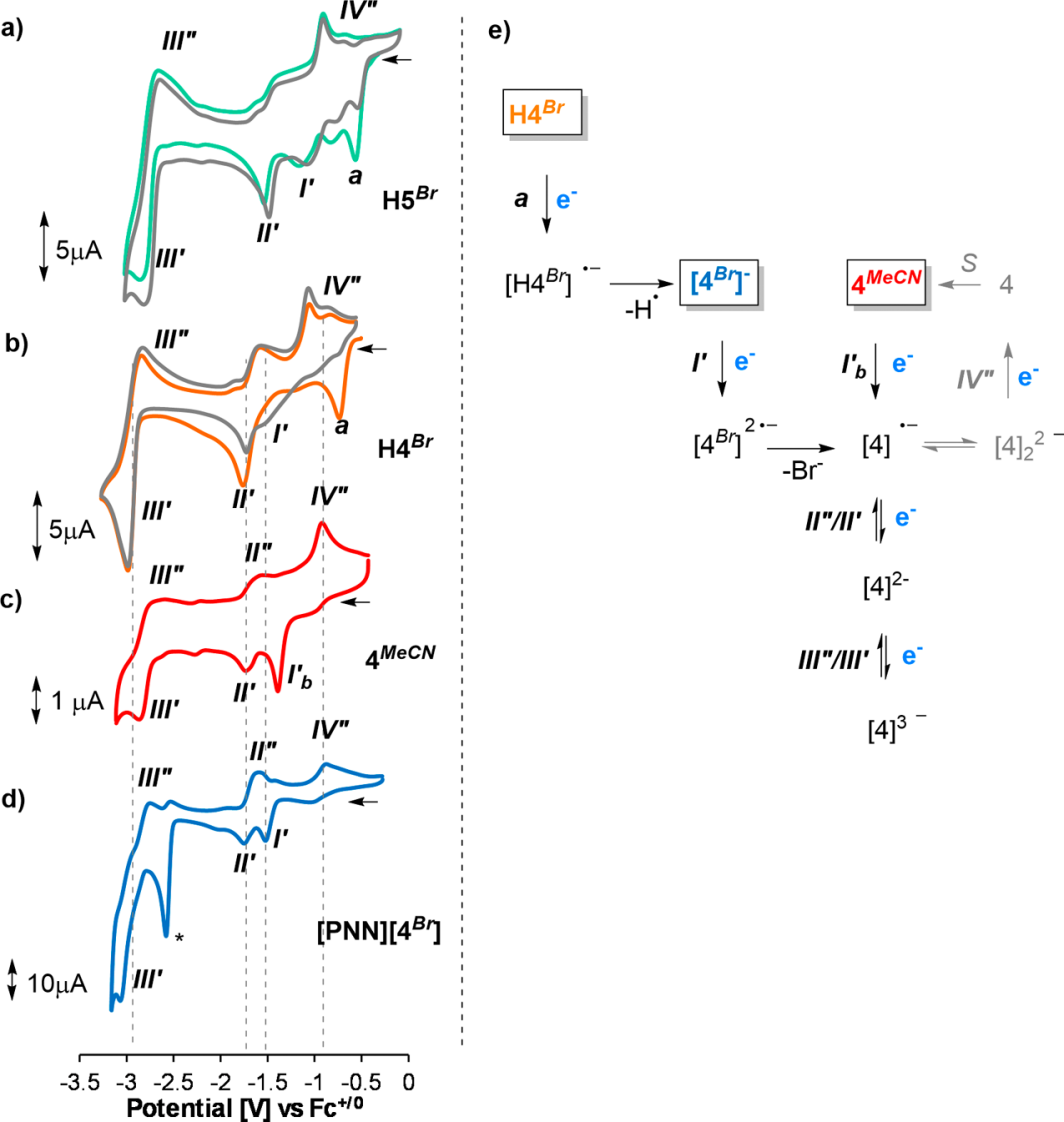
Voltammograms of complexes (a) **H5** ^***Br***^, (b) **H4** ^***Br***^, (c) **4** ^***MeCN***^ and (d) **[PPN]­[4** ^***Br***^ **]** in acetonitrile at 0.05 V/s. The gray trace in (a) corresponds to the second cycle. (*) assigned to the reduction of the [PPN]^+^ cation. (e) Proposed pathway for the reduction of formazanate and protonated formazan complexes.

**Table 3:** Electrochemical Data for **Me5** ^***Br***^, **[Me5** ^***MeCN***^ **]­[PF**_**6**_ **]**, **H5** ^***Br***^, **H4** ^***Br***^, **[PPN]­[4** ^***Br***^ **]** and **4** ^***MeCN***^

	redox waves/[V] vs Fc^+/0^			
compound	***I***	***II***	***III***	***IV***
**Me5** ^***Br***^	*I*″/*I*′ = −0.59	*II*″/*II*′ = −0.87	*III*″/*III*′ = −2.80	*IV*″/*IV*′ = −0.38
**[Me5** ^***MeCN***^ **][PF**_**6**_ **]**		*II*″/*II*′ = −0.89	*III*″/*III*′ = −2.81	*IV*″/*IV*′ = −0.41
**H5** ^***Br***^	*a* = −0.53	*II*′ = −1.52	*III*″/*III*′ = −2.78	*IV*″ = −0.66
**H4** ^***Br***^	*a* = −0.73	*II*′ = −1.70	*III*″/*III*′ = −2.89	*IV*″ = −0.86
**[PPN][4** ^***Br***^ **]**	*I*′ = −1.51	*II*″/*II*′ = −1.67	*III*″/*III*′ = −2.90	*IV*″ = −0.88
**4** ^***MeCN***^	*I*′ = −1.40	*II*_b_^″^/*II*_b_^′^ = −1.65	*III*″/*III*′ = −2.80	*IV*″ = −0.87

The chemical step that follows the one-electron reduction (*I*′) of **Me5** ^***Br***^ likely involves axial ligand dissociation, as has been reported in related systems.^2,17,45^ Loss of Br^–^ from the electrochemically generated radical anion (**[Me5** ^***Br***^ **]** ^**•–**^) in our system is supported by the observation that the peak current ratio (*I*′/*I*″) is changed significantly in the presence of added Bu_4_NBr (50 equiv), which indicates the equilibrium **[Me5** ^***Br***^ **]** ^**•–**^ ⇌ **Me5** ^**•**^ + Br^–^ (Figure 4b). Based on the relatively small (∼300 mV) separation between the reduction waves *I*′ and *II*′, we propose that the second (*quasi*-reversible) reduction wave is due to electron transfer to **Me5** ^**•**^ forming the closed-shell anion **[Me5]** ^**–**^. In the presence of excess of bromide, a slight cathodic shift (*ca*. 0.15 V) of this feature suggests that also this species is in equilibrium with a bromide-coordinated species (**[Me5** ^***Br***^ **]** ^**2–**^). The reduction at *III*′ generates the triply reduced complex **[Me5]** ^**2–**^. On the reverse scan, the oxidations coupled to these reductions were observed at *I*″ = −0.60, *II*″ = −0.89, and *III*″ = −2.73 V. By carrying out CV experiments in select potential windows (Figure S24), we confirmed that the additional redox waves *IV* are related to the species that is generated by reduction at *I*′ (−0.53 V) and we thus assign this to the bromide-free **Me5** ^**•**^/**[Me5]** ^**+**^ couple (or a solvated analogue). This is further corroborated by the CV analysis of the independently prepared *cationic* complex **[Me5** ^***MeCN***^ **]­[PF**_**6**_ **]** (Figure 4c), the voltammetry of which indeed shows the redox waves *IV*″/*IV*′ in addition to *II*″/*II*′ and *III*″/*III*′ (but not *I*′ or *I*″). This confirms that the two- and three-electron reduction reactions observed in the CV of **Me5** ^***Br***^ are due to bromide-free species.

Next, we evaluated the electrochemistry of neutral formazan complex **H5** ^***Br***^ (Figure 5a), which has an *N*-H group instead of *N*-Me. Its cyclic voltammogram shows that the first reduction of this complex occurs at a cathodic peak potential of −0.6 V, but no return wave is observed, also at higher scan rates (up to 1 V/s), indicating this reduction to be irreversible. A scan to more negative potentials has additional peaks at −1.1 V and −1.5 V (labeled as *II*′ and *III*′, respectively). The electrochemical profile of Re complexes bearing the protonated formazan ligand is similar in all cases (*e.g.*, **H4** ^***Br***^ and **H5** ^***Br***^, see Figure 5). The pronounced differences between complexes with *N*-Me (**Me5** ^***Br***^) and protonated (*N*-H; **H4** ^***Br***^ and **H5** ^***Br***^) formazan ligands were unexpected, so we decided to examine the reduction chemistry of **H4** ^***Br***^ via a preparative scale synthesis with a chemical redox reagent. The addition of one equivalent of Co­(Cp*)_2_ into a THF solution of **H4** ^***Br***^ resulted in an immediate color change from purple to dark blue. Analysis of the reaction mixture by FT-IR showed that the resulting product exhibits ν­(CO) bands at 2008, 1912, and 1880 cm^–1^. The color change and the IR spectral features are reminiscent of the anionic formazanate complex **[4** ^***Br***^ **]** ^**–**^, (Figures S7 and S8), suggesting that 1-electron reduction of **H4** ^***Br***^ is followed by hydrogen atom loss (reductive NH bond cleavage, Figure 5e). The identity of the product as the formazanate complex **[4** ^***Br***^ **]** ^**–**^ (as the [Co­(Cp*)_2_]^+^ salt) was confirmed by UV–vis and ^19^F NMR spectroscopy, as well as X-ray crystallography (*vide supra*). Similar reductive deprotonation was reported for metal complexes bearing ligands containing proton-responsive groups, such as dihydroxy-bipyridine^23,46^ and imidazole fragments.^47^ This transformation is fast on the time scale of the cyclic voltammetry experiment, and all subsequent redox processes observed in the CV of **H4** ^***Br***^ are due to electrochemically generated **[4** ^***Br***^ **]** ^**–**^. This is corroborated by a FT-IR spectroelectrochemistry study (*vide infra*), where we observe the same species regardless of whether the starting material is **H4** ^***Br***^ or **[4** ^***Br***^ **]** ^**–**^ (Figure S29).

The CVs of complexes with deprotonated formazanate ligands, *i.e.*, **[PPN]­[4** ^***Br***^ **]** and **4** ^***MeCN***^, are included for comparison purposes in Figure 5. The correspondence between these compounds indicates that in all cases the speciation under these electrochemical conditions is similar: also for these compounds, bromide dissociation leads to the radical anion **[4]** ^**•–**^ as a common intermediate regardless of whether or not the starting material contains a bromide ligand. A similar electrochemical profile is observed when the CV of **4** ^***MeCN***^ is recorded in DMF, evidence that further supports the formation of the non-solvated one-electron radical **[4]** ^**•–**^ (Figure S28a). The electrochemical behavior is not affected by the formazanate substitution pattern or the nature of the axial ligand: the CV of the pyridine adduct **5** ^***Py***^ is essentially the same as that of **4** ^***MeCN***^ (Figure S28b).

Analogous to what is observed for **Me5** ^***Br***^ above, further reduction of **[4]** ^**•–**^ is indicated by the redox waves at −1.67 V (*II*′/*II*″) and −2.80 V (*III*′/*III*″), which generate the corresponding di- and trianionic complexes **[4]** ^**2–**^ and **[4]** ^**•3–**^, respectively. Finally, the anodic peak *IV*″ is only observed once the potential is swept past peak *I*′ and is ascribed to oxidation of a dimeric intermediate (*e.g.*, **[4]**_**2**_^**2–**^, or a mixed-valent species such as **[4]**_**2**_^**•–**^), in analogy to the dimerization observed in the reduction chemistry of ReCl­(CO)_3_(bpy).^48^

### Spectroelectrochemistry

To gain more insight into the nature of the intermediates generated upon reduction, IR spectroelectrochemistry measurements were performed under a nitrogen atmosphere. In acetonitrile, **Me5** ^***Br***^ exhibits three ν­(CO) bands at 2032, 1950, and 1919 cm^–1^ which fade at the potential of the first reduction *I*′, and are replaced by two new sets of CO vibrations (2019 and 1914­(br) cm^–1^ (major); 2001 and 1876­(br) cm^–1^ (minor)) (Figure 6; red trace). This indicates that a mixture of two Re carbonyl species is formed, which both remain visible also when the potential is passed beyond the first reduction wave. The major species is assigned to the solvato-radical **Me5** ^***MeCN*•**^. The same two sets of IR bands were observed for the products of a chemical reduction experiment, when either the neutral complex **Me5** ^***Br***^ or the cationic acetonitrile complex **[Me5** ^***MeCN***^ **]­[PF**_**6**_ **]** were treated with Co­(Cp*)_2_ as chemical reducing agent (Figure S30). The ν­(CO) bands of the minor product (2001, 1876 cm^–1^), are assigned to the radical anion **Me5** ^**•**^ based on DFT calculations (see below).

**Figure 6:**
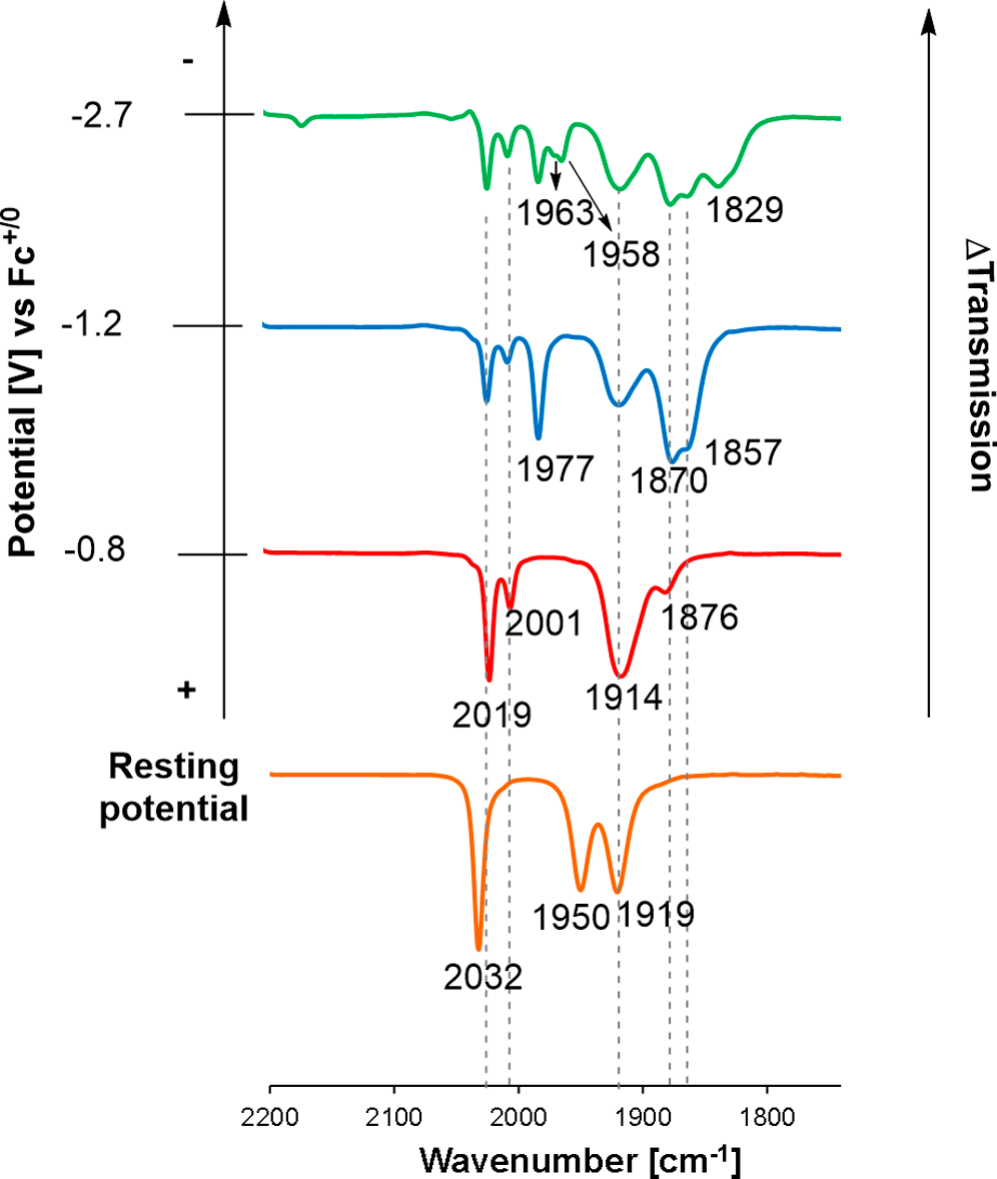
Infrared spectroelectrochemistry of complex **Me5** ^***Br***^ in acetonitrile.

When the potential of the second reduction ***II*′** is reached, three carbonyl bands of similar intensity rise at 1977, 1870, and 1857 cm^–1^, which we attribute to the two-electron reduced complex **[Me5]** ^**–**^ (Figure 6, blue trace). This assignment is further supported by the appearance of the same IR bands in a solution of **Me5** ^***B*** *r*^ that was treated with two equivalents of Co­(Cp*)_2_ (Figure S30). In the literature, the 2-electron reduction chemistry of the archetypical complex *fac*-ReCl­(CO)_3_(bpy) has been reported to shift the high-energy carbonyl band by 74 cm^–1^.^11^ The shift that we observe here is smaller (55 cm^–1^), which we ascribe to the reductions being primarily ligand-based.^27–29^ Costentin and Chardon-Noblat reported similar observations when the bipyridine scaffold was furnished with electron-withdrawing groups, making reductions more ligand-centered which led to a smaller shift in ν­(CO).^49^ The anion **[Me5]** ^**–**^ is the major species in the mixture until the potential reaches values more negative than −2.6 V. At that point, two new ν­(CO) bands at 1963, 1958, and 1829 cm^–1^ start to appear, which we tentatively assign to the 3-electron reduced complex **[Me5]** ^**2–**^ (Figure 6, green trace).

We subsequently investigated the reduction products of the formazanate species **[4** ^***Br***^ **]** ^**–**^ and **4** ^***MeCN***^. Both these compounds lead to very similar FT-IR spectra in the spectroelectrochemistry experiment (see Figure S29), in accordance with the conclusion based on cyclic voltammetry that these lead to the common initial intermediate **[4]** ^**•–**^ (*vide supra*) (Figure 7). Upon reaching the potential of the reduction peak *I*′, the formation of two new species is indicated by the appearance of high-energy carbonyl bands at 1995 and 1976 cm^–1^ that are assigned to a symmetric stretching mode (A_1_ in a generic *fac*-tricarbonyl complex of *C*_3*v*_ symmetry). The asymmetric CO stretching modes are found at 1894, 1868, and 1858 cm^–1^, but the overlap makes a definite assignment difficult. The two species are assigned as the radical anion **[4]** ^**•–**^ and its dimerization product **[4]**_**2**_^**2–**^. The formation of such dimers has precedent in the literature,^43,48,51–53^ and is further supported by DFT computations (*vide infra*). Attempts to chemically synthesize and isolate the dimeric species have been so far unsuccessful. At the potential of peak *II*′, two ν­(CO) bands arise at 1958 and 1830­(br) cm^–1^ that we assign to the 2-electron reduced complex **[4]** ^**2–**^. The shift in the carbonyl stretching frequency (52 cm^–1^) in these formazanate complexes is similar to that found for the methylformazan species, indicating ligand-centered reductions also for the formazanate complexes.

**Figure 7:**
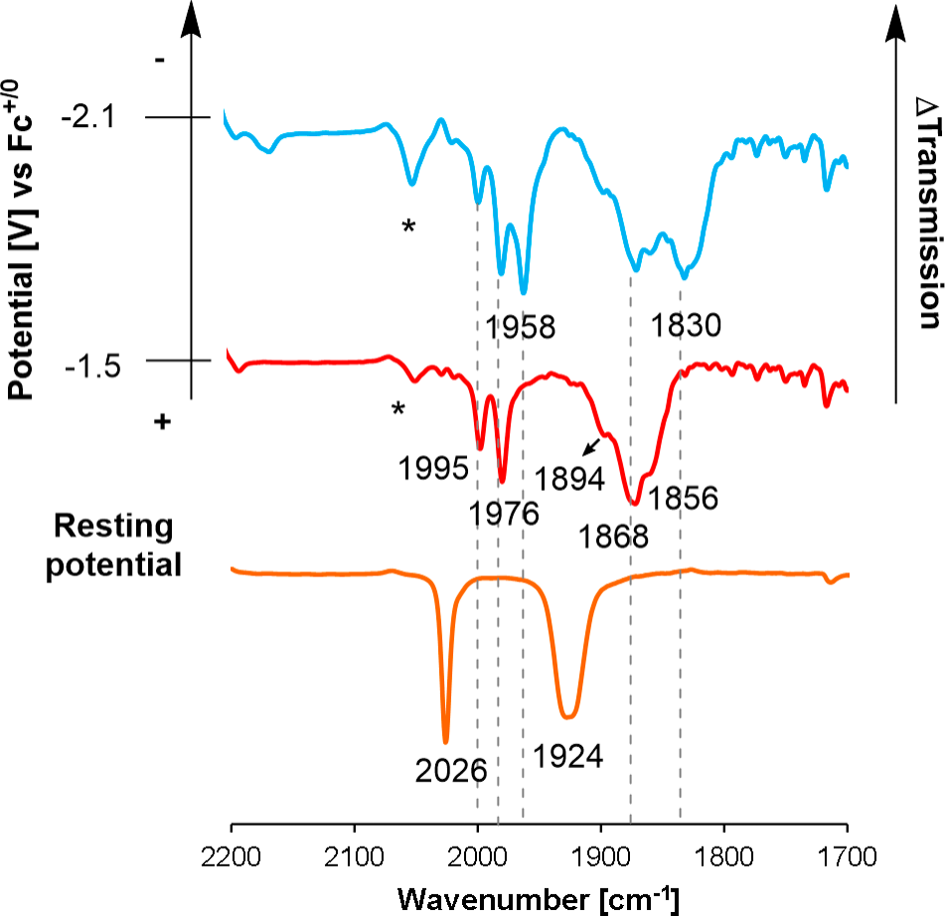
Infrared spectroelectrochemistry of complex **4** ^***MeCN***^ in acetonitrile.

### Density Functional Theory

To gain more information about the electronic structure of the complexes described herein, DFT calculations were carried out. Geometry optimizations were run at the MN15L^54^/def2-tzvp^55^ level of theory, using the crystallographic coordinates of **Me5** ^***Br***^ and **[4** ^***Br***^ **]** ^**–**^ for the formazan and formazanate complexes, respectively, as starting point. Analytical frequency calculations confirmed that the resulting geometries were minima on the potential energy surface (no imaginary frequencies). A table comparing empirical and computed (after scaling with the appropriate factor of 0.9578 ^50^) CO stretching frequencies is presented in the Supporting Information (Table S4).

The optimized structure of **Me5** ^***Br***^ exhibits metrical parameters that agree with those found in the X-ray structure (Table S3). The computed CO vibrations (2022, 1960, 1924 cm^–1^; average = 1969 cm^–1^) are also comparable to the experimental values (2032, 1950, 1919 cm^–1^; average = 1967 cm^–1^). The HOMO is mainly of metal character, whereas the LUMO has a mixed metal–ligand nature (Figure 8a). Unrestricted DFT calculations were carried out on the structure of the one-electron reduced complexes **[Me5** ^***Br***^ **]** ^**•–**^, **Me5** ^**•**^ and **Me5** ^***MeCN*•**^. In this series, the calculated CO frequencies for the bromido species occur at lower frequencies compared to the neutral radicals (**[Me5** ^***Br***^ **]** ^**•–**^ = 1986, 1908, 1880 cm^–1^ vs **Me5** ^**•**^ = 2009, 1936, 1920 cm^–1^, **Me5** ^***MeCN*•**^ = 2011, 1932, 1929 cm^–1^). Binding of acetonitrile to the radical **Me5** ^**•**^ was calculated to be favorable (Δ*G* = −10.9 kcal/mol in the gas phase), suggesting that the adduct **Me5** ^***MeCN*•**^ is the species that is experimentally observed. Thus, the data is consistent with the formation of an equilibrium mixture of the non-solvated anion **Me5** ^**•**^ (exp: 2001, 1876­(br) cm^–1^) and the neutral acetonitrile complex **Me5** ^***MeCN*•**^ (exp: 2019, 1914­(br) cm^–1^). Spin density plots (isovalue = 0.01) of these radicals indicate that the unpaired electron density is mainly centered on the formazan metallacycle (Figure 9a). The geometry of the two-electron reduction product, **[Me5]** ^**–**^, was subsequently optimized in the open- and closed-shell singlet as well as triplet state. The broken-symmetry calculation converged on the closed-shell singlet, and this was found to be lower in energy than the triplet state by 37 kcal/mol. Also for this complex, the computed CO frequencies (1969, 1883, 1876 cm^–1^; average = 1909 cm^–1^) are in good agreement with the two-electron reduction product observed in the spectroelectrochemistry data (1977, 1869, 1857­(br) cm^–1^; average = 1901 cm^–1^).

**Figure 8:**
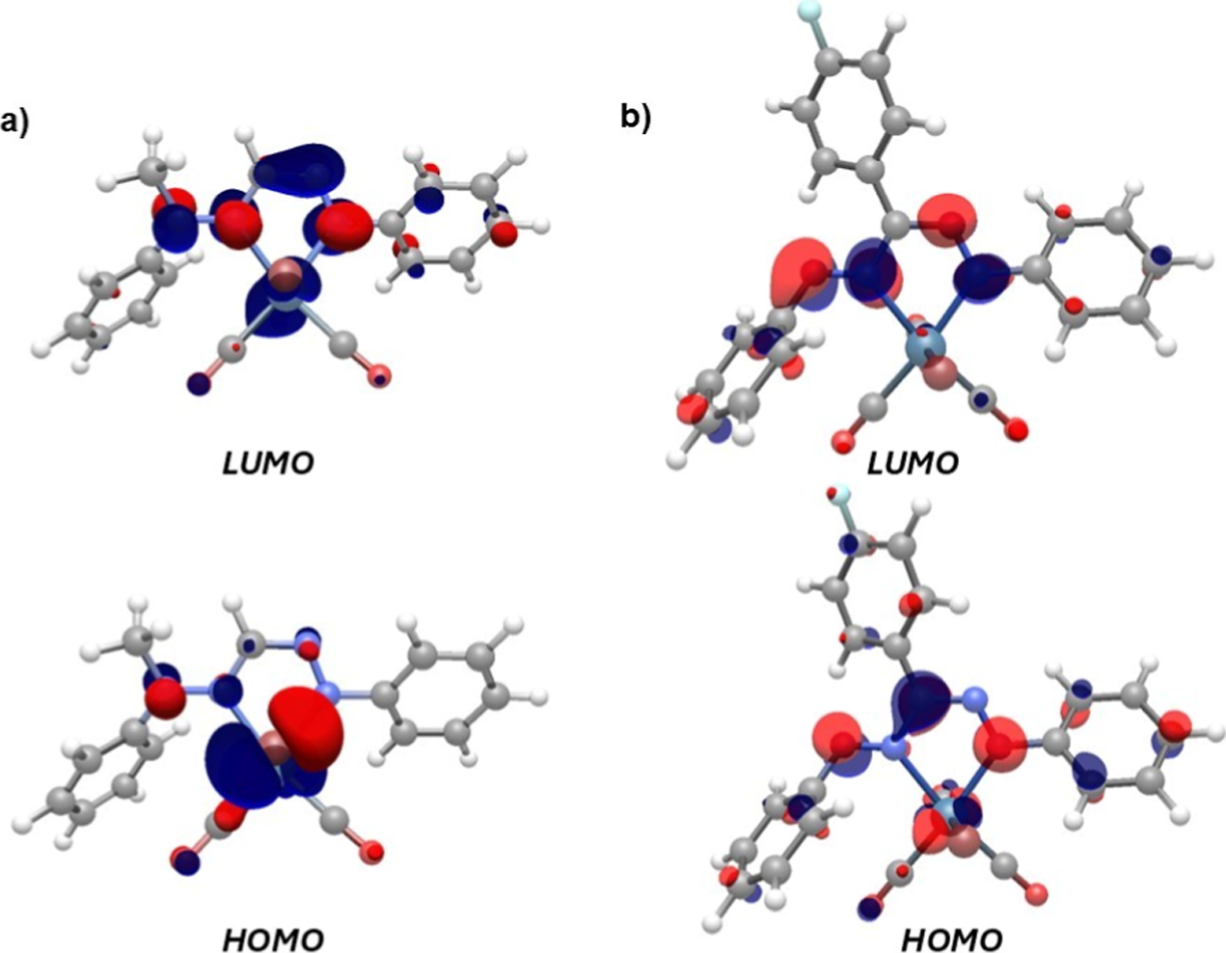
Frontier orbitals (isovalue = 0.05) of (a) **Me5** ^***Br***^ and (b) **[4** ^***Br***^ **]** ^**–**^.

**Figure 9:**
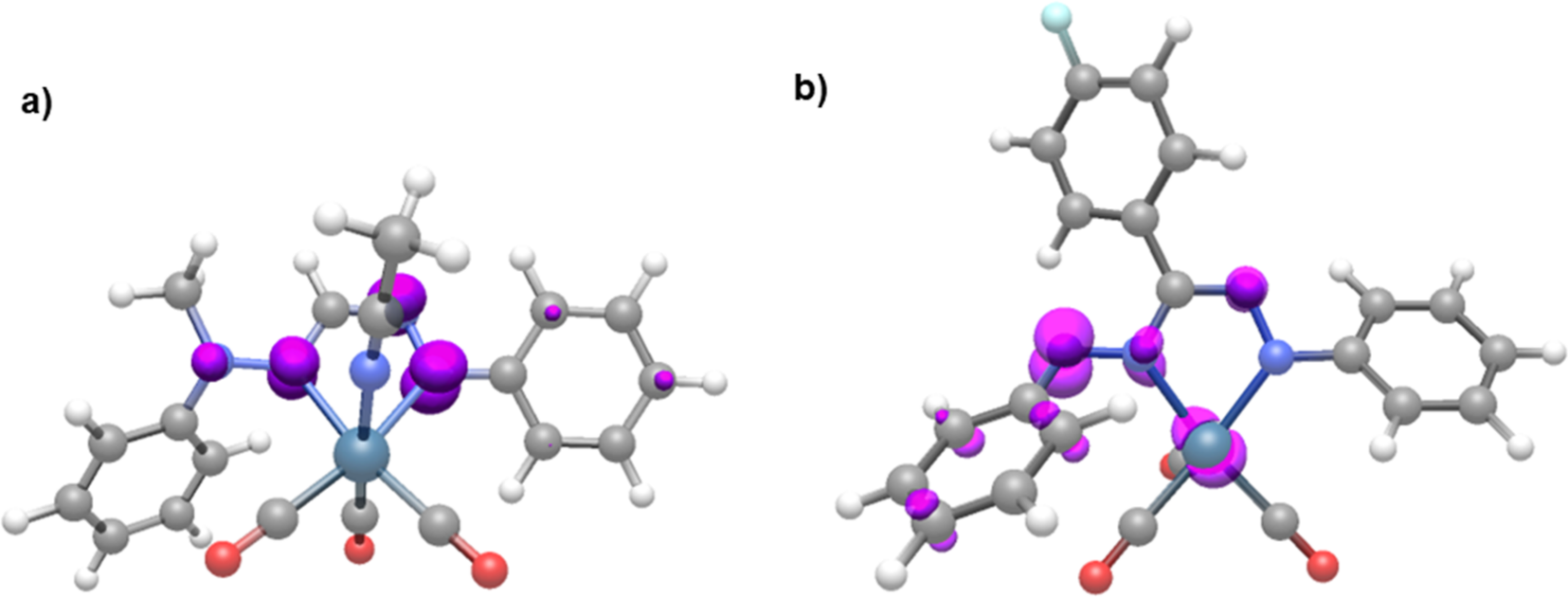
Spin density plots (isovalue = 0.01) of the one-electron reduced complexes (a) **Me5** ^***MeCN*•**^ and (b) **[4]** ^**•–**^.

Similar calculations were carried out for the formazanate complex **[4** ^***Br***^ **]** ^**–**^, which reproduced the experimental structure well and showed a satisfactory match with the CO stretching vibrations (DFT: 2010, 1912, 1884 cm^–1^; average = 1935 cm^–1^ vs experimental: 1997, 1915, 1911 cm^–1^; average = 1941 cm^–1^). Analysis of the frontier orbitals indicates that the HOMO is mainly localized on the formazanate backbone with a small contribution of the metal center (Re d_π_ orbital), while the LUMO has ligand π*-character primarily (Figure 8b).^4^ Unrestricted DFT calculations were performed on the one-electron reduction product of **[4** ^***Br***^ **]** ^**–**^, *i.e.*, the 17-electron radical anion **[4]** ^**•–**^, for which the computed carbonyl frequencies (1978, 1892, 1887 cm^–1^) are in good agreement with the experimental values. Spin density calculations indicate that the unpaired electron is delocalized over the metal and formazanate ligand (Figure 9b); hence, the first reduction is not a purely ligand-based process, but it also involves the metal due to the covalent nature of the bonding. Since reduced formazanate complexes have thus far only been obtained with the ligand bound in a six-membered chelate ring, we also computed this alternative coordination mode, but at this level of theory the five-membered chelate ring is favored by 4.6 kcal/mol, likely due to the larger size of the Re ion, thus preferring 5-membered chelating rings with larger bite angles.^56^ For the putative doubly reduced species **[4]** ^**2–**^, we optimized the structure using a broken-symmetry approach to determine whether the open or closed-shell singlet is favored. The results showed that both possible conformations of the formazanate ligand, the five- and six-membered chelate (**[4]**_***a***_^**2–**^, **[4]**_***b***_^**2–**^, respectively), are most stable in the closed-shell singlet state (the triplet state is ∼30 kcal/mol higher; see Figure S38 for the structures). The difference in energy between the two different ligand conformations is minor (0.50 kcal/mol), and in the absence of further experimental verification, we refrain from making a structural assignment. The computed carbonyl frequencies are similar for both formazanate binding modes (**[4]**_***a***_^**2–**^ = 1950, 1860, 1837 cm^–1^; **[4]**_***b***_^**2–**^ = 1940, 1841, 1837 cm^–1^), and match well with the data from IR spectroelectrochemistry.

Based on the available experimental and computational data, we propose the speciation depicted in Scheme 2 for the reduction chemistry of the Re complexes described here. Compounds with a neutral formazan ligand, either with *N*-H (**H4** ^***Br***^/**H5** ^***Br***^) or *N*-Me group (**Me5** ^***Br***^), are reduced at relatively mild potentials (∼−0.6 V vs Fc^+/0^) to the corresponding radical anions. In case of the *N*-H formazan species, this reduction results in rapid conversion to the formazanate analogues (**[4** ^***Br***^ **]** ^**–**^ and **[5** ^***Br***^ **]** ^**–**^) by hydrogen-atom loss. The *N*-Me derivative, on the other hand, is stable and can be further reduced to the closed-shell species **[Me5]** ^**–**^ in which the azo-imine (N=N–C=N) fragment of the neutral formazan ligand is converted to a dianionic imine-diamido (N–N=C–N), similar to that observed in the 2-electron redox chemistry of α-diimines.^57–59^ The deprotonated formazanate complexes are redox-active at more negative potentials, but similarly lead to ligand-based reductions.

**Scheme 2:**
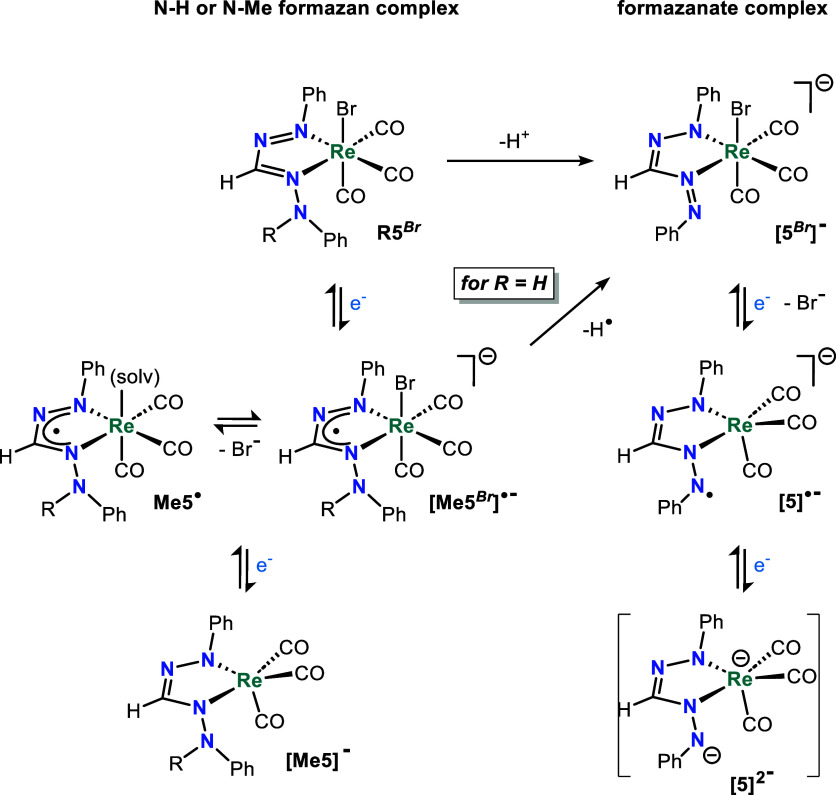
Proposed Pathways of the Reduction Chemistry of Formazan/Ate Re­(I) Complexes

### Electrochemistry and Spectroscopy in the Presence of CO_2_

We investigated the reactivity of both formazanate and alkylformazan Re­(I) complexes toward CO_2_, as has been extensively explored for other Re­(I) carbonyl complexes bearing bidentate nitrogen-based ligands. The cyclic voltammograms of **Me5** ^***B*** *r*^ and **4** ^***MeCN***^ were recorded upon CO_2_ saturation in the absence and the presence of phenol (Figure 10), which has been shown to be an efficient Brønsted acid in CO_2_ electroreduction chemistry.^60^ Control experiments that monitored the reaction between phenol and formazanate Re complexes by NMR spectroscopy indicated that it is not a sufficiently strong acid to protonate the ligand under these conditions.

**Figure 10:**
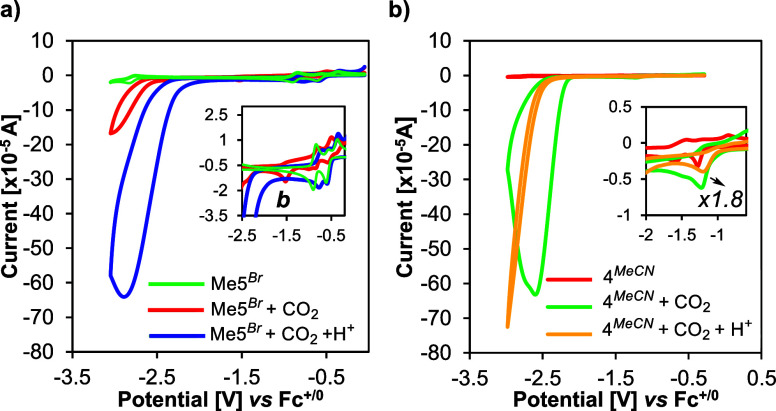
Cyclic voltammograms under CO_2_ with and without 5% phenol at 50 mV/s for (a) **Me5** ^***Br***^ and (b) **4** ^***MeCN***^. The peak labeled as *b* in (a) suggests that CO_2_ interacts with a reduced form of **Me5** ^***Br***^.

The CV of **Me5** ^**Br**^ under CO_2_ (Figure 10a) displays a subtle anodic shift (40–50 mV) of the reduction waves *I*′ (−0.84 V) and *II*′ (−0.57 V) accompanied by the formation of a new reduction peak *b* at −1.52 V; however, no current increase was detected at these potentials, and it is only when reaching more negative values (−2.6 V) that the current rises (8.6 times) at a potential close to the reduction *III*′ detected under N_2_, suggesting that the triple-reduced species [**Me5]** ^**•2–**^ reacts with CO_2_. Addition of phenol (5%) increases the current at *III*′, while the reduction waves *I*′ and *II*′ shift anodically by *ca.* 80 mV. The changes in the voltammogram of **Me5** ^***Br***^ upon CO_2_ saturation suggest that CO_2_ interacts with the two-electron reduced species **[Me5]** ^**–**^, but only after the addition of the third electron does CO_2_ conversion take place.

In the case of the formazanate complex **4** ^***MeCN***^, only slight changes are observed under CO_2_ for the redox waves at mild potentials. However, at *ca.* −2.4 V a more prominent current increase is detected, with a peak current that is *ca.* 200 times higher than in the absence of CO_2_. When the CVs were recorded in the presence of phenol (5%), the current increase is observed at an even more negative potential of −2.8 V (Figure 10b). For both complexes, repeated CV cycling under CO_2_ results in diminished peak currents, indicating that degradation reactions also take place. Polishing the electrode recovered some of the initial activity, suggesting that precipitation and electrode fouling may occur.

Since the prominent current enhancements at *III*′ in the CV of both **Me5** ^***Br***^ and **4** ^***MeCN***^ under CO_2_ saturation suggested catalytic behavior, we carried out controlled potential electrolysis (CPE) experiments to analyze the products that are formed. While we did detect CO in the headspace of the H-cell after electrolysis at −2.8 V for 2 h in the presence of 5% phenol, its quantification by GC indicated poor faradaic efficiencies and very low turnover (**Me5** ^***Br***^ FE = 16%, TON = 0.93; **4** ^***MeCN***^ FE = 19%, TON = 0.66; **[4** ^***Br***^ **]** ^**–**^ FE = 21%, TON = 1.6). Moreover, an electrolysis experiment using **[4** ^***B*r**^ **]** ^**–**^ at −2.4 V with a ^13^CO_2_-saturated solution produced formate according to NMR analysis, but only in substoichiometric quantity (based on ^1^H NMR integration, see Figures S32 and S33). Thus, it seems that the increased current observed by CV is due to background reactivity (*i.e.*, solvent decomposition) at these negative potentials.

For the formazanate complexes **4** ^***MeCN***^ and [**4** ^***Br***^]^−^, the detection of a slight current increase at low overpotentials under CO_2_ saturation prompted us to further investigate this behavior by CPE. Electrolysis of a 1 mM solution of either **4** ^***MeCN***^ or [**4** ^***Br***^]^−^ past the potential of the reduction wave *I*′ in the presence of CO_2_ produced CO in substoichiometric quantities. It should be noted that in none of these experiments the CO produced is due to catalyst decomposition, since control experiments without CO_2_ did not generate GC-detectable quantities of CO. Moreover, the color of the solutions is markedly different when carried out under CO_2_ (yellow) or N_2_ (red), indicating that the Re complex reacts with CO_2_ at low overpotentials, albeit that no catalysis is observed (Figures S34 and S35).

We subsequently studied the speciation of formazanate Re­(I) complexes in the presence of CO_2_, both by IR spectroelectrochemistry and NMR spectroscopy (with ^13^CO_2_). We use **4** ^***MeCN***^ in the forthcoming discussion as a representative example as similar outcomes were found for **[4** ^**Br**^ **]** ^**–**^, which is consistent with the notion that both compounds generate the same bromide-free species upon reduction. The IR spectrum of the one-electron reduction product of **4** ^***MeCN***^ (generated at −1.5 V) in the presence of CO_2_ (*ca.* 0.14 M in acetonitrile) shows three metal–carbonyl stretching vibrations that are very similar to those without CO_2_, but in addition a new band at 1714 cm^–1^ is observed (Figure 11, red trace, labeled as *), which is indicative of a species that contains an organic carbonyl (C=O) group.

**Figure 11:**
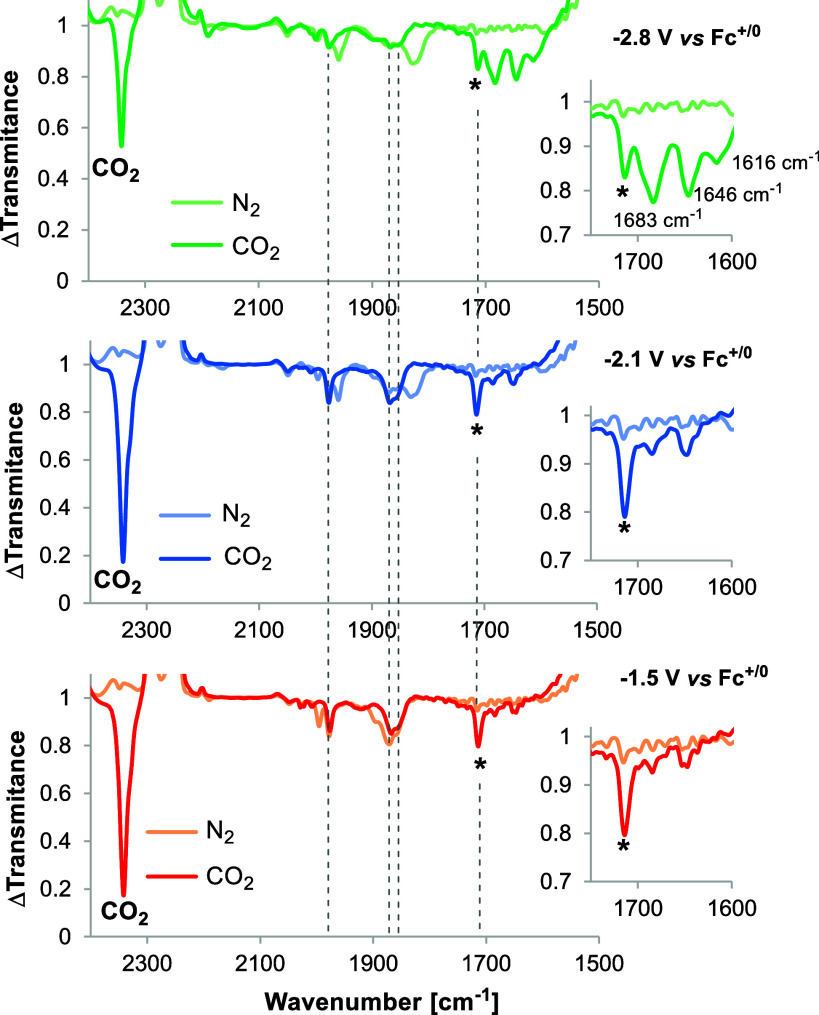
Infrared spectroelectrochemistry of 1.5 mM of **4** ^***MeCN***^ with 0.1 M of NBu_4_PF_6_ saturated with CO_2_ (dark trace); solution under N_2_ shown as light trace for comparison. (*) Corresponds to the band at 1714 cm^–1^.

Similarly, chemical reduction of **4** ^***MeCN***^ in CD_3_CN with 1 equiv Cp*_2_Co in an NMR tube under ^13^CO_2_ atmosphere revealed the appearance of new ^13^C NMR signals at 159.4 and 161.3 pm (Figure S36). A control experiment without reducing agent confirmed that the parent compound **4** ^***MeCN***^ is unreactive toward CO_2_. Addition of further reducing equivalents, either in the IR spectroelectrochemistry cell at −2.8 V or in an NMR tube with addition of 2 equiv Cp*_2_Co, resulted in more changes to the spectra. In the IR, low-energy ν­(CO) bands appear at 1683, 1648, and 1616 cm^–1^ whereas the ^13^C NMR shows a new major signal at 160.5 ppm. While we have not been able to isolate these compounds in pure form, the spectroscopic data indicate that the reduced forms of **4** ^***MeCN***^ react with CO_2_ resulting in products that have spectroscopic signatures consistent with carbonate (CO_3_^2–^/HCO_3_^–^)^61^ or carbamate^62^ species. Roesky and co-workers reported a magnesium formazanate complex that inserts CO_2_ into the Mg–N­(formazanate) bond to give a carbamate product with similar spectroscopic features.^63^ Our group has previously explored the reactivity of reduced boron and zinc formazanate complexes, wherein the nucleophilic character of the *N*-atoms in the ligand has been harnessed with different electrophiles (PhCH_2_Br, H^+^,^30^ lactide^64^). Accordingly, the experimental and computational lines of evidence discussed above indicate that upon reduction of **4** ^***MeCN***^, the nucleophilicity of the *N*-atom of the formazanate ligand in the radical anion **[4]** ^**•–**^ is larger than the nucleophilicity of the reduced metal ion resulting in an attack of the ligand onto CO_2_ and formation of a carbamate group (Figure 12).^65–67^ We computed the structure of the putative *N*-CO_2_ formazanate adduct **[4** ^***N*‑*CO***_**2**_^ **]** ^**•–**^, which has a tridentate NNO ligand that leads to two distinct binding modes. The geometries of the *facial* and *meridional* isomers were optimized using density functional theory calculations at the MN15L^54^/def2-tzvp^55^ level, which both converged to minima on the potential energy surface. The C=O stretching frequency of the carbamate unit in the two isomeric structures is calculated to be identical at 1731 cm^–1^, and agrees well with the empirically observed band at 1714 cm^–1^ (Figures S39 and S40). The difference in the DFT-computed Gibbs free energy between the isomers is substantial, with the *fac* isomer favored by Δ*G* = −18.5 kcal/mol. A comparison between the reaction product *fac*-**[4** ^***N*‑*CO***_**2**_^ **]** ^**•–**^ and the starting materials (**[4]** ^**•–**^ + CO_2_) indicates that binding of CO_2_ in this metal–ligand cooperative manner is indeed downhill (Δ*G* = −5.8 kcal/mol in the gas phase). The theoretical ν­(CO) stretching frequencies in *fac*-**[4** ^***N*‑*CO***_**2**_^ **]** ^**•–**^ are similar to the experimental ν­(CO) vibrations (DFT: 1987, 1904, 1896 cm^–1^, average = 1929 cm^–1^; Exp: 1976, 1867, 1857 cm^–1^, average = 1900 cm^–1^). Although further work is needed to fully confirm the identity of the CO_2_ adduct, examples of ligand-based reactivity of CO_2_ have been reported to be detrimental to catalysis^12,68,69^ and it seems that similar deleterious reactivity may occur here as well.

**Figure 12:**
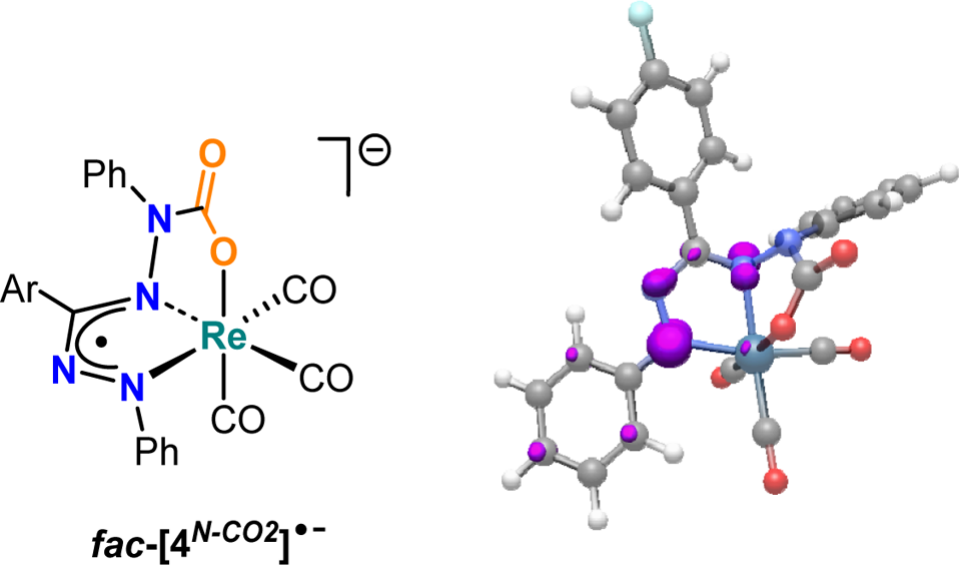
Structure of the putative CO_2_ adduct *fac*-**[4** ^***N‑CO***_**2**_^ **]** ^**•–**^ and its spin density plot (isovalue = 0.01).

## Conclusions

We described the synthesis, characterization, and reduction chemistry of Re­(I) carbonyl complexes comprising neutral (methyl)­formazan and anionic formazanate ligands. The spectroscopic and structural characterization of these species indicates a common “*open*” coordination mode of the ligands to form five-membered chelates with a pendant *N*-group, which contrasts the so far observed six-membered chelate rings found for anionic formazanate ligands. The (spectro)­electrochemical characterization of these species manifests the rich redox chemistry of formazan and formazanate ligands when bound to Re. We find that protonated (N–H) formazan Re­(I) complexes are unstable upon one-electron reduction, and evolve H_2_ via reductive NH bond cleavage forming their anionic formazanate analogues. On the other hand, the corresponding *N*-Me formazan complexes are stable and lead to sequential one-electron reduction chemistry (up to 3e^–^) as shown by spectroelectrochemistry. DFT calculations for both *N*-methylformazan and formazanate complexes support ligand-based 2e^–^ redox reactions in these compounds. The reactivity of reduced compounds toward CO_2_ was explored by cyclic voltammetry and spectroelectrochemistry studies. A comparison between data obtained with and without CO_2_ present in solution shows that CO_2_ has a significant effect on the electrochemistry of these compounds, but controlled potential electrolysis experiments indicated they are not active catalysts for CO_2_ electroreduction. The spectroelectrochemistry data under CO_2_ atmosphere provides evidence that, upon reduction, the pendant *N*-group in the ligand may be involved in CO_2_ capture to form a Re-bound carbamate fragment that is not further converted to the desired CO_2_ reduction products.

The presence of the redox-active formazanate ligand in Re­(I) tricarbonyl complexes not only enables milder reductions—compared to those reported for the archetypical Lehn’s catalyst ReCl­(CO)_3_(bpy) and derivatives thereof—but also switches the *locus* of reactivity to the ligand scaffold.

These results highlight the importance of judicious ligand design in homogeneous CO_2_ electroreduction catalysts: ligand-based “pooling” of redox equivalents may prove to be too much of a good thing when it opens up unproductive reaction pathways. Overall, a better understanding of the role that the redox-active ligand plays in multielectron reactions (*e.g.* as electron reservoir to facilitate multielectron substrate conversion vs a center for dead-end reactivity) is key for improved catalyst design and to expand the applicability of such ligands in novel chemical transformations.

## Experimental Section

### General Considerations

All manipulations were performed under N_2_ atmosphere using Schlenk/vacuum line and glovebox techniques. The solvents (Aldrich, anhydrous 99.8%) were passed over columns of Al_2_O_3_ (Fluka) and BASF R3-11-supported Cu oxygen scavenger. Complex **H4** ^***Br***^ was synthesized using the methodology previously reported by our group.^32^ ReBr­(CO)_5_ was prepared as previously reported^70^ from Re_2_(CO)_10_ (Aldrich, 98%) and Br_2_ (Aldrich, 98%). Bis­(triphenylphosphine)­iminium chloride [PPN]­[Cl] (Aldrich, 97%) tetraphenylphosphonium bromide [PPh_4_]­[Br] (Aldrich 97%), CH_3_I (Merck 99%), NEt_3_ (Aldrich 99.5%) and (1,4-diazabicyclo[2.2.2]­octane) DABCO (TCI 98%), ethyl *ortho*-formate (Aldrich 98%), phenylhydrazine (Aldrich 97%) and HBF_4_ (Aldrich 48 wt % in H_2_O) were used without further purification. **L5Me** was prepared following the procedure reported by Neugebauer^33^ from **L5H** and CH_3_I. THF-*d*_8_ was dried over Na/K and distilled via vacuum transfer. NMR spectra were measured on Mercury 400, Varian Inova 500, or Bruker 600 MHz spectrometers. Residual solvent signals were used as an internal reference for ^1^H and ^13^C spectra and reported in ppm relative to TMS (0 ppm). Full assignments were based on two-dimensional experiments (COSY, HSQC, HMBC) using standard pulse sequences. FT-IR spectra were collected in THF solution on a JASCO 4700 series FT-IR spectrometer in transmission mode using a liquid cell with CaF_2_ windows. UV–vis spectra were recorded in THF solution on an Avantes AvaSpec-2048 UV–vis spectrophotometer. X-ray diffraction data were collected at 100 K on a Bruker D8 Venture diffractometer with Mo Kα (λ = 0.71073 Å) radiation source. The crystal structure was refined using the SHELXL^71^ software. Non-hydrogen atoms were refined anisotropically (Table 4).

**Table 4:** Crystallographic Data for [Co­(Cp*)_**2**_ **]­[4** ^***Br***^ **]**, **5** ^***Py***^, **H5** ^***Br***^, **Me5** ^***Br***^ and **[5^*MeCN*^]­[PF_6_]**

	[Co(Cp*)_**2**_ **][4** ^***Br***^ **]**	**5** ^***Py***^	**H5** ^***Br***^	**Me5** ^***Br***^	**[5** ^***MeCN***^ **][PF**_**6**_ **]**
chemical formula	C_42_H_44_CoFBrN_4_O_3_Re	C_21_H_16_N_5_O_3_Re	C_16_H_12_BrN4O_3_Re	C_17_H_14_BrN_4_O_3_Re	C_19_H_17_F_6_N_5_O_3_PRe
*M*_r_	996.86	572.59	574.41	588.43	694.54
cryst syst	monoclinic	monoclinic	triclinic	orthorhombic	triclinic
color, habit	black, needle	blue, plate	dark-red, block	dark-red, needle	dark-red, needle
size (nm)	0.37 × 0.07 × 0.06	0.22 × 0.18 × 0.02	0.06 × 0.02 × 0.01	0.14 × 0.01 × 0.01	0.35 × 0.040 × 0.030
space group	*P*2_1_/*n* (no. 14)	*P*2_1_/*n* (no. 14)	*P*1̅ (no. 2)	*Pbnc* (no. 60)	*P*1̅ (no. 2)
*a* (Å)	15.0946(10)	8.4163(6)	7.3741(10)	30.805(3)	6.7615(4)
*b* (Å)	17.0051(14)	11.1815(9)	9.1941(12)	7.5141(3)	12.8316(7)
*c* (Å)	15.5791(12)	44.434(3)	13.765(3)	15.9819(10)	13.9441(10)
α (deg)	90	90	71.633(11)	90	77.321(2)
β (deg)	101.586(3)	93.023(3)	86.853(10)	90	79.800(3)
γ (deg)	90	90	81.765(14)	90	85.276(2)
*V* (Å^3^)	3917.4(5)	4175.8(5)	876.5(3)	3699.4(4)	1160.46(13)
*Z*	4	8	2	8	2
ρ_calc_ (g·cm^–3^)	1.690	1.821	2.176	2.113	1.988
radiation, λ (Å)	Mo Kα, 0.71073	Cu Kα, 1.54178	Cu Kα, 1.54178	Cu Kα, 1.54184	Cu Kα, 1.54178
μ(Mo Kα) (mm^–1^)	4.579	11.661	16.440	15.601	11.644
*F*(000)	1976	2208	540	2224	668
temp (K)	100	100(2)	295(2)	295(2)	170(2)
θ range (deg)	2.091–26.469	4.077–66.702	3.383–69.964	2.869–72.859	3.293–68.453
data collected (*h*, *k*, *l*)	–18:18, –21:21, –19:19	–9:10, –13:13, –52:52	–8:8, –11:11, –16:16	–38:37, –9:9, –19:19	–8:8, –15:15, –16:16
no. of reflns collected	85 881	50 837	15 403	74 921	33 779
no. of indep reflns	8052	7313	3267	3628	4166
obsd reflns *F*_o_ ≥ 2.0σ(*F*_o_)	6854	7267	2988	3530	4101
*R*(*F*) [obsd reflns] (%)	3.65	0.0409	2.87	1.93	0.051
*R*_w_(*F* ^2^) [all reflns] (%)	8.052	0.0990	7.15	4.83	0.149
GOF	1.321	1.245	1.049	1.137	1.194
weighting *a*, *b*	0.0000, 22.1095	0.0177, 44.7432	0.0000, 2.2659	0.0198, 3.4590	0.0543, 26.6174
params refined	516	581	230	236	318
min, max residual densities	–1.15, 1.08	–2.204, 1.632	–0.97, 1.03	–0.707, 0.691	–2.307, 2.655

Offline analysis of gaseous products was carried out either on a Shimadzu GC-2014 equipped with a TCD detector and on a ShinCarbon column or on a HP 5890 series II instrument with a TCD detector. For experiments at low overpotential values, the sample was passed through a Varian CP-PoraBOND Q (50 m × 0.53 m × 10 μm) and an Agilent Technologies HP-Molsieve (30 m × 0.53 mm × 50 μm) column. For more details about GC quantification see Supporting Information.

### Synthesis of 1,5-Diphenylformazan (**L5H**)

Diphenyl formazan was synthesized using a modified procedure from the one reported by Von Pechmann.^72^ Equimolar amounts of ethyl-orthoformate (6.7 mmol, 1.1 mL) and phenylhydrazine (13.4 mmol, 1.3 mL) under acidic conditions (30 drops of HBF_4_ solution) were dissolved in 15 mL of acetonitrile. The reaction was refluxed overnight, turning dark red. The solvent was evaporated to 1/3 of the original volume, and cold water was poured into the flask until complete precipitation. The solid was filtered and purified by column chromatography in silica using as eluent DCM. The red fraction was collected, and violet crystals were formed upon solvent evaporation. (751 mg, 50%) ^1^H NMR (CDCl_3_, 25 °C, 400 MHz) δ/ppm: 7.24 (t, ^3^ *J* = 8 Hz, 2H, Ph *p*-H), 7.43 (t, ^3^ *J* = 8 Hz, 4H, Ph *m*-H), 7.57 (d, ^3^ *J* = 8 Hz, 4H, Ph *o*-H), 7.87 (s, 1H, NNCNN, CH), 11.02 (s, 1H, NNCNN, NH).

### Synthesis of **[NHEt**_**3**_ **]­[*n*** ^***Br***^ **]** (*n* = 1–4)

The neutral formazan species **H*n*** ^***Br***^ (0.08 mmol) was dissolved in THF (5 mL) and an equimolar amount of NEt_3_ (0.08 mmol) was added, leading to an immediate color change from dark red to blue-greenish. The mixture was stirred at room temperature for 30 min. The solvent was removed under vacuo, leaving an oily dark residue. Upon trituration with 5 mL of pentane, the product precipitated as a black powder. The mixture was filtered out, and the solid was washed (2 × 5 mL) with pentane. Finally, the solid was dried under vacuum. **[NHEt**_**3**_ **]­[1** ^***Br***^ **]**. ^1^H NMR (THF-*d*_8_, 25 °C, 400 MHz) δ/ppm: 1.13 (t, ^3^ *J* = 8 Hz, 9H, NHEt_3_^+^, CH_3_), 2.96 (q, ^3^ *J* = 8 Hz, 6H, NHEt_3_^+^, CH_2_), 3.1 (s, 1H, NHEt_3_^+^, NH), 7.10 (m, 3H, Ph–CN *m*-H, Ph–CN *p*-H), 7.20 (m, 2H, Ph-N=N *p*-H, Ph–N–N *p*-H), 7.32 (m, 6H, Ph-N=N *o*-H, Ph–N–N *m*-H, Ph-N=N *m*-H), 7.85 (d, 2H, ^3^ *J* = 8 Hz, Ph–N–N *o*-H), 8.03 (d, 2H, ^3^ *J* = 8 Hz, Ph-NC *o*-H). IR (C_7_H_8_) ν­(CO)/cm^–1^: 2011(s), 1912(s), 1891(s). **[NHEt**_**3**_ **]­[2** ^***Br***^ **]**. ^1^H NMR (THF-*d*_8_, 25 °C, 400 MHz) δ/ppm: 1.10 (t, ^3^ *J* = 8 Hz, 9H, NHEt_3_^+^, CH_3_), 2.33 (s, 3H, *p*-tol CH_3_) 2.93 (q, ^3^ *J* = 8 Hz, 6H, NHEt_3_^+^, CH_2_), 4.11 (s, 1H, NHEt_3_^+^, NH), 7.10 (t, 1H, ^3^ *J* = 8 Hz, Ph–N–N *p*-H), 7.15 (m, 4H, *p*-tol *m*-H, Ph–N=N *o*-H), 7.20 (t, 1H, ^3^ *J* = 8 Hz, Ph–N=N *p*-H), 7.31 (m, 4H, Ph–N=N *m*-H, Ph–N–N *m*-H), 7.86 (d, 2H, ^3^ *J* = 8 Hz, Ph–N–N *o*-H), 7.95 (d, 2H, ^3^ *J* = 8 Hz, *p*-tol *o*-H). ^13^C­{^1^H} NMR (THF-*d*_8_, 25 °C, 151 MHz) δ/ppm: 8.04 (NHEt_3_^+^ CH_3_), 20.08 (*p*-tol CH_3_), 46.19 (NHEt_3_^+^ CH_2_), 121.64 (Ph–N–N *ipso*-C), 121.82 (Ph–N–N *o*-CH), 124.14 (Ph–N–N *p*-CH), 125.45 (Ph–N=N *p*-CH), 127.25 (Ph–N–N *m*-CH), 127.44 (*p*-tol *m*-CH), 127.67 (Ph–N=N *m*-CH), 128.91 (*p*-tol *o*-CH), 131.24 (Ph–N=N *ipso*-C), 136.05 (*p*-tol *p*-C), 156.82 (Ph–N–N *ipso*-C), 157.51 (imine-C), 189.97 (CO *trans* Br C), 193.25 (CO *trans* Ph–N=N C), 197.39 (CO *trans* Ph–N–N C). IR (C_7_H_8_) ν­(CO)/cm^–1^: 2011(s), 1912(s), 1891(s). **[NHEt**_**3**_ **]­[3** ^***Br***^ **]**. ^1^H NMR (THF-*d*_8_, 25 °C, 400 MHz) δ/ppm: 1.09 (t, ^3^ *J* = 8 Hz, 9H, NHEt_3_^+^, CH_3_), 2.92 (q, ^3^ *J* = 8 Hz, 6H, NHEt_3_^+^, CH_2_), 3.79 (s, 3H, *p*-MeO-Ph CH_3_), 4.0 (s, 1H, NHEt_3_^+^, NH), 6.93 (d, 2H, ^3^ *J* = 8 Hz, *p*-MeO-Ph *m*-CH), 7.18 (m, 4H, Ph–N=N *p*-H, Ph–N–N *p*-H, Ph–N=N *o*-H), 7.35 (m, 4H, Ph–N–N *m*-H, Ph–N=N *m*-H), 7.87 (d, 2H, ^3^ *J* = 8 Hz, Ph–N–N *o*-H), 7.98 (d, 2H, ^3^ *J* = 8 Hz, *p*-MeO-Ph *o*-H). ^13^C­{^1^H} NMR (THF-*d*_8_, 25 °C, 151 MHz) δ/ppm: 9.15 (NHEt_3_^+^ CH_3_), 47.30 (NHEt_3_^+^ CH_2_), 55.34 (*p*-MeO-Ph CH_3_), 113.73 (*p*-MeO-Ph *m*-CH), 122.42 (Ph–N=N *p*-CH), 123.30 (Ph–N–N *o*-CH), 126.52 (Ph–N=N *o*-CH), 128.63 (Ph–N–N *m*-CH), 129.02 (Ph–N=N *m*-CH), 131.40 (*p*-MeO-Ph *o*-CH), 158.07 (Ph–N–N *ipso*-C), 160.50 (imine-C), 190.18 (CO *trans* Br C), 194.24 (CO *trans* Ph–N=N C), 197.55 (CO *trans* Ph–N–N C). IR (C_7_H_8_) ν­(CO)/cm^–1^: 2011(s), 1912(s), 1891(s). **[NHEt**_**3**_ **]­[4** ^***Br***^ **]**. Similar spectroscopic data to those reported with other counterions below.

### Synthesis of **[PPN]­[4** ^***Br***^ **]**

A Schlenk flask was charged with 0.0490 g (0.073 mmol) of **H4** ^***Br***^ and 0.0422 g (0.073 mmol) of [PPN]­Cl. The solids were dissolved in 5 mL of THF, and 0.01 mL (0.073 mmol) of NEt_3_ were added, observing an immediate color change from dark purple to green-blue. The mixture was kept stirring for 2 h, filtered, and the filtrate evaporated to dryness. A dark precipitate was formed after the addition of 5 mL of pentane. The solid was rinsed twice with pentane (5 mL) and dried under vacuum. (41.3 mg, 84%). **[PPh**_**4**_ **]­[4** ^***Br***^ **]**. A similar procedure was followed. The reaction was carried out with equimolar amounts of **H4** ^***Br***^ (0.05 g, 0.075 mmol), NEt_3_ (0.01 mL, 0.075 mmol), and [PPh_4_]­[Br] (0.0323 g, 0.075 mmol). (29.7 mg, 40%). [Co­(Cp*)_**2**_ **]­[4** ^***Br***^ **]**. In a 20 mL vial, 0.0580 g (0.087 mmol) of **H4** ^**Br**^ and 0.0286 g (0.087 mmol) of Co­(Cp*)_2_ were added and mixed with 5 mL of THF. The reaction was stirred for 24 h, and gradually, the solution turned dark green-blue: the characteristic color of the formazanate species. The solution was filtered, and by slow evaporation, a crystalline material was obtained. ^1^H NMR (THF-*d*_8_, 25 °C, 600 MHz) δ/ppm: 1.67 (s, 30H, Cp* CH_3_), 6.95–6.99 (m, 3H, Ph–N–N *p*-H, *p*-FPh *m*-H), 7.13–7.17 (m, 3H, Ph–N=N *o*-H, Ph–N=N *p*-H), 7.22 (t, 2H, ^3^ *J* = 8 Hz, Ph–N–N *m*-H), 7.28 (t, 2H, ^3^ *J* = 8 Hz, Ph–N=N *m*-H), 7.83 (d, 2H, ^3^ *J* = 8 Hz, Ph–N–N *o*-H), 8.12 (dd, 2H, ^3^ *J*_H–H_ = 8 Hz, ^4^ *J*_H–F_ = 6 Hz, *p*-FPh *o*-H). ^19^F NMR (THF-*d*_8_, 25 °C, 565 MHz) δ/ppm: −115.80 (m, *p*-FPh F). ^13^C­{^1^H} NMR (CDCl_3_, 25 °C, 151 MHz) δ/ppm: 6.73 (CH_3_ Cp*), 93.73 (*ipso*-C Cp*), 112.81 (d, ^2^ *J*_C–F_ = 21 Hz, *p*-FPh *m*-CH), 121.94 (Ph–N=N *o*-CH), 122.97 (Ph–N–N *p*-CH), 124.89 (Ph–N=N *p*-CH), 126.91 (Ph–N–N *m*-CH), 127.20 (Ph–N=N *m*-CH), 130.82 (d, ^3^ *J*_C–F_ = 7 Hz, *p*-FPh *o*-CH), 131.25 (d, ^4^ *J*_C–F_ = 3 Hz, *p*-FPh *ipso*-C), 155.15 (imine-C), 156.94 (Ph–N–N *ipso*-C), 157.39 (Ph–N=N *ipso*-C), 160.93 (*J*_C–F_ = 245 Hz, *p*-FPh *p*-C), 190.49 (CO *trans* Br C), 193.64 (CO *trans* Ph–N=N C), 197.96 (CO *trans* Ph–N–N C). FT-IR (THF) ν­(CO)/cm^–1^: 2007(s), 1912(s), 1879(s). Anal. Calcd for (C_42_H_44_CoFBrN_4_O_3_Re): C 50.6, H 4.45, N 5.62; found C 50.38, H 4.50, N 5.58.

### Synthesis of **4** ^***MeCN***^

In 15 mL of acetonitrile were dissolved 0.3036 g (0.45 mmol) of **H4** ^***Br***^ with 1.2 equiv (0.1400 g) of AgPF_6_. The mixture was refluxed for 3 h in the darkness and then passed over Celite. Assuming full conversion to the resulting brown solution, 0.0509 g (0.45 mmol) of DABCO were added, observing immediately a blue intense color. After stirring for 30 min, the mixture was evaporated to dryness, and then redissolved in diethyl ether. This solution was filtered over neutral alumina, and the volatiles were removed, yielding a dark solid (131 mg, 46%). ^1^H NMR (acetonitrile-*d*_3_, 25 °C, 600 MHz) δ/ppm: 1.76 (s, 3H, NCCH_3_ CH_3_), 6.86 (d, 2H, ^3^ *J*_H–H_ = 8 Hz Ph–N=N *o*-H), 6.96 (t, 2H, ^3^ *J*_H–F_, ^3^ *J*_H–H_ = 8 Hz, *p*-FPh *m*-H), 6.99 (t, 1H,^3^ *J*_H–H_ = 8 Hz, Ph–N–N *p*-H), 7.22 (m, 5H, Ph–N–N *m*-H, Ph–N=N *m*-H, Ph–N=N *p*-H), 7.53 (d, 2H, ^3^ *J* = 8 Hz, Ph–N–N *o*-H), 7.72 (dd, 2H, ^3^ *J*_H–H_ = 8 Hz, ^4^ *J*_H–F_ = 6 Hz, *p*-FPh *o*-H). ^19^F NMR (acetonitrile-*d*_3_, 25 °C, 565 MHz) δ/ppm: −116.1 (m, *p*-FPh F). ^13^C­{^1^H} NMR (CDCl_3_, 25 °C, 151 MHz) δ/ppm: 0.71 (NCCH_3_ CH_3_), 117.1 (NCCH_3_ CN), 114.81 (d, ^2^ *J*_C–F_ = 21 Hz, *p*-FPh *m*-CH), 121.95 (Ph–N–N *o*-CH), 122.12 (Ph–N=N *o*-CH), 125.61 (Ph–N–N *p*-CH), 128.08 (Ph–N=N *p*-CH), 128.69 (Ph–N=N *m*-CH), 129.24 (Ph–N–N *m*-CH), 130.29 (d, ^4^ *J*_C–F_ = 5 Hz, *p*-FPh *ipso*-C), 132.02 (d, ^3^ *J*_C–F_ = 7 Hz, *p*-FPh *o*-CH), 156.63 (Ph–N–N *ipso*-C), 157.27 (Ph–N=N *ipso*-C), 157.72 (imine-C), 162.31 (*J*_C–F_ = 245 Hz, *p*-FPh *p*-C), 191.19 (CO *trans* Br C), 192.36 (CO *trans* Ph–N=N C), 195.04 (CO *trans* Ph–N–N C). IR (THF) ν­(CO)/cm^–1^: 2024(s), 1922­(broad). HRMS (ESI+) (*m*/*z*): Calcd for [MH]^+^ = 631.102958. Found = 631.09616. [M]^+^ = 630.095133. Found = 630.09281. [M – CO]^+^ = 602.100218. Found = 602.09800. [M – 3CO]^+^ = 547.118213. Found = 546.09710.

### Synthesis of **H5** ^***Br***^

In a 50 mL two-necked round-bottom flask were added 0.1018 g (0.25 mmol) of ReBr­(CO)_5_ and 0.05621 g (0.25 mmol) of **L5H**. The mixture was dissolved in 20 mL of toluene, rendering an orange-red solution that turned dark brown when the reaction proceeded. The solution was heated up to reflux for 45 min, and the volatiles were evaporated under vacuum to dryness, yielding a black fine powder. The solid was rinsed with hexane (3 × 5 mL). (130 mg, 91%) ^1^H NMR (CDCl_3_, 25 °C, 600 MHz) δ/ppm: 7.46 (d, 2H, ^3^ *J* = 8 Hz, Ph–NH *o*-H), 7.49 (t, 1H, ^3^ *J* = 8 Hz, Ph–N=N *p*-H), 7.54 (t, 2H, ^3^ *J* = 8 Hz, Ph–N=N *m*-H), 7.57 (m, 3H, ^3^ *J* = 8 Hz, Ph–NH *m*-H, *p*-H), 7.85 (d, 2H, ^3^ *J* = 8 Hz, Ph–N=N *o*-H), 8.72 (s, 1H, NH), 9.1 (s, 1H, NCN H). ^13^C­{^1^H} NMR (CDCl_3_, 25 °C, 150 MHz) δ/ppm: 123.89 (Ph–N=N *o*-CH), 125.19 (Ph–NH *o*-CH), 129.34 (Ph–N=N *m*-CH), 129.39 (Ph–N=N *p*-CH), 130.34 (Ph–NH *m*-CH), 131.89 (Ph–NH *p*-CH), 138.52 (Ph–NH *ipso*-C), 150.52 (imine C), 156.54 (Ph–N=N *ipso*-C), 180.82 (CO *trans* Br C), 193.07 (CO *trans* Ph–NH–N C), 194.33 (CO *trans* Ph–N=N C). FT-IR (THF) ν­(CO)/cm^–1^: 2031(s), 1953(s) 1919(s). HRMS (ESI−) (*m*/*z*): Calcd for [MH]^−^ = 574.95461. Found = 574.95544. [M]^−^ = 573.96001. Found = 573.96034. [M – 3CO]^−^ = 489.980297. Found = 489.95629.

### Synthesis of **Me5** ^***Br***^

Equimolar amounts of ReBr­(CO)_5_ (0.1173 g, 0.29 mmol) and **L5Me** (0.0688 g, 0.29 mmol) were dissolved in 20 mL of toluene. The yellow-orange solution was refluxed for 45 min. The resulting dark-brown mixture was cooled down to room temperature, and the solvent evaporated to dryness, yielding a dark crystalline material. The solid was rinsed with hexane (3 × 5 mL). Crystals were obtained by redissolving the solid in CH_2_Cl_2_ and subsequent slow solvent evaporation. (152.6 mg, 90%) ^1^H NMR (CDCl_3_, 25 °C, 600 MHz) δ/ppm: 3.93 (s, 3H, NMe CH_3_), 7.41 (d, 2H, ^3^ *J* = 8 Hz, Ph–NMe *o*-H), 7.54 (m, 6H, Ph–N=N *m*-H, *p*-H; Ph–NMe *m*-H, *p*-H), 7.84 (d, 2H, ^3^ *J* = 8 Hz, Ph–N=N *o*-H), 8.96 (s, 1H, NCN H). ^13^C­{^1^H} NMR (CDCl_3_, 25 °C, 150 MHz) δ/ppm: 45.78 (Ph–NMe CH_3_), 123.74 (Ph–N=N *o*-CH), 125.43 (Ph–NMe *o*-CH), 129.22 (Ph–N=N *m*-CH), 129.56 (Ph–N=N *p*-CH), 129.71 (Ph–NMe *m*-CH), 131.26 (Ph–NMe *p*-CH), 146.41 (Ph–NMe *ipso*-C), 149.87 (imine C), 156.84 (Ph–N=N *ipso*-C), 184.54 (CO *trans* Br C), 191.96 (CO *trans* Ph–NH–N C), 193.15 (CO *trans* Ph–N=N C). FT-IR (THF) ν­(CO)/cm^–1^: 2031(s), 1952(s), 1915(s). HRMS (ESI−) (*m*/*z*): Calcd for [M]^−^ = 587.98013. Found = 587.98046. [M – 3CO]^+^ = 503.99547. Found = 503.98046.

### Synthesis of **[Me5** ^***MeCN***^ **]­[PF**_**6**_ **]**

In a round-bottom flask, 0.0995 g (0.17 mmol) of **Me5** ^***Br***^ and 1.2 equiv of AgPF_6_ (0.0561 g, 0.22 mmol) were dissolved in 5 mL of MeCN. The reaction was performed in the darkness and heated to reflux for 4 h. Then, the mixture was cooled down and filtered through Celite. The solid was recrystallized by diffusion (DCM: pentane), obtaining red needles (73.1 mg, 63%). ^1^H NMR (THF-*d*_8_, 25 °C, 400 MHz) δ/ppm: 2.44 (s, 3H, MeCN CH_3_), 4.11 (s, 3H, NMe, CH_3_), 7.56–7.58­(m, 8H, Ph–NMe *o*-H, Ph–N=N *m*-H, *p*-H; Ph–NMe *m*-H, *p*-H), 7.89 (m, 2H, Ph–N=N *o*-CH), 9.43 (s, 1H, NCN H). FT-IR (THF) ν­(CO)/cm^–1^: 2048(s), 1968(s) 1952(s). HRMS (ESI+) (*m*/*z*): Calcd for [MH]^+^ = 551.09170. Found: 551.09229. [M]^+^ = 550.08834. Found: 550.23822.

### Synthesis of **5** ^***Py***^

**[Me5** ^***MeCN***^ **]­[PF**_**6**_ **]** (81.5 mg, 0.117 mmol, 1.0 equiv) and pyridine (75 μL, 0.93 mmol, 8.4 equiv) were dissolved in 8 mL of THF. The mixture was refluxed for 7 h, and the solvent was evaporated to dryness. The crude product was purified via column chromatography using DCM as eluent. The obtained dark blue material was recrystallized from pentane (18.1 mg, 21%). ^1^H NMR (THF-*d*_8_, 25 °C, 600 MHz) δ/ppm: 7.13 (t, 1H, ^3^ *J* = 7 Hz, Ph–N–N *p-C*H), 7.17 (d, 2H, ^3^ *J* = 8 Hz, Py *o*-CH), 7.40 (m, 5H, Py *m*-CH, Ph–N=N *m*-CH, Py *p*-CH), 7.47 (t, 2H, ^3^ *J* = 8 Hz, Ph–N–N *m*-CH), 7.78 (d, 2H, ^3^ *J* = 8 Hz, Ph–N–N *o*-CH), 7.92 (dt, 1H, ^3^ *J* = 8 Hz, ^1^ *J* = 1 Hz, Ph–N=N *p*-CH), 8.05 (d, 2H, ^3^ *J* = 6 Hz, Ph–N=N *o*-CH), 8.22 (s, 1H, NCN, H). ^13^C­{^1^H} NMR (THF-*d*_8_, 25 °C, 150 MHz) δ/ppm: 122.15 (*o*-CH, Ph–N–N), 123.28 (Py *o*-CH), 125.63 (Ph–N–N *p*-C), 127.23 (Ph–N–N *m*-CH), 129.13 (Py *p*-CH), 129.45 (Py *m*-CH), 129.86 (Ph–N=N, *m*-CH), 140.28 (Ph–N=N, *p*-CH), 150.63 (CH NCN), 153.78 (Ph–N=N *o*-C), 156.62 (Ph–N–N *ipso*-C), 157.47 (Ph–N=N *ipso*-C), 194.19 (CO *trans* Py), 194.48 (CO *trans* Ph–N–N), 197.97 (CO *trans* Ph–N=N). FT-IR (THF) ν­(CO)/cm^–1^: 2021, and 1920 cm^–1^. HRMS (ESI+) (*m*/*z*): Calcd for [M + H]^+^ = 574.08387. Found = 574.08826. Calcd for [M]^+^ = 573.08387. Found = 574.08826. [M – 2CO]^+^ = 518.09069. Found = 518.09882.

## Supplementary Material



## Notes

1D (^1^H, ^13^C­{^1^H}) and 2D (^1^H EXSY) NMR experiments, additional spectroscopy measurements (IR and UV–vis), electrochemical measurements (CV, CPE) and computational data (geometry optimizations, TDDFT calculations, and NTO analysis) (PDF)
