# Supplementary material for: Synthesis and Electrochemistry of Formazan(ate) Re(I) Complexes: Ligand-Based Reactivity toward CO2
Source: Inorg Chem. 2025 Oct 17;64(43):21603–19. doi: 10.1021/acs.inorgchem.5c03626 (PMC12587401; doi:10.1021/acs.inorgchem.5c03626)
Supplement: Supplementary file 1 [file ic5c03626_si_001.pdf]

Supporting information

Synthesis and electrochemistry of formazan(ate) Re(I) complexes: ligand-based reactivity toward CO_2

Liliana Capulín Flores,[a,b]* Sander J. Mondria,[a] Kai-Thorben Kuessner,[c] Philipp Rohatschek,[c] Inke Siewert,[c] Noé Zúñiga-Villarreal,[b] and Edwin Otten[a]

AUTHOR ADDRESSES

[a] Stratingh Institute for Chemistry, University of Groningen, Nijenborgh 3, 9747 AG Groningen, The Netherlands.

[b] Instituto de Química, Universidad Nacional Autónoma de México, Ciudad Universitaria, Circuito Exterior, 04510 México, D.F., México.

[c]Georg-August-Universität Göttingen, Institut für Anorganische Chemie, Tammannstr. 4, 37077 Göttingen, Germany.

*Corresponding Author: E-mail(L.C.F.): l.capulin.flores@rug.nl

Table of contents

	Page
Characterization	S3
Photophysical studies	S19
Electrochemical studies	S21
Spectroelectrochemistry	S23
Chemical reductions	S26
Electrochemistry under CO_2	S27
Chemical reductions in the presence of $^{13}CO_2$	S31
DFT	S32
TDDFT	S36
References	S38

Characterization

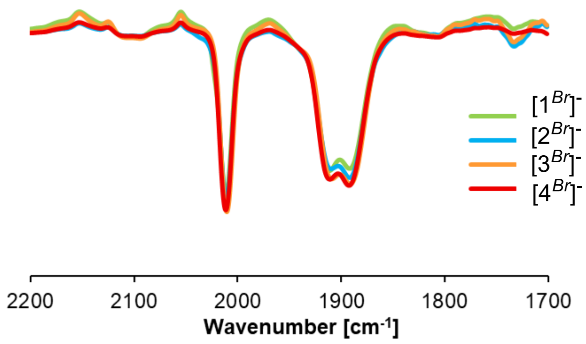

Figure S1. FT-IR spectra in toluene solution of formazanate complexes [$NHEt_3$] [1^{Br}]$^-$ - [4^{Br}]$^-$, R = H (**1**), Me (**2**), MeO (**3**) and F(**4**) in the presence of excess of NEt_3.

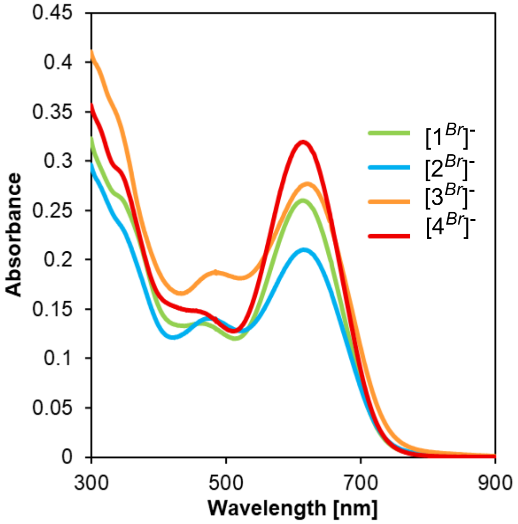

Figure S2. UV-vis spectra in toluene solution of formazanate complexes [[$NHEt_3$] [1^{Br}]$^-$ - [4^{Br}]$^-$, R = H (**1**), Me (**2**), MeO (**3**) and F(**4**) in the presence of excess of NEt_3.

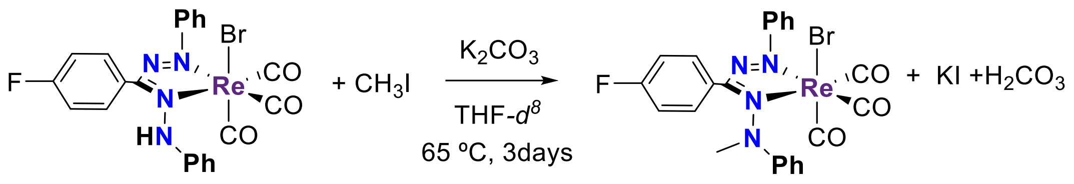

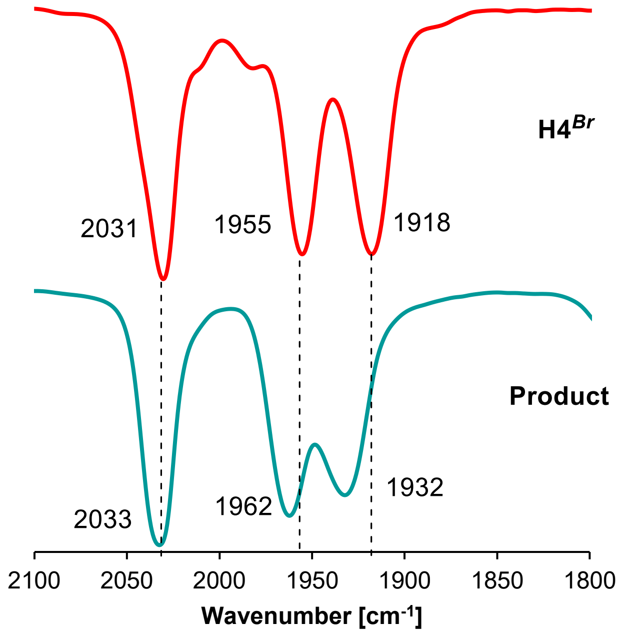

Figure S3. We also attempted direct alkylation on H4^{Br} with MeI and K_2CO_3. While the product was obtained, the reaction was difficult to reproduce. Here, the FT-IR spectra in THF-d_8 solution of the crude and starting material are shown.

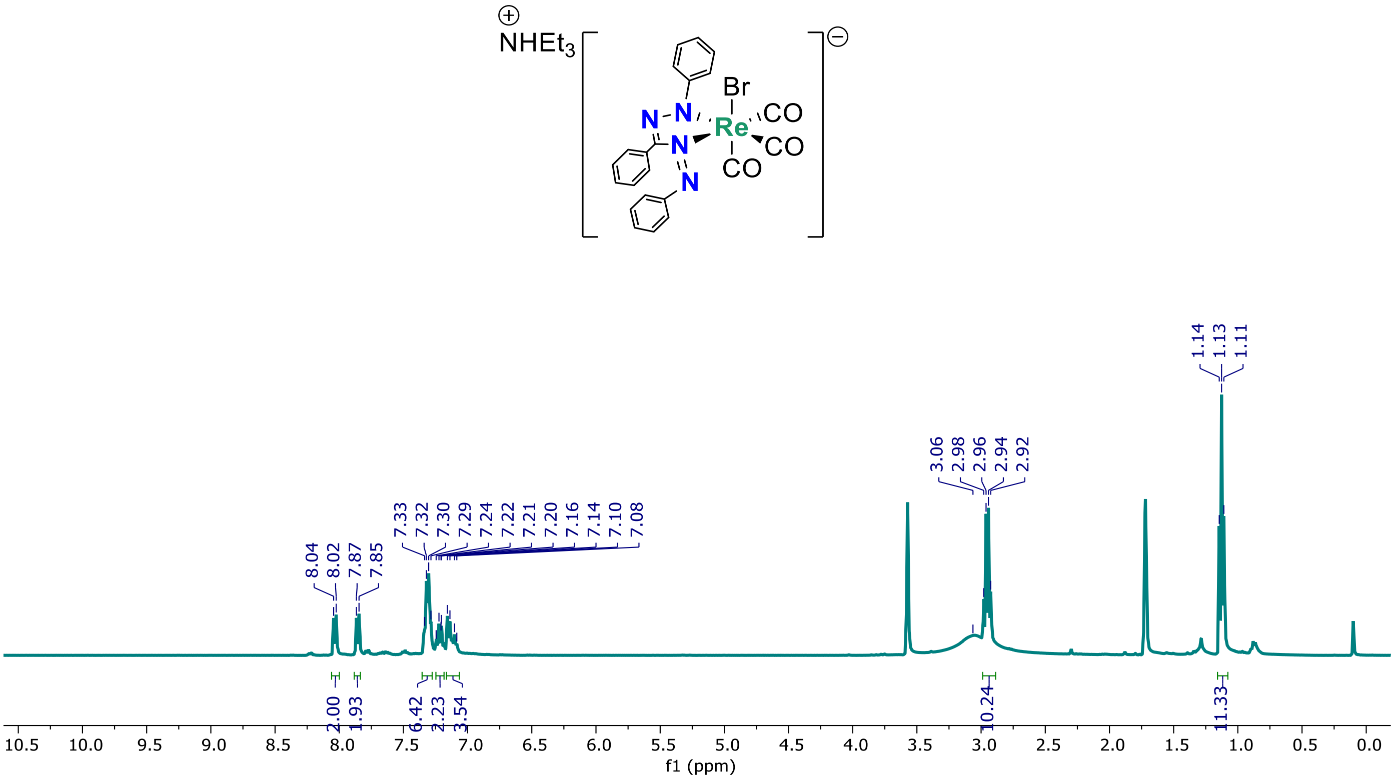

Figure S4. ^{1}H NMR spectrum of [NHEt$_3$][1^{Br}] in THF-d_8 at rt.

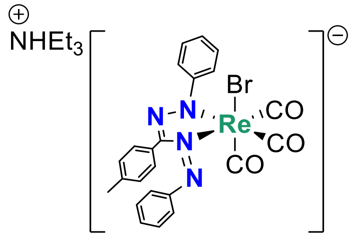

a)

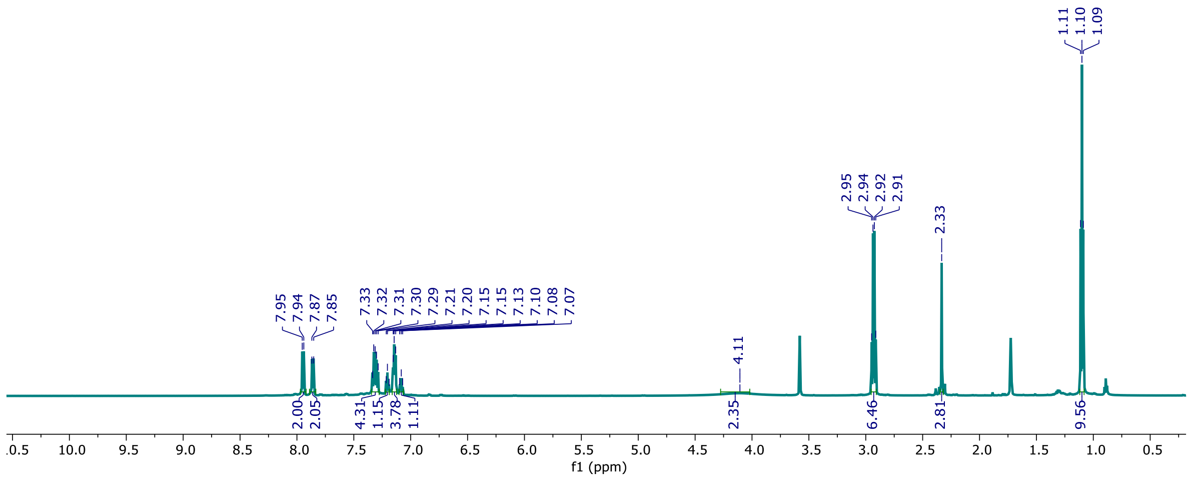

b)

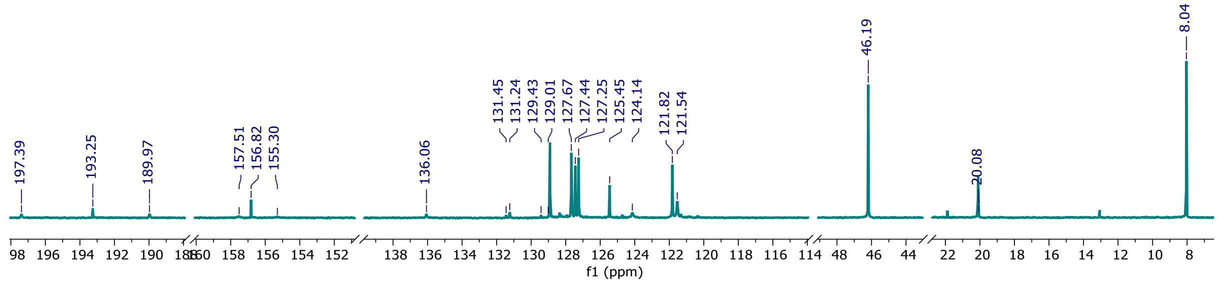

Figure S5. a)^{1}H and b) $^{13}C\{^{1}H\}$ NMR spectra of [NHEt$_3$][2^{Br}] in THF-d_8 at rt.

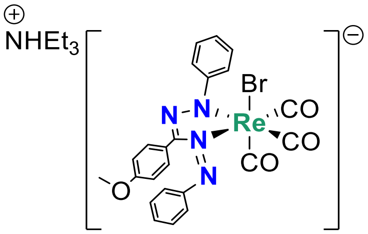

a)

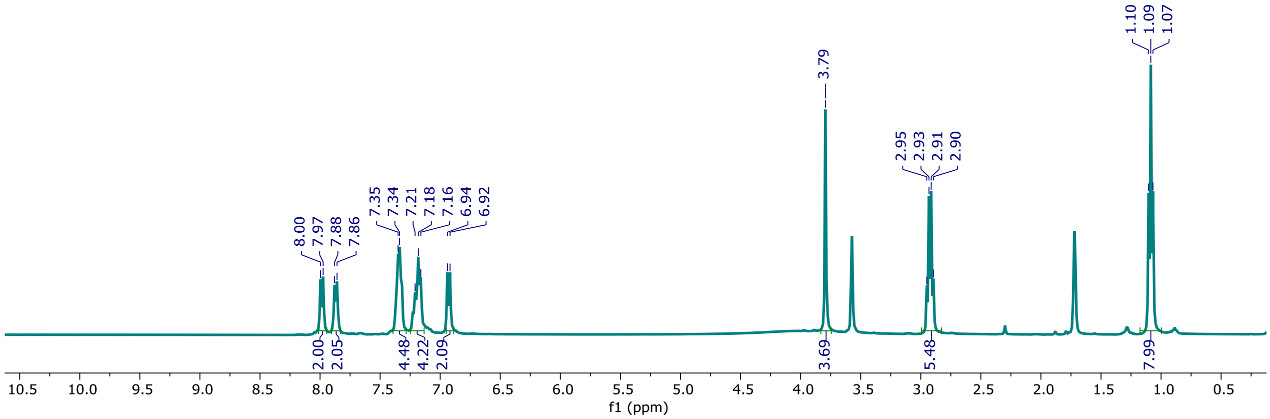

b)

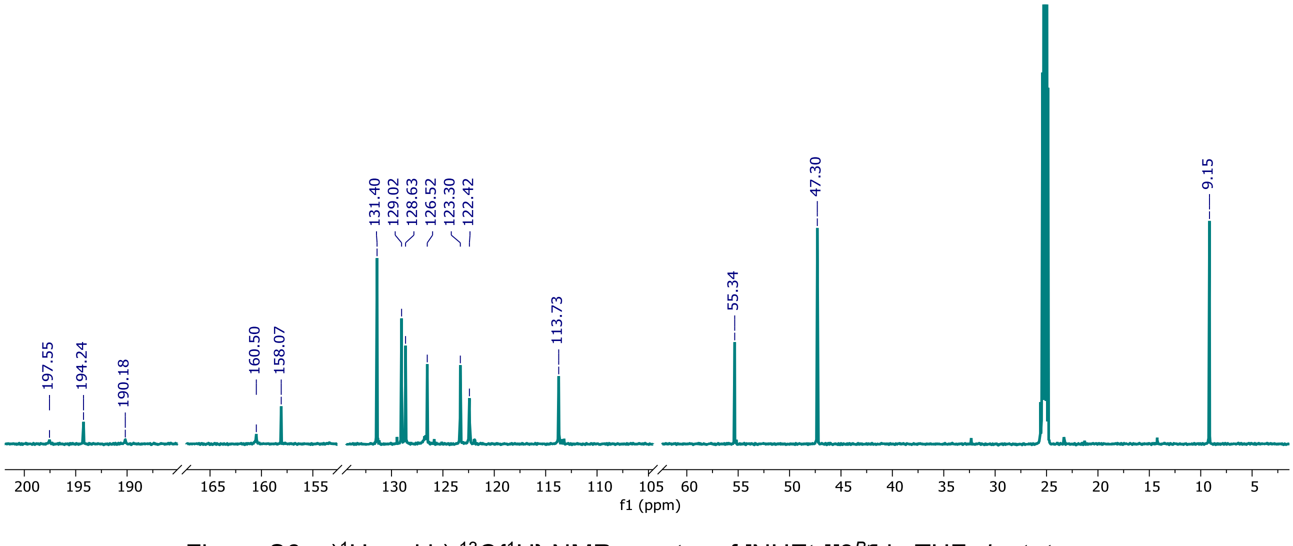

Figure S6. a)^{1}H and b) ^{13}C{^{1}H} NMR spectra of [NHEt$_3$][3^{Br}] in THF-d_8 at rt.

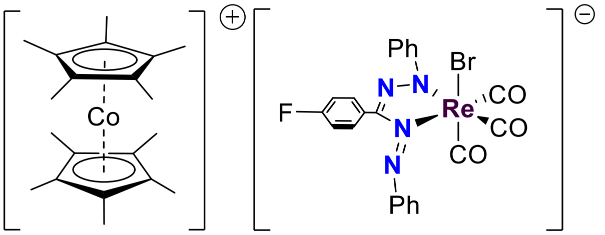

a)

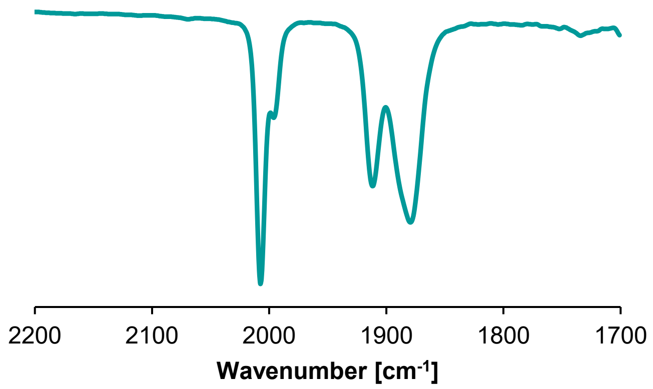

b)

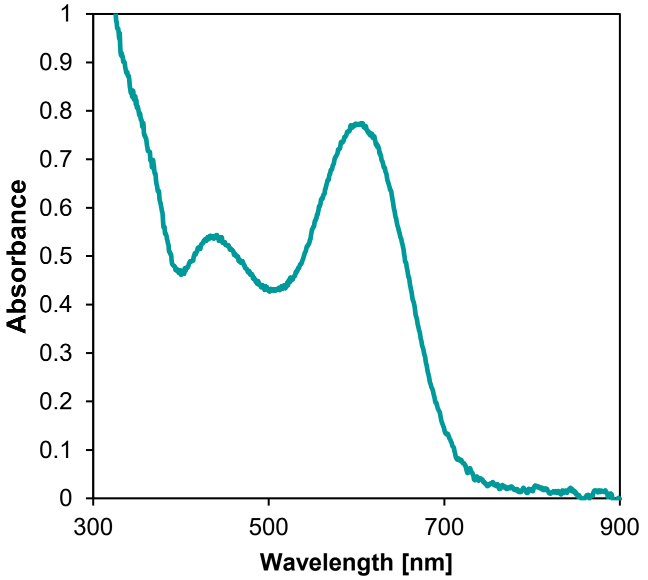

Figure S7. a) FT-IR and b) UV-vis spectra of $[Co(Cp^*)_2][4^{Br}]$ in THF at rt.

a)

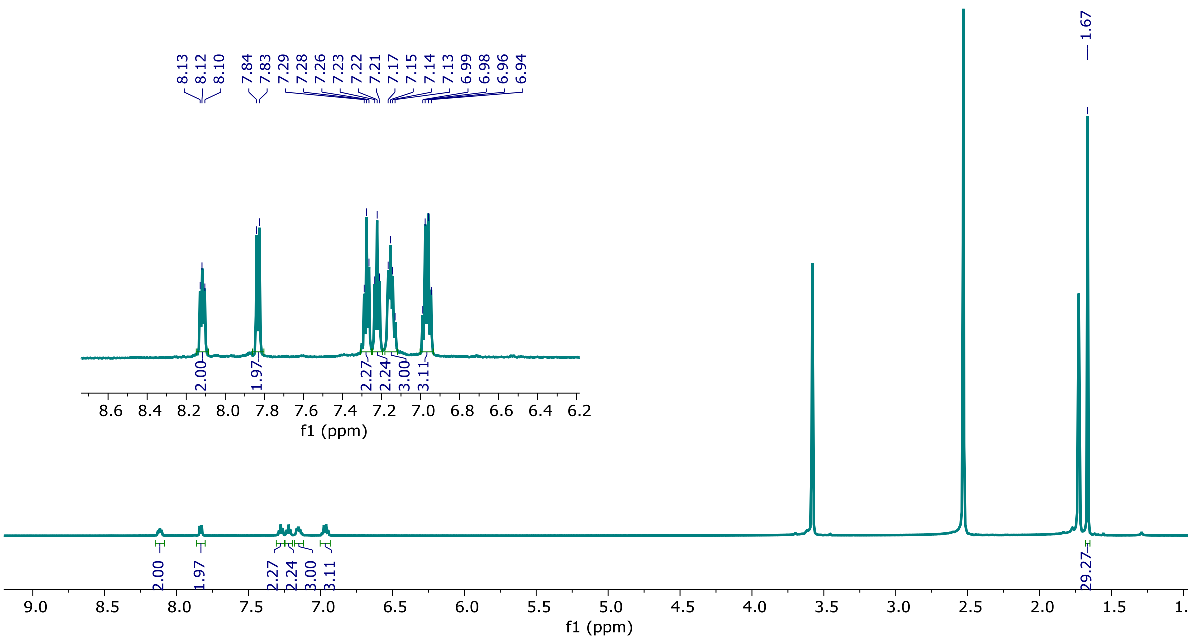

b)

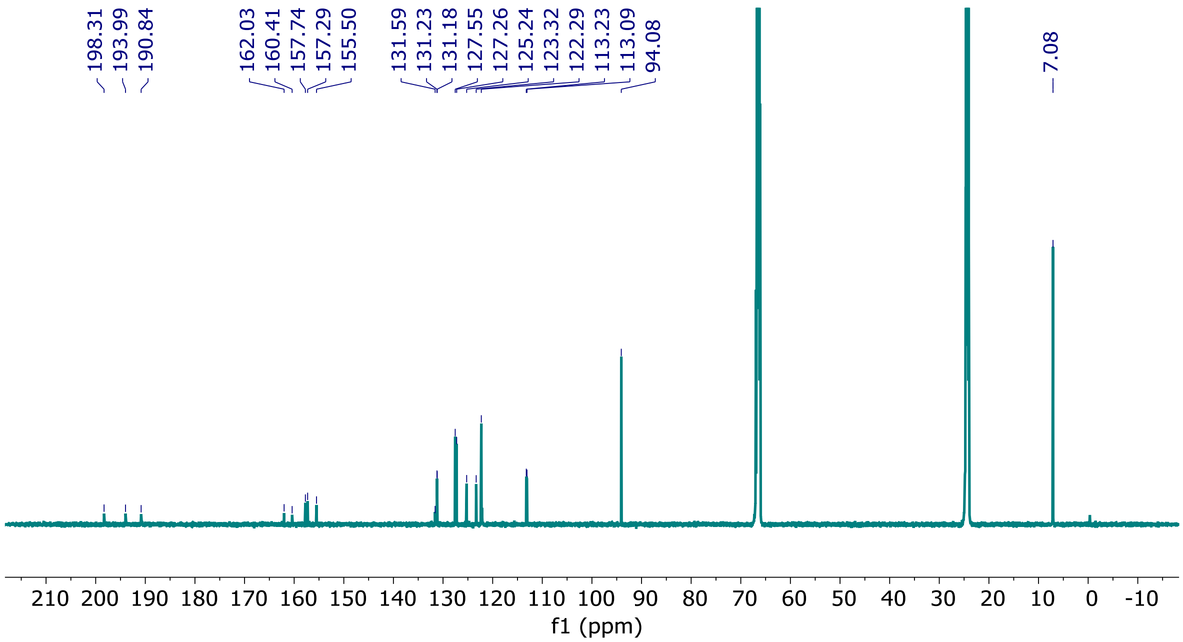

Figure S8. a)^{1}H and b) ^{13}C{^{1}H} NMR spectra of [Co(Cp*)$_2$][4^{Br}] in THF-d_8 at rt.

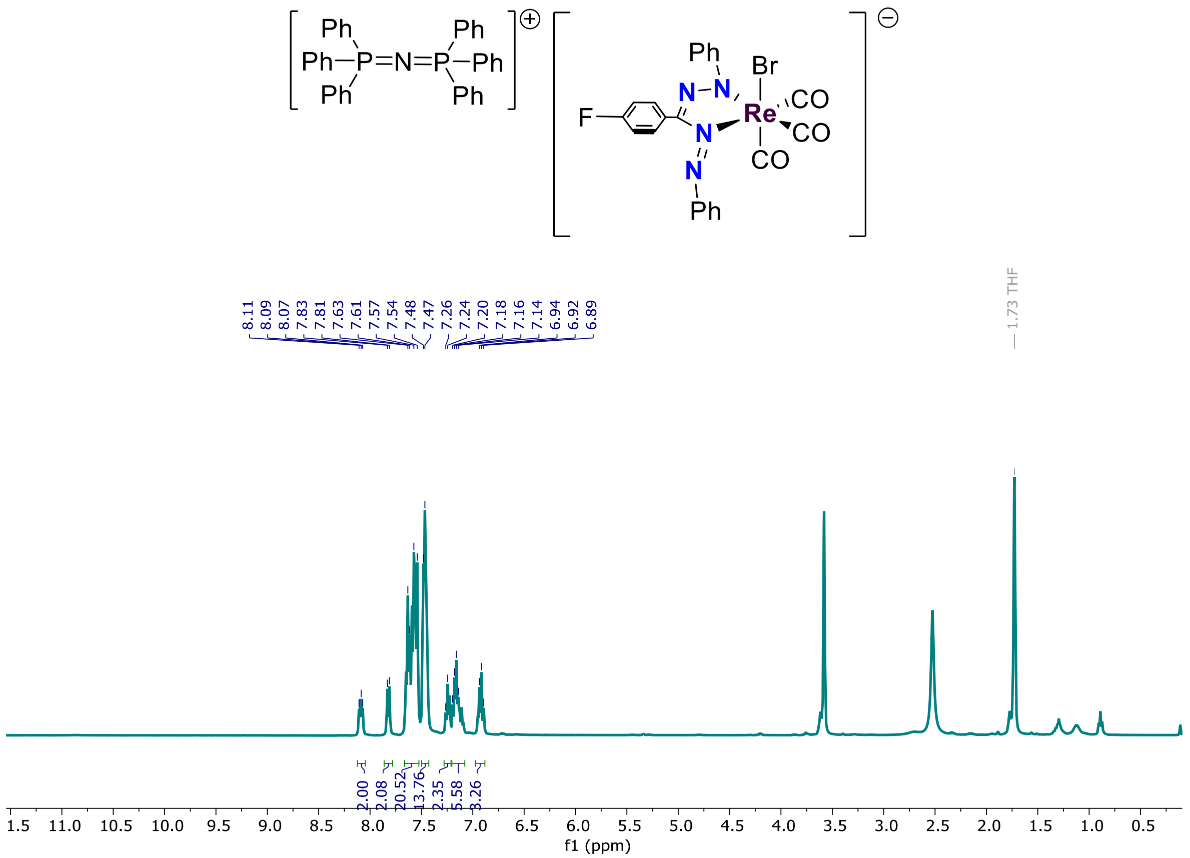

Figure S9. ^{1}H NMR spectrum of [PPN][4^{Br}] in THF-d_8 at rt.

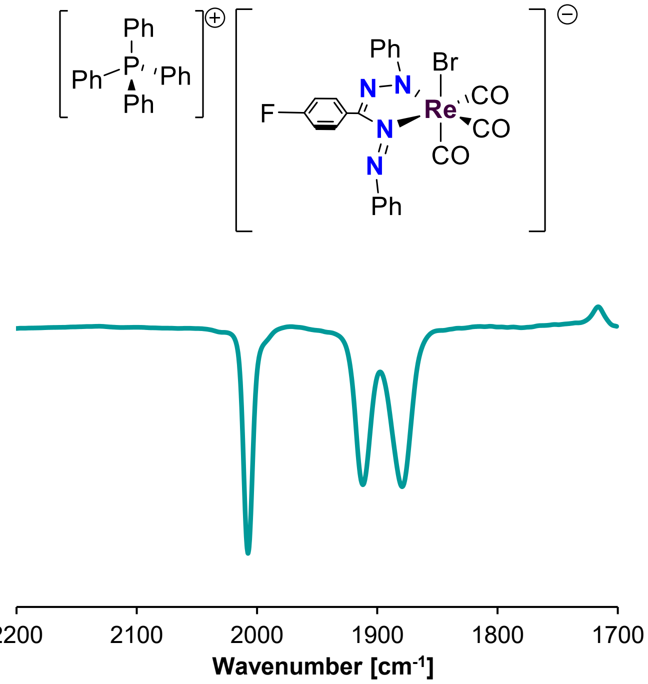

Figure S10. FT-IR spectrum of $[PPh_4][4^{Br}]$ in THF.

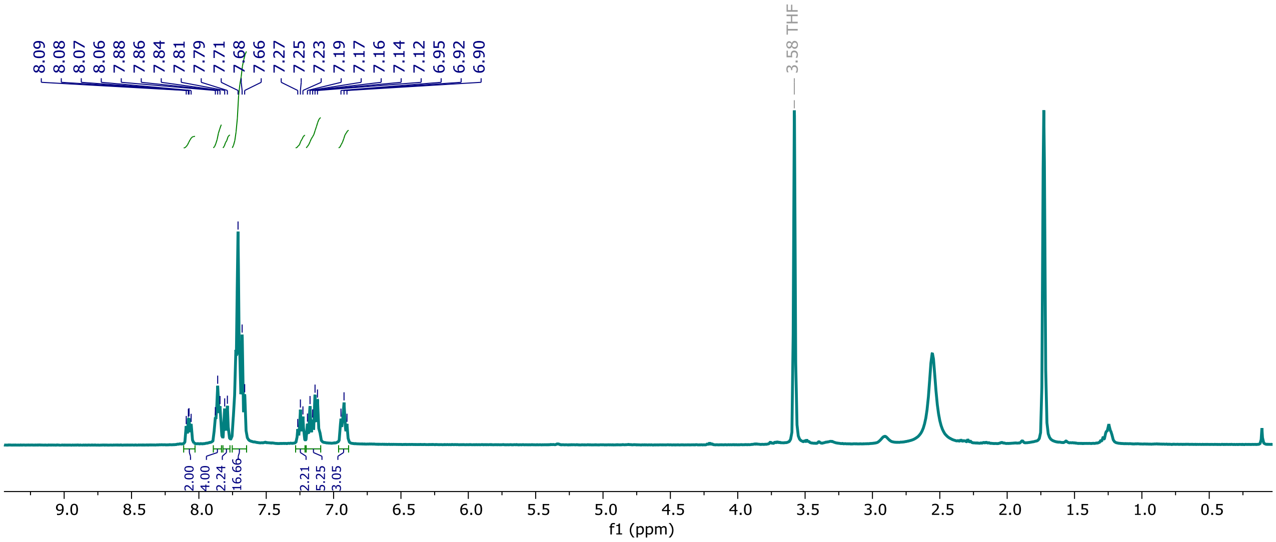

Figure S11. ^1H NMR spectrum of $[PPh_4][4^{Br}]$ in THF-d_8 at rt.

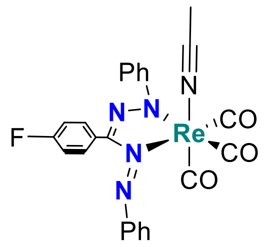

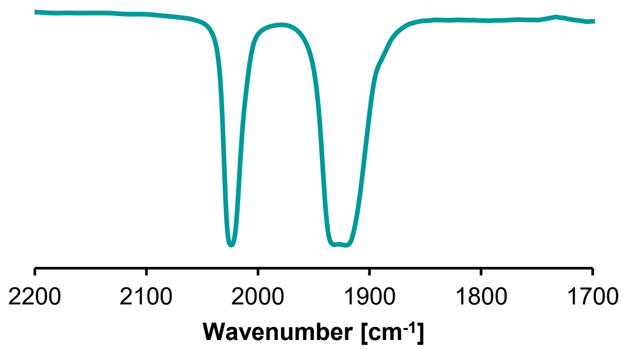

Figure S12. FT-IR of 4^{MeCN} in THF.

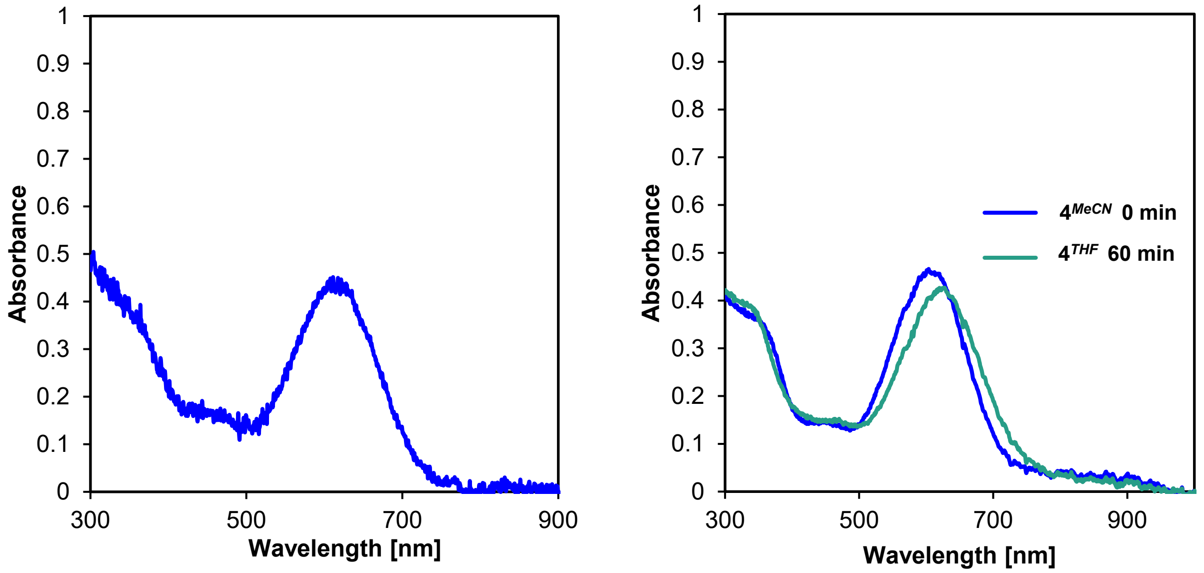

Figure S13. UV-vis spectra of 4^{MeCN} in toluene (left) and THF (right), c ≈ 10^{-5} M.

a)

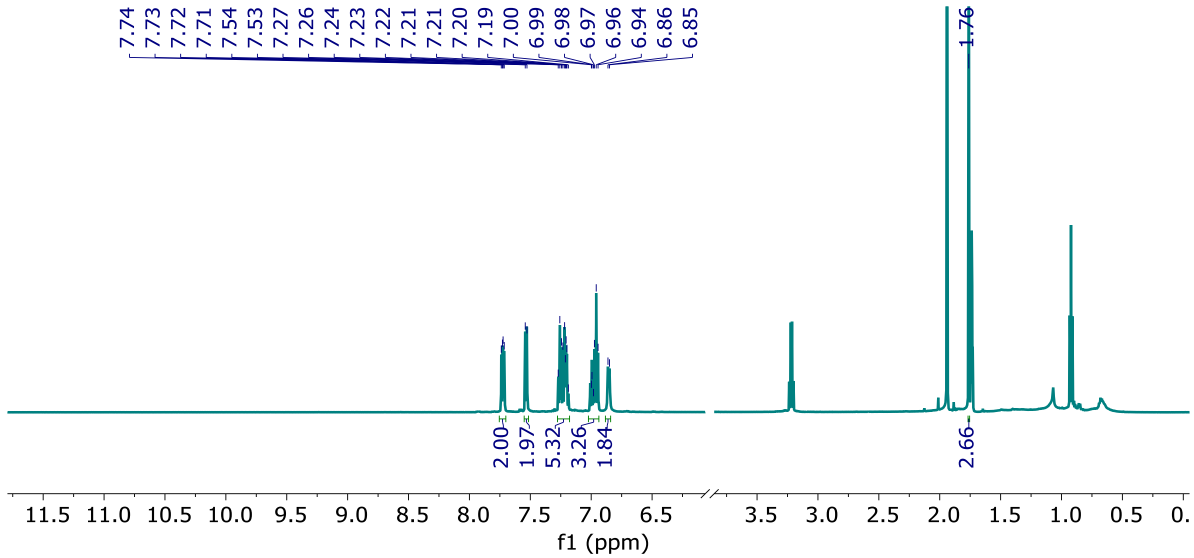

b)

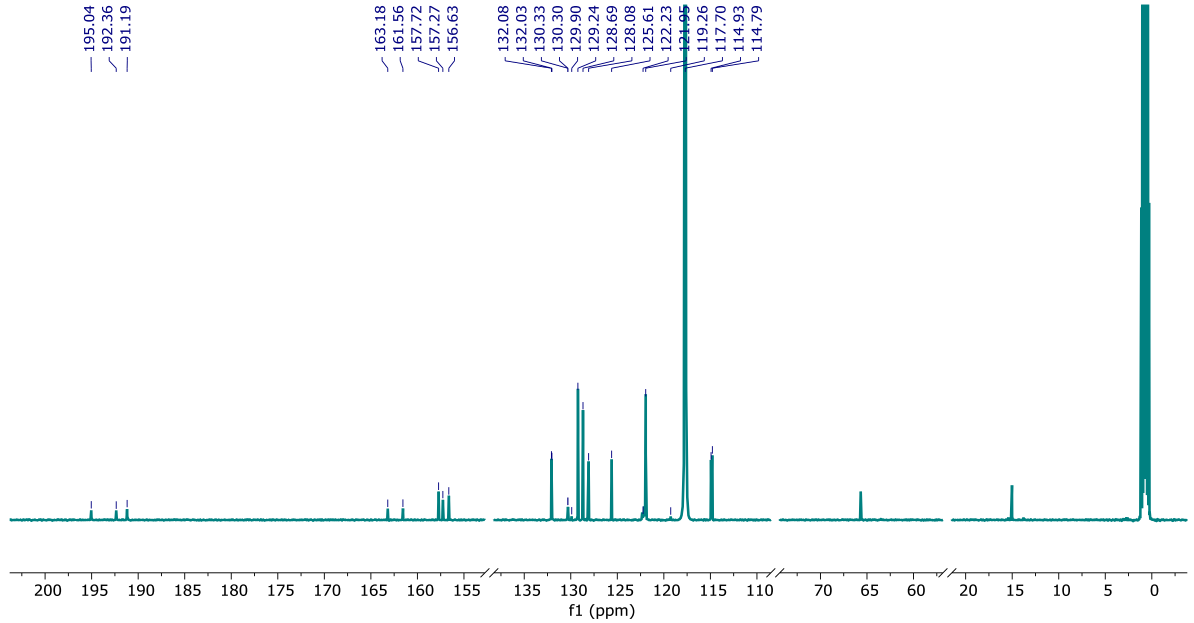

Figure S14. a)^{1}H NMR and b)^{13}C{^{1}H} spectra of 4^{MeCN} in THF-d_8 at rt.

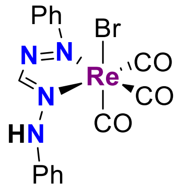

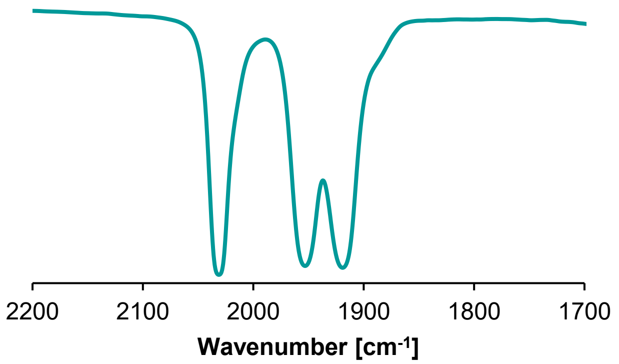

Figure S15. FT-IR spectrum of H5^{Br} in THF.

a)

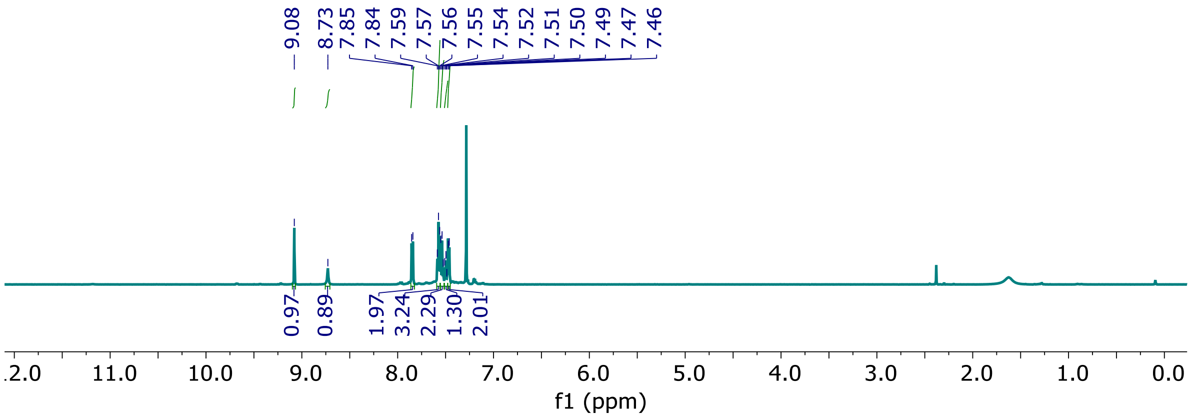

b)

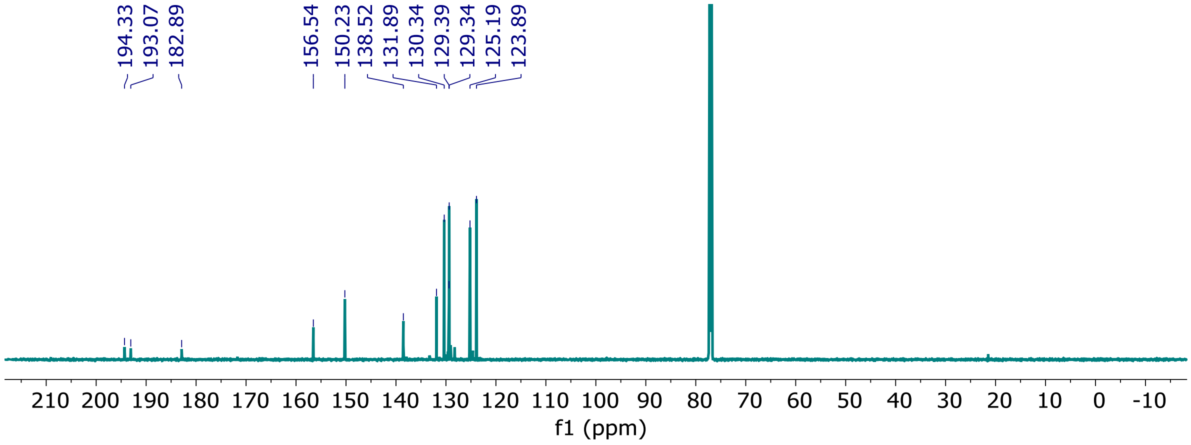

Figure S16. a)^{1}H and b) ^{13}C{^{1}H} NMR spectra of H5^{Br} in THF-d_8 at rt.

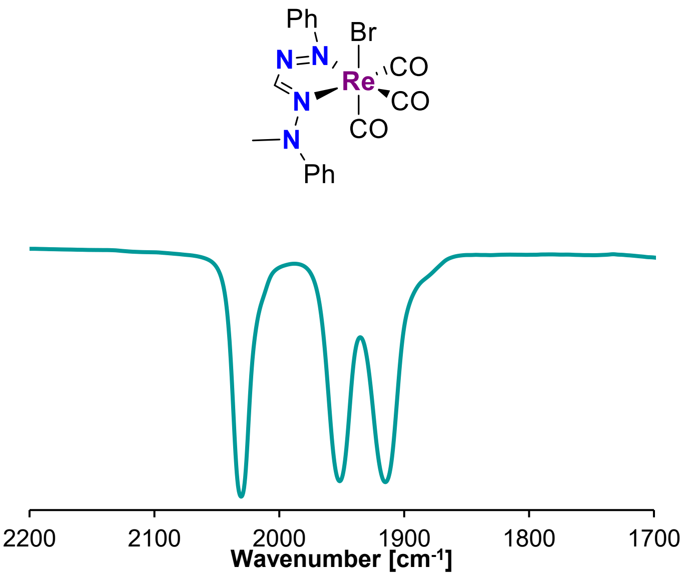

Figure S17. FT-IR spectrum of Me5^{Br} in THF.

a)

b)

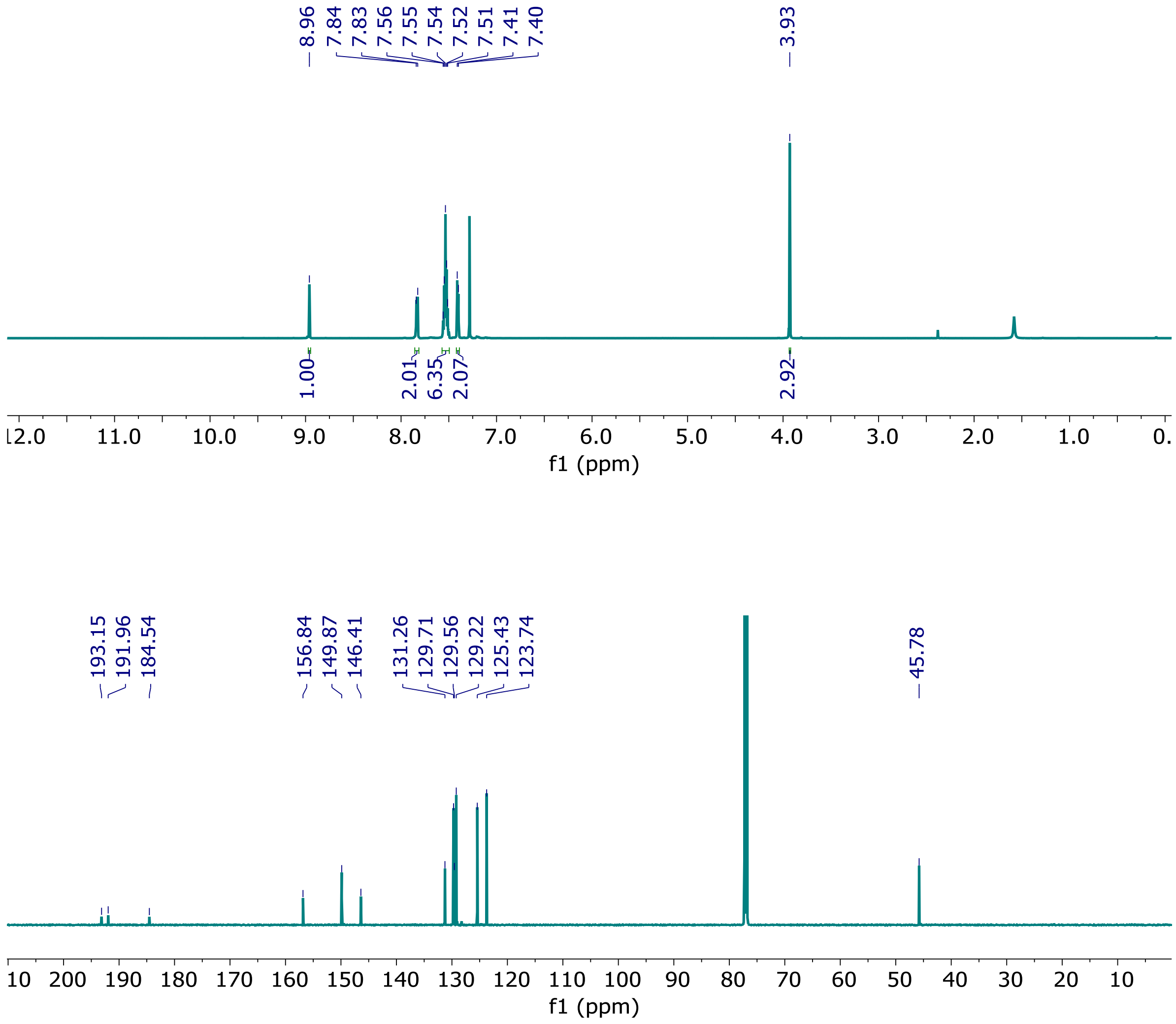

Figure S18. a)^{1}H and b) ^{13}C{^{1}H} NMR spectra of Me5^{Br} in THF-d_8 at rt.

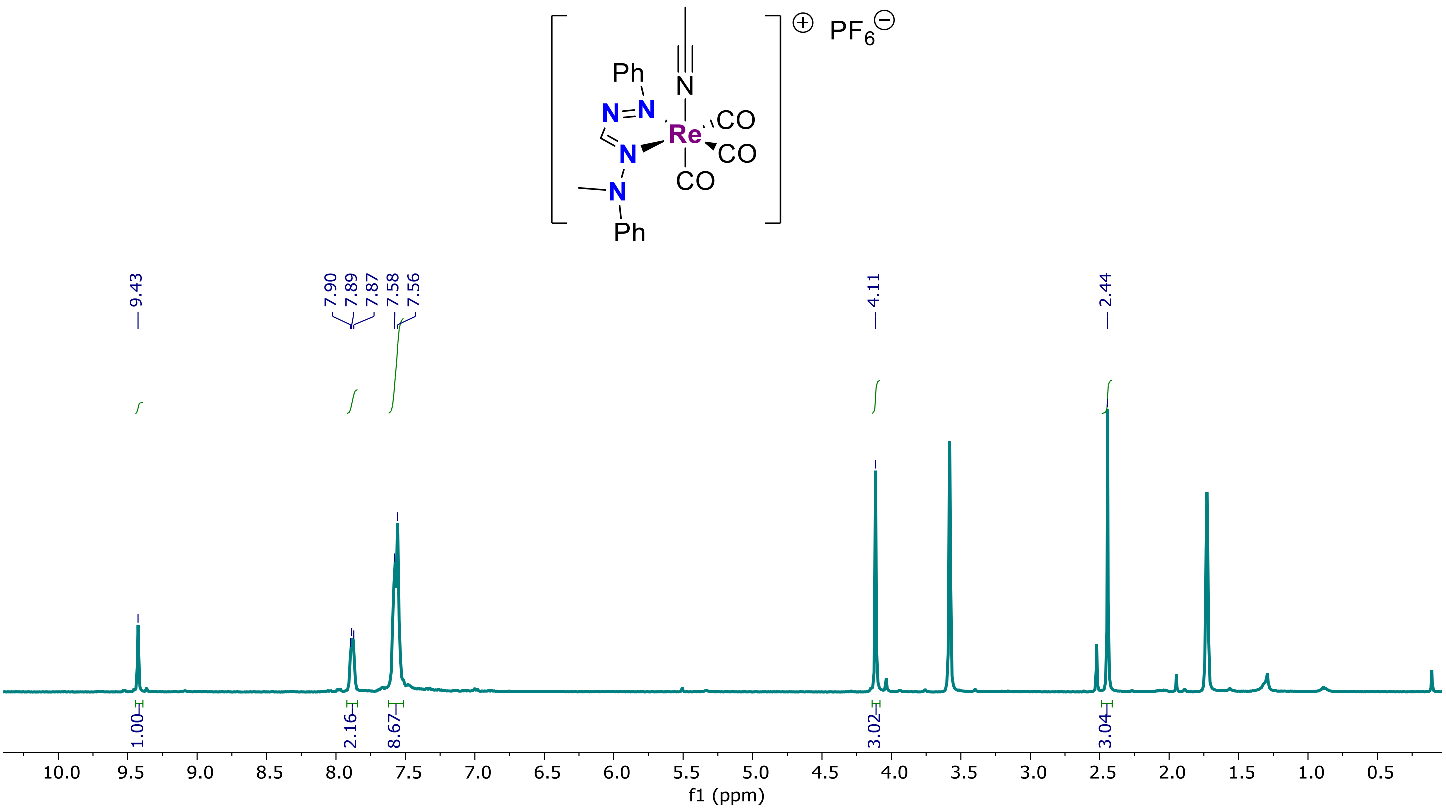

Figure S19. ^{1}H NMR spectrum of [Me5^{MeCN}][PF_6] in THF-d_8 at rt.

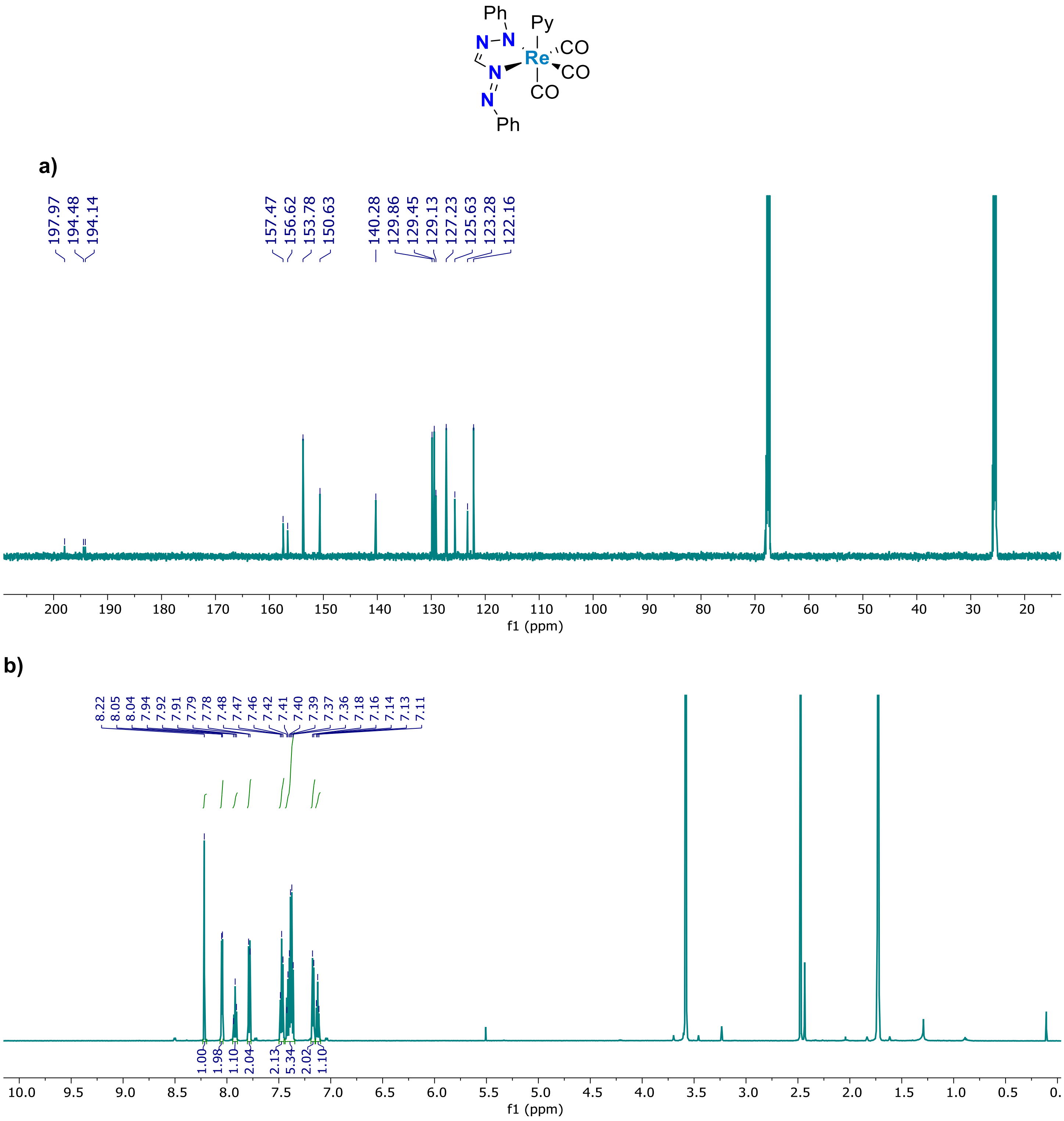

Figure S20. a) ^{1}H and b) $^{13}C\{^{1}H\}$ NMR spectra of 5^{py} in THF-d_8 at rt.

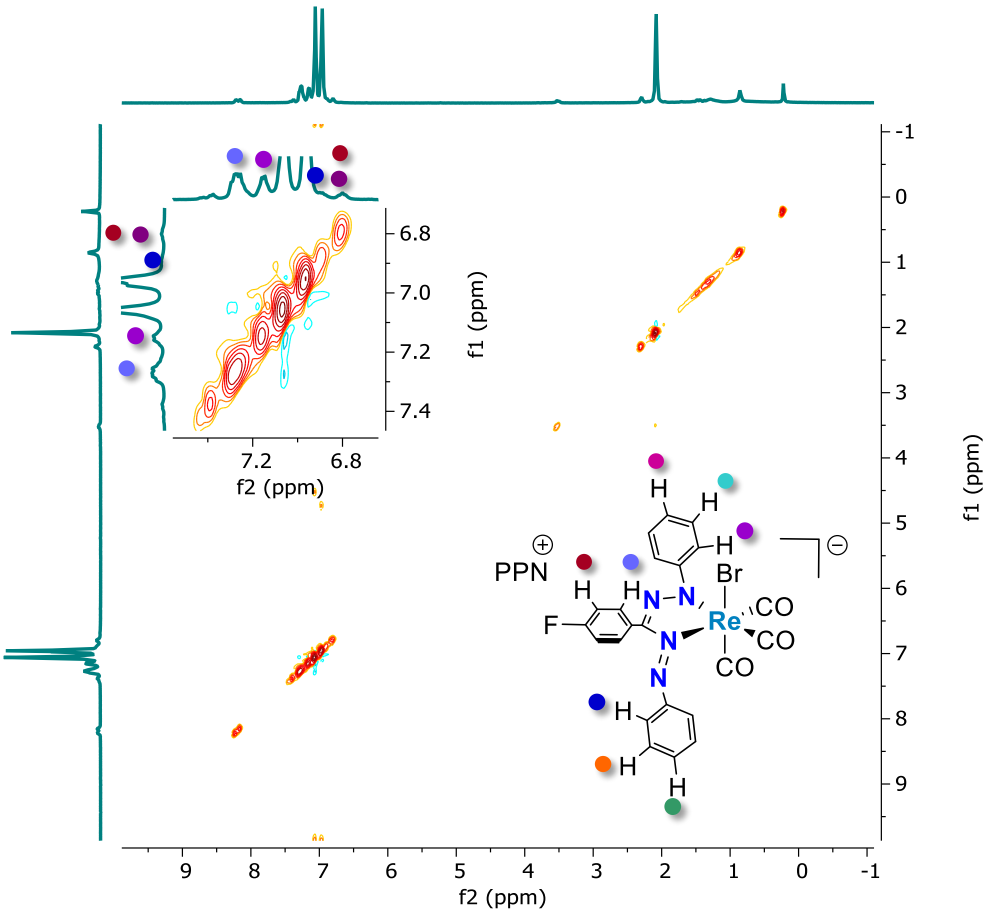

Figure S21. ^{1}H EXSY NMR spectrum of [PPN][4^{Br}] in Toluene-d_8 at 80ºC (mixing time 0.5 s). The lack of crosspeaks in the aromatic region indicates that no dynamic behavior is observed at the NMR timescale (5-membered ⇌ 6-membered).

Photophysical studies

Luminescence spectroscopy was performed with a Fluorolog®-3 spectrometer from HORIBA Jobin Yvon. A four-window cuvette from Hellma® Analytics (d = 1 cm) was used. A TBX Picosecond Photon Detection Module from HORIBA Jobin Yvon served as detector. In time-dependent luminescence spectroscopy a Nano-LED pulsed diode light source (λ_{max} = 320 nm) from HORIBA Scientific combined with a Single Photon counting controller FluoroHub from HORIBA Jobin Yvon as a pulse control unit were used. Luminescence decay data were corrected for scattered light by a separate measurement of a non-emissive silica suspension at equal excitation and detection wavelength (λ = 320 nm). Lifetimes were obtained from bi-exponential fits of the luminescence decay using fit options implemented in originPro® from OriginLab®. Excitation in steady state luminescence spectroscopy was performed using a Xenon Short Arc Lamp from Ushio Inc. light source.

a)

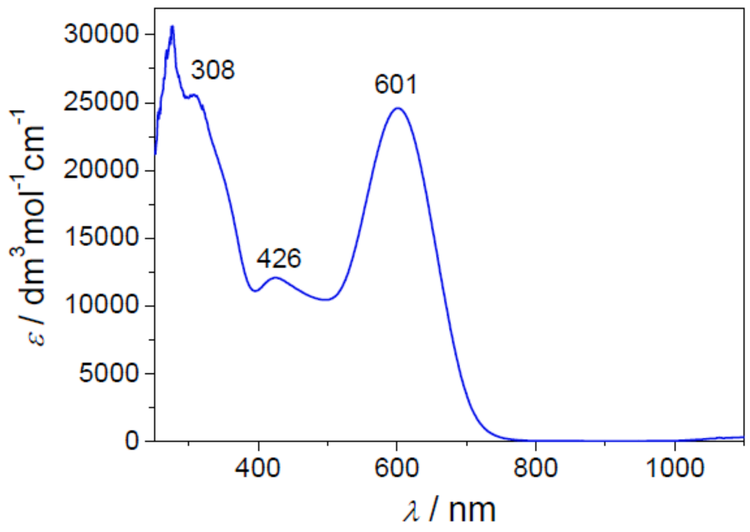

b)

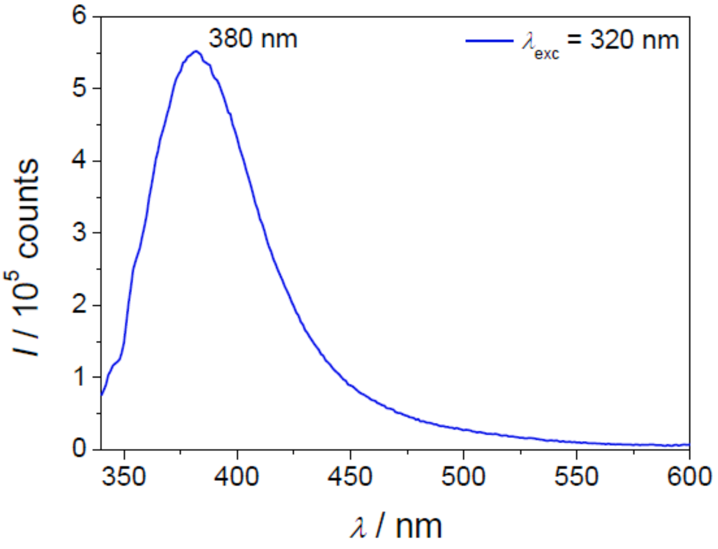

Figure S22. a) Absorption and b) emission spectra of [PPN][4^{Br}] recorded in acetonitrile.

a)

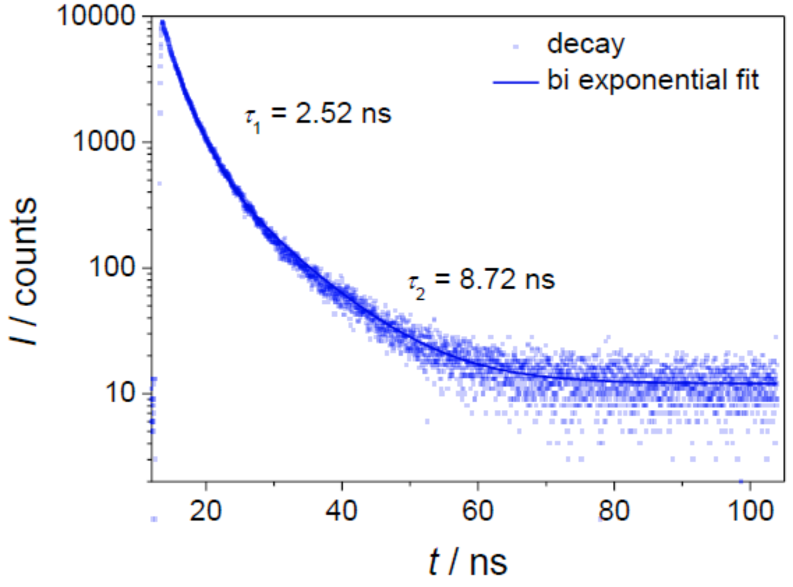

b)

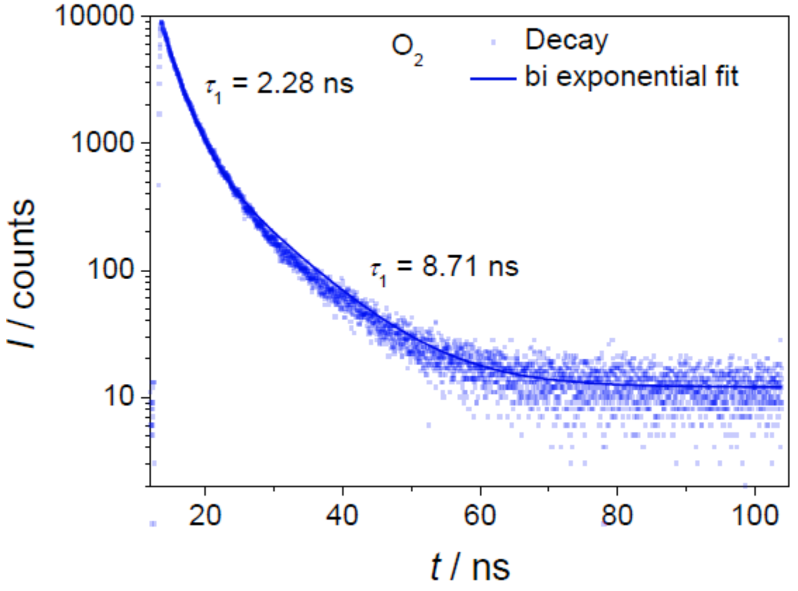

Figure S23. Lifetime decay of [PPN][4^{Br}] a) under N_2 and b) under O_2 recorded in acetonitrile.

Electrochemical studies

Cyclic voltammograms were recorded in a CHI600C potentiostat under N_2 atmosphere at room temperature in dry acetonitrile containing 0.1 M of NBu_4PF_6. The solutions were purged for 5 min with a stream of N_2 prior to the electrochemical measurements. CV was performed using a three-electrode setup comprised of a glassy carbon working electrode (3 mm^2), a silver wire as pseudoreference electrode, and a Pt wire as a counter electrode. The working electrode was polished before each measurement with an alumina slurry (0.05 μm), rinsed with distilled water, and sonicated for a few minutes, then washed with acetone. The concentration of the analyte was 1 mM in all cases. Ferrocene was added at the end of each experiment as an internal reference.

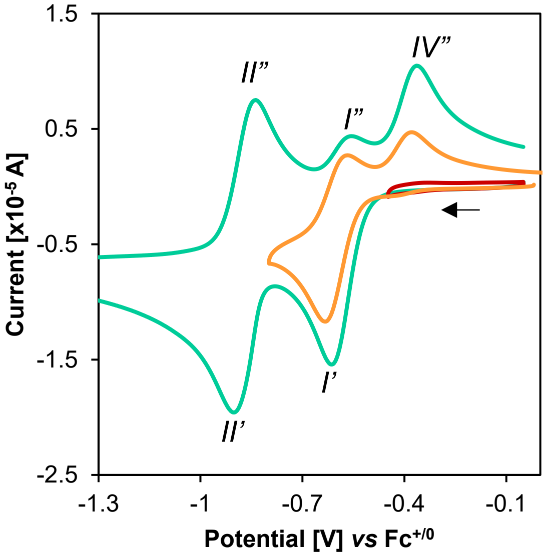

Figure S24. CVs of complex Me5^{Br} in acetonitrile at 50 mV/s at different potential windows showing the first cycle.

a) b)

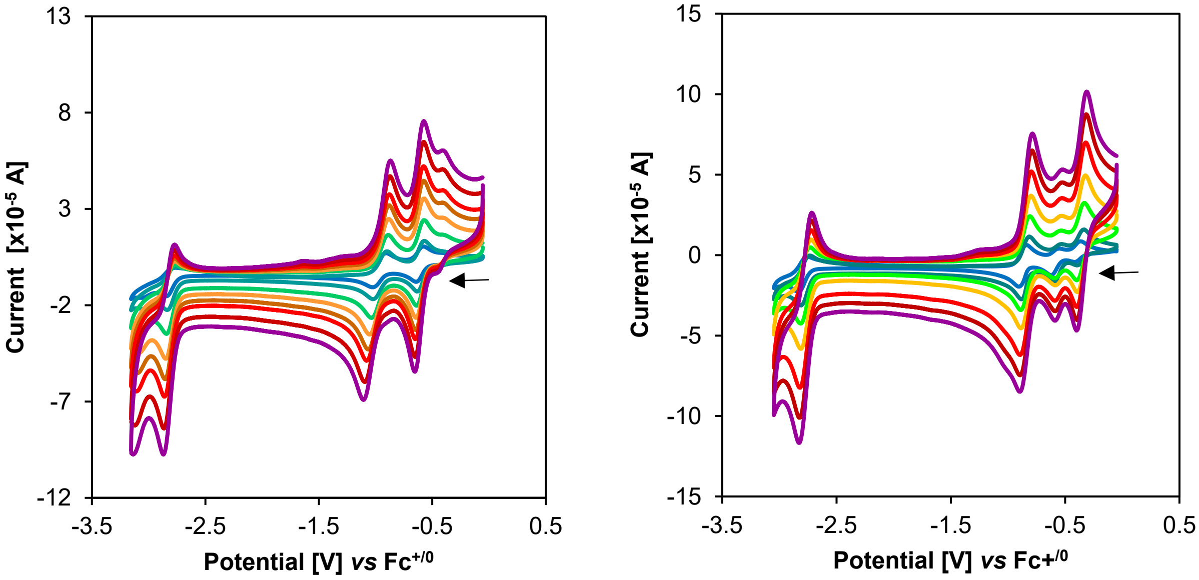

Figure S25. CVs at different scan rates (0.05, 0.1, 0.25, 0.5, 1, 1.5 and 2V/s) showing the second cycle of a) Me5^{Br} and b) Me5^{Br} + 50 eq of NBu_4Br.

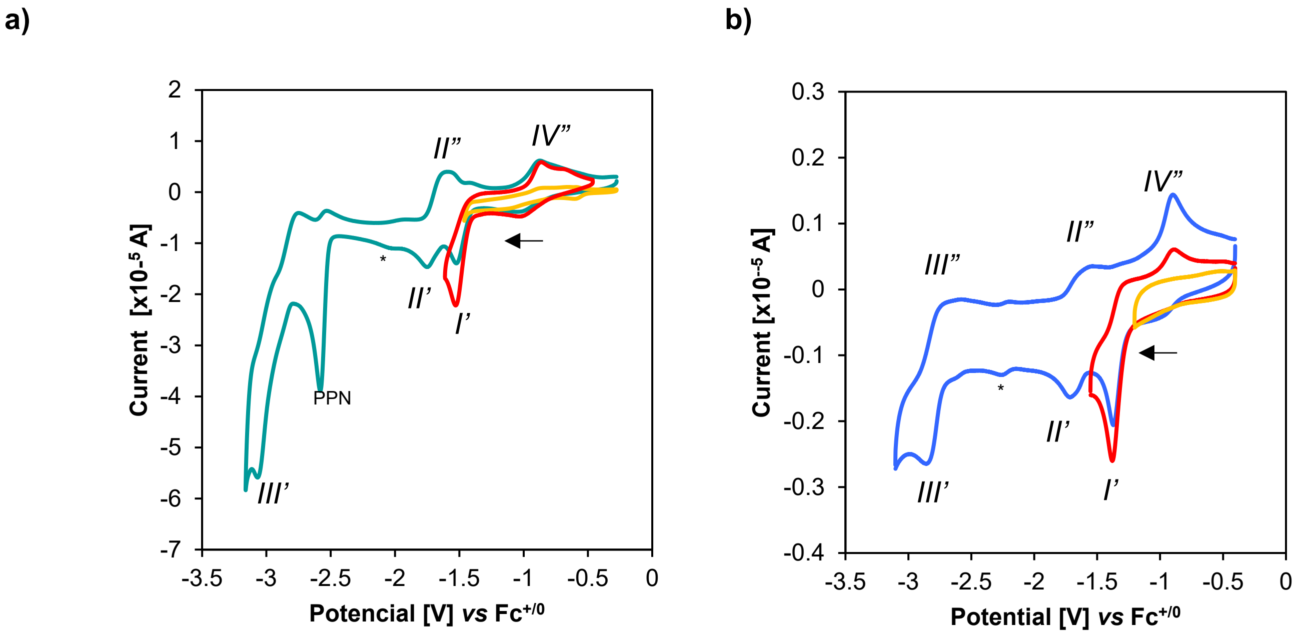

Figure S26. CVs of complexes a) [PPN][4^{Br}] and b) 4^{MeCN} in acetonitrile at 50 mV/s at different potential windows showing the second cycle.

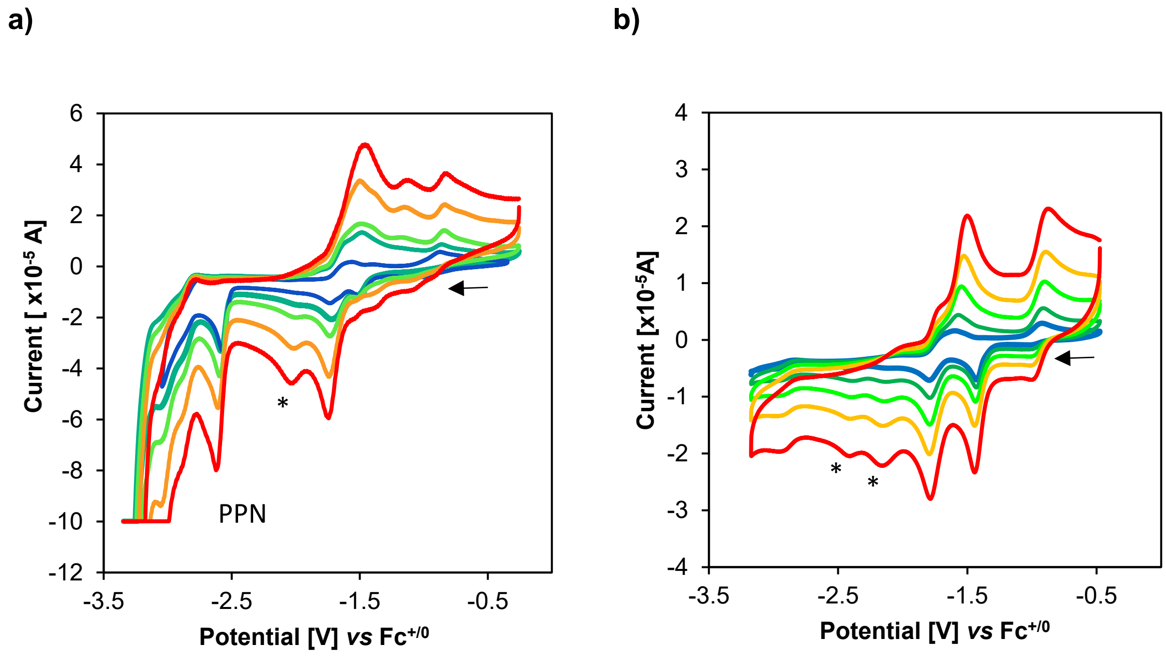

Figure S27. CVs at different scan rates (0.05, 0.1, 0.2, 0.5, and 1 V/s) showing the second cycle of a) [PPN][4^{Br}] and b) 4^{MeCN} in acetonitrile, 0.1 M [NBu_4][PF_6].

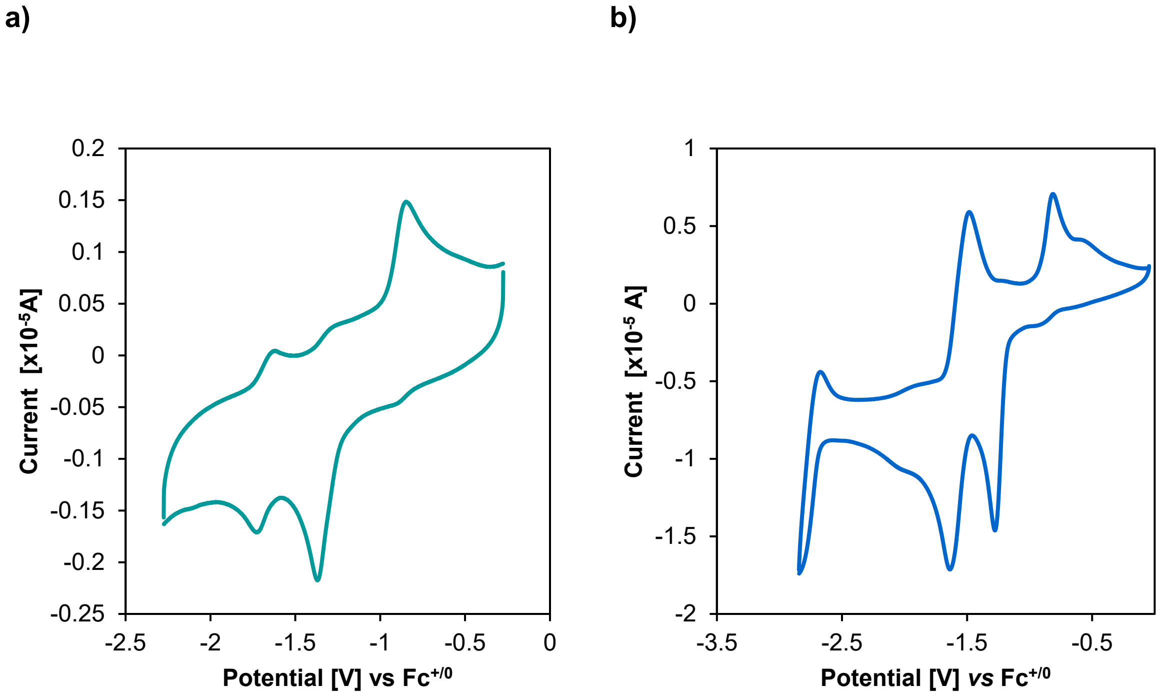

Figure S28. CV of a) 4^{MeCN} in DMF at 50 mV/s and b) 5^{py} in MeCN showing the second cycle.

Spectroelectrochemistry

FT-IR spectroelectrochemical measurements were performed on a Bruker Invenio-R spectrometer with a MCT-Detector using an OTTLE cell containing a platinum mesh as the working electrode, Ag wire as pseudoreference electrode, and a platinum counter electrode. The potential was applied by a Gamry Reference 600 potentiostat. The spectra were recorded every twelve seconds during a linear potential sweep with a scan rate of 2.5 mV/s. Redox potentials referenced against ferrocene were determined by comparing the potential values of the linear potential sweep against the potentials on the cyclic voltammetry experiment (which were internally referenced with ferrocene).

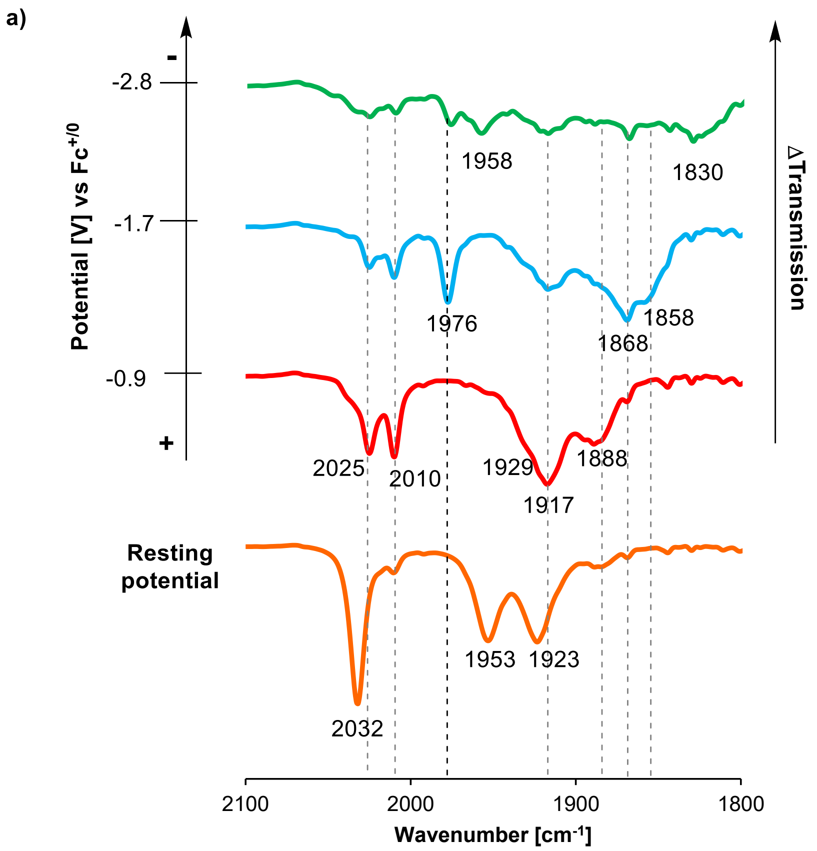
a)
Potential [V] vs Fc+/0
-2.8
-1.7
-0.9
-
+
ΔTransmission
1958
1830
1976
1868
1858
2025
2010
1929
1917
1888
Resting potential
1953
1923
2032
2100
2000
1900
1800
Wavenumber [cm-1]

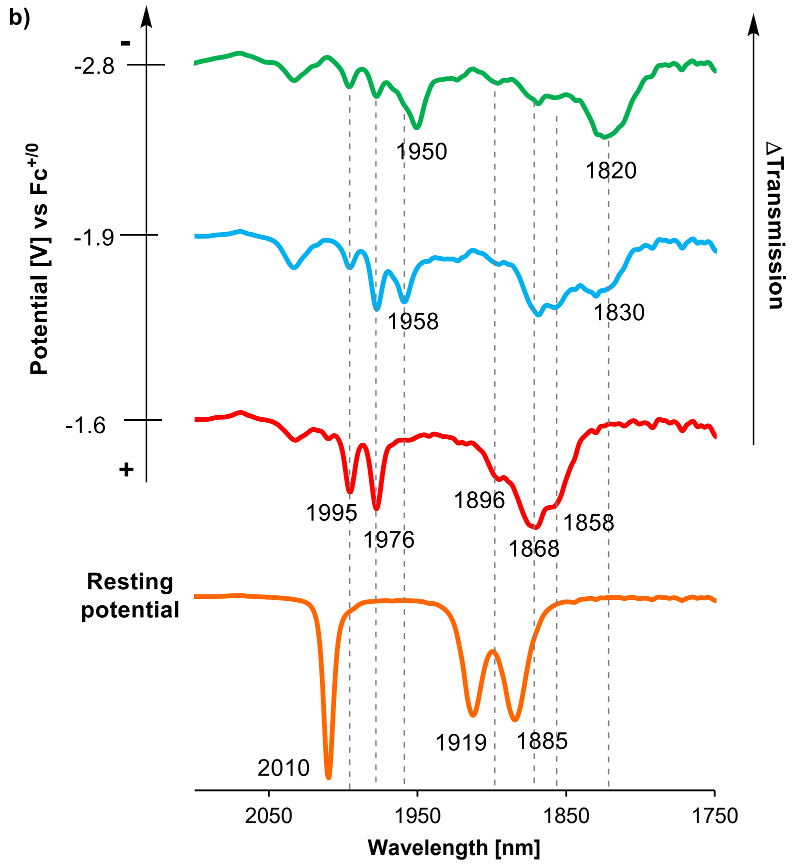
b)
Potential [V] vs Fc+/0
-2.8
-1.9
-1.6
-
+
ΔTransmission
1950
1820
1958
1830
1995
1976
1896
1868
1858
Resting potential
2010
1919
1885
2050
1950
1850
1750
Wavelength [nm]

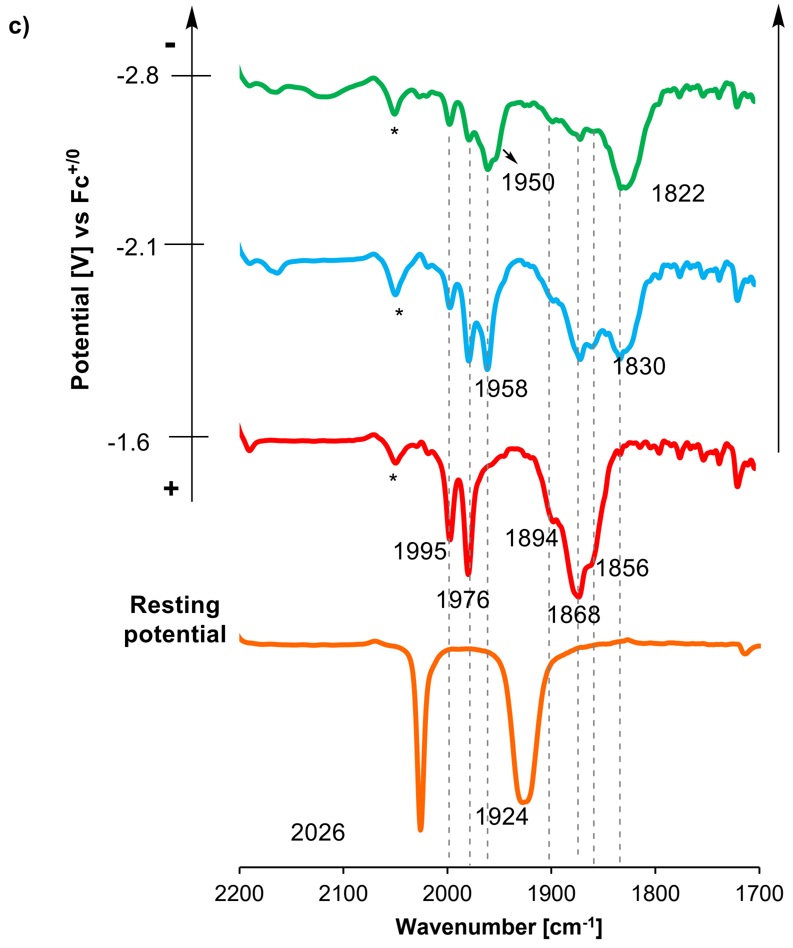

Figure S29. FT-IR-SPEC of a) $H4^{Br}$ b) [PPN][4^{Br}] and c) 4^{MeCN} under N_2 in an 0.1 M NBu_4PF_6 solution in acetonitrile.

Chemical reductions

Chemical reductions were carried out inside the glovebox using dry and degassed solvents. Stock solutions from the reducing agent $(Co(Cp^*)_2$ and the substrate were freshly prepared in 2.4 mM concentration in acetonitrile. Equivalent volumes of the stock solutions of both the reducing agent and the complex were mixed, and the FT-IR cell was filled inside the glovebox. The spectra were recorded immediately outside the glovebox.

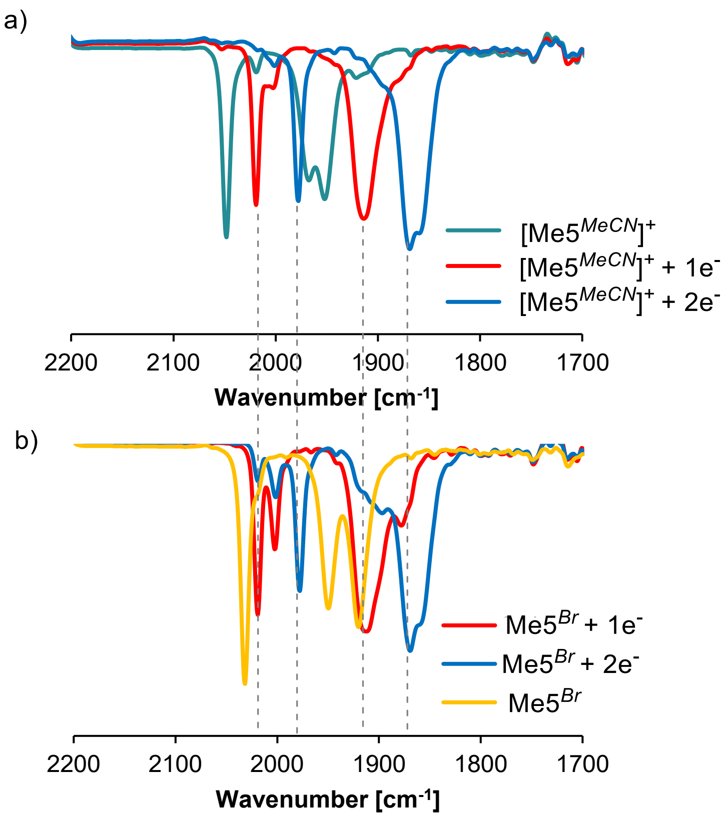

Figure S30. FT-IR upon chemical reduction with $Co(Cp^*)_2$ of a) [Me5^{MeCN}][PF_6] and b) Me5^{Br} in acetonitrile at rt.

Electrochemistry under CO_2

All measurements were recorded in 0.1 M solution of NBu_4PF_6 in dry acetonitrile at a scan rate of 50 mV/s, unless otherwise is stated. For CV, the voltammograms were recorded in a custom-made cell with a three-electrode setup using a glassy carbon (3 mm^2) working electrode, a Pt wire counter electrode, and a Ag/AgCl wire pseudoreference electrode. The experiments were measured on CHI600C potentiostat. The solution was purged with a stream of N_2 prior to starting the experiments. Upon recording the voltammogram of the complex, the electrolytic solution was saturated with CO_2 for 10 min (≈0.28 M), then the CV was measured. In experiments with an added proton source, phenol was added (0.500 g, 0.53 M) into the CO_2-saturated solution, and the voltammogram was collected. The electrode was polished between each measurement.

For controlled potential electrolysis (CPE), the experiments were carried in an air-tight two-port cell, where the Pt spiral counter electrode was separated from the bulk solution by introducing the electrode in a capillary with a porous glass frit containing supporting electrolyte solution (0.1 M of NBu_4PF_6). A glassy carbon rod (3 mm) was used as a working electrode and a Ag/$AgNO_3$ as a reference electrode. The cell was charged with 3 mL of supporting electrolyte solution with a 1mM concentration of the catalyst. The solution was purged for 10 min with CO_2, then 500 µL of methane were injected as an internal standard. After electrolysis, samples from the headspace were taken and analyzed by gas chromatography. Gas quantification was carried out on a a Shimadzu GC-2014 equipped with a TCD detector and on a ShinCarbon column. The number of moles of H_2 and CO were determined in independent experiments, where helium and argon were used as carrier gas, respectively. Calibration curves were built by injecting known quantities of the respective gas (CO or H_2) into the bulk electrolysis cell and mixed with a constant amount of CH_4. Faradaic efficiencies were determined by multiplying the number of moles of CO by 2F/Q, where 2 corresponds to the number of electrons needed for the reduction of CO_2 to CO, F stands for the Faraday constant equals to 96485 C/mol, and Q the charge passed during the bulk electrolysis experiment.

For the controlled potential electrolysis experiments at lower overpotential, another custom-made cell was used, where the cathode and the anode were separated by a porous glass frit. A reticulated vitreous carbon (Duocel 45 ppi) working electrode and a Ag/AgCl wire pseudo reference electrode were placed in the cathode chamber with 6 mL of the supporting electrolyte solution, and the catalyst added in a 1mM concentration. On the anode chamber 6 mL of supporting electrolyte were added and a Pt mesh was used as a counter electrode. Both sides were purged for 5 min with N_2 and 10 min each with a stream of CO_2. Upon electrolysis 1 mL of the headspace was injected into a HP 5890 series II instrument with a TCD detector. The sample was passed through a Varian CP-PoraBOND Q (50 m x 0.53 m x 10 µm) and an Agilent Technologies HP-Molsieve (30 m x 0.53 mm x 50 µm) column. The amount of CO was determined by comparing the area against a calibration mixture of known concentrations.

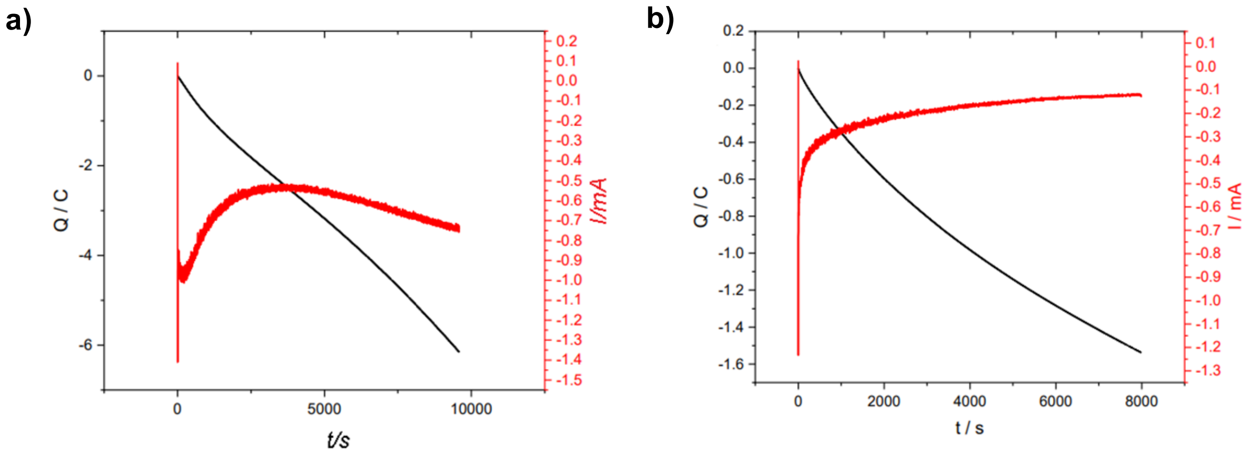

Figure S31. CPE of 4^{MeCN} -2.8 V vs $Fc^{+/0}$ under a) CO_2 and b) CO_2 + 5% phenol.

Table S1.Data from controlled potential electrolysis for formazanate complexes 4^{MeCN} and [PPN][4^{Br}] under CO_2.

Complex	CO_2				
	Low Overpotential		High Overpotential		
	FE_{CO} %	TON	FE_{CO} %	FE_{HCOOH} %	TON
4^{MeCN}	25	0.63	48	---	4.97
[PPN][4^{Br}]	19	0.45	9	<1	0.67

Table S2. Data from controlled potential electrolysis for 4^{MeCN}, [PPN] [4^{Br}] and Me5^{Br} under CO_2 and 5% phenol.

Complex	CO_2 + 5% Phenol High Overpotential		
	FE_{CO}%	TON	FE_{H2}%
4^{MeCN}	19	0.66	1
[PPN][4^{Br}]$^-$	21	1.62	2
Me5^{Br}	16	0.93	7

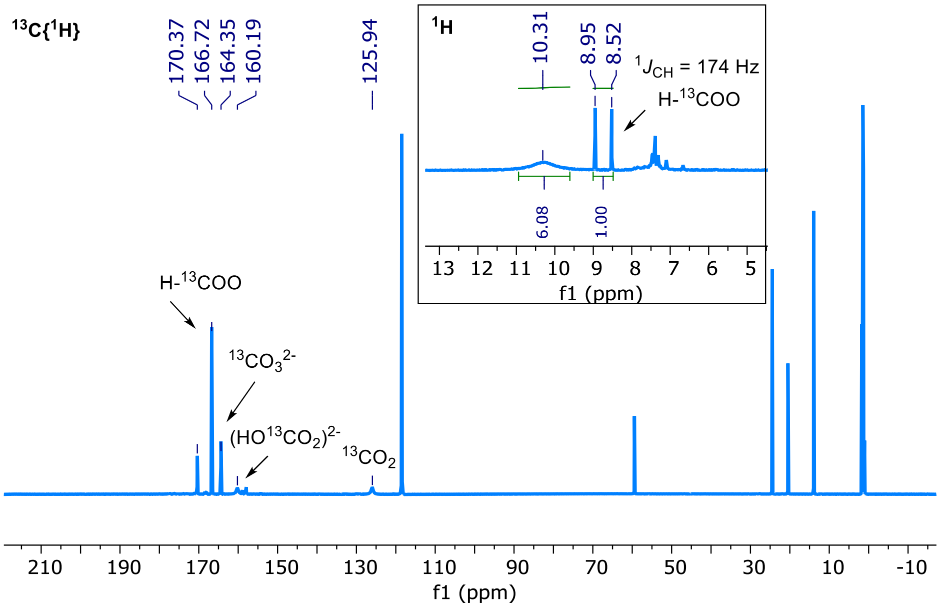

Figure S32. NMR spectra after electrolysis of [PPN][4^{Br}] under 'dry' CO_2 at -2.5V *vs* $Fc^{+/0}$

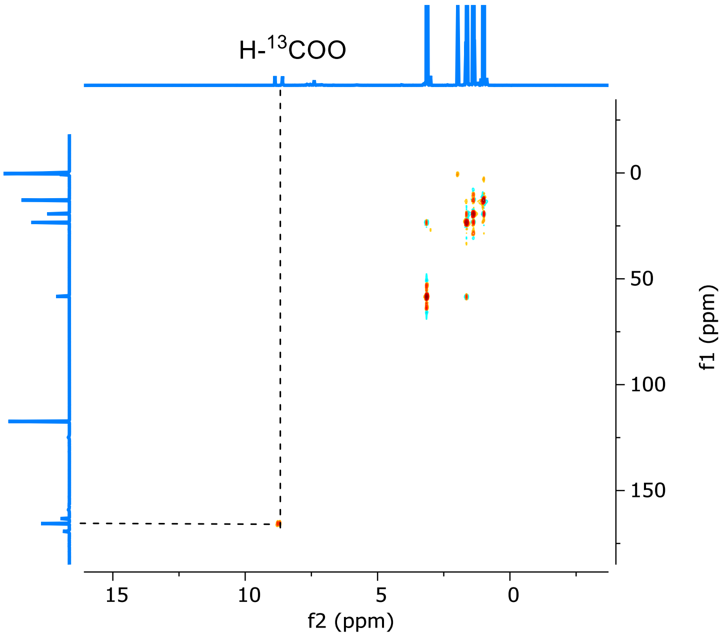

Figure S33. HSQC experiment of the cathode solution upon CPE under $^{13}CO_2$ at -2.5V *vs* $Fc^{+/0}$.

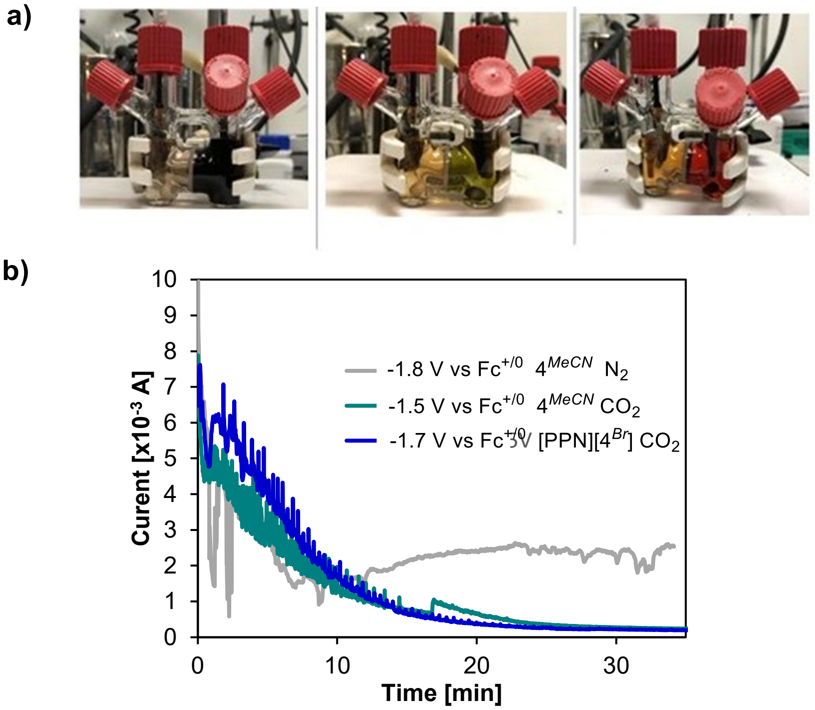

Figure S34. a) Bulk electrolysis solutions in an H-cell of 4^{MeCN}. The cathode on the right compartment: before electrolysis (left), after electrolysis under CO_2, (middle), and under N_2 (right). b) CPE at low overpotentials for 4^{MeCN} and $[PPN][4^{Br}]$ under CO_2 and N_2.

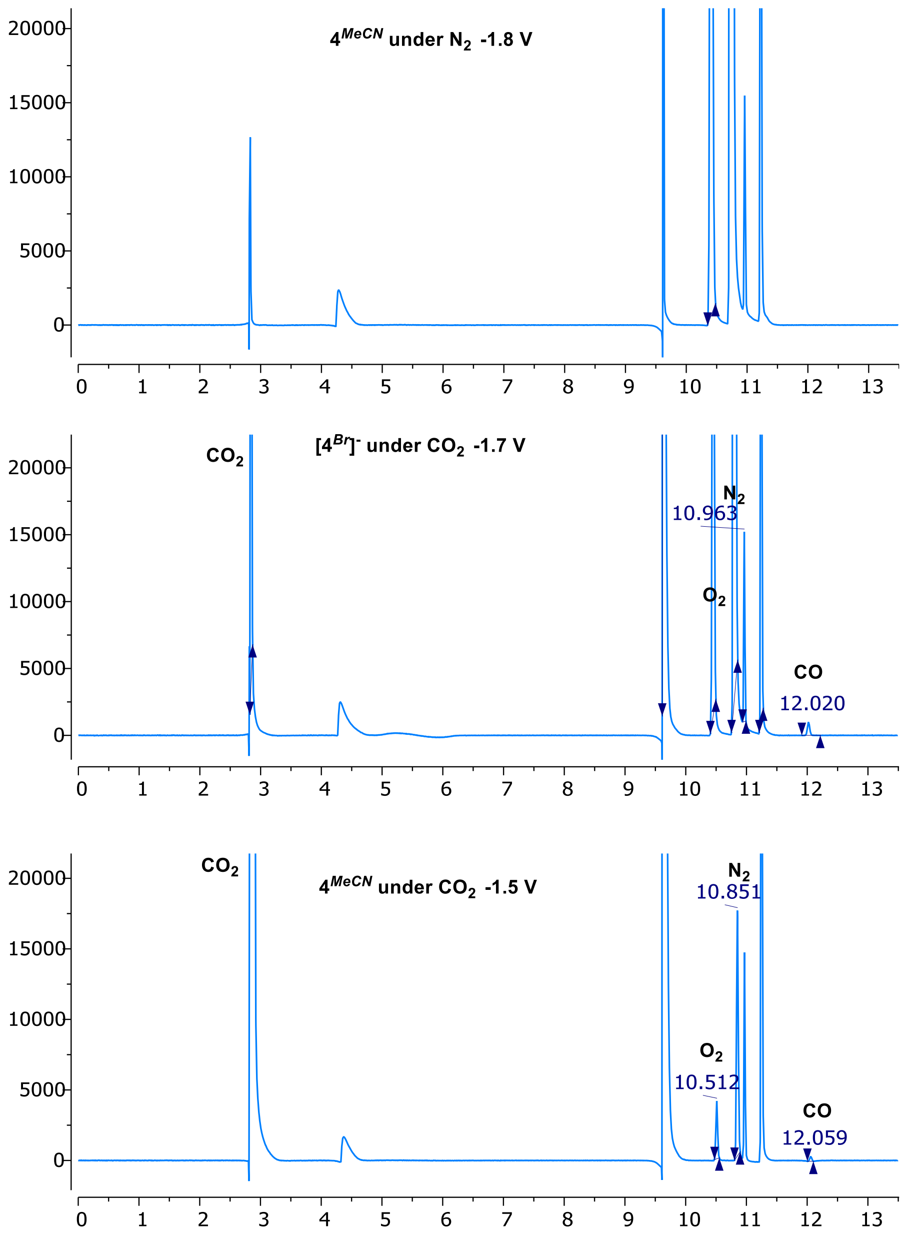

Figure S35. Chromatograms of the analysis of the headspace of electrolysis at low overpotentials. CO was only detected in samples sparged with CO_2.

Chemical reductions in the presence of $^{13}CO_2$

The samples were prepared inside the glovebox by adding 1.3 (6.8 mg, 0.021 mmol) or 2.3 (13 mg, 0.037 mmol) equivalents of the reducing agent Co(Cp*)$_2$ with one equivalent of the catalyst **4*MeCN*** (10 mg, 0.016 mmol) into an NMR Young-tube. Then, 0.5 mL of CD_3CN previously saturated with $^{13}CO_2$ were poured into the tube. The solutions were mixed at room temperature and after 2 h the NMR recorded. Samples of the one and two-electron reductions without the substrate were also prepared in the same manner for comparison.

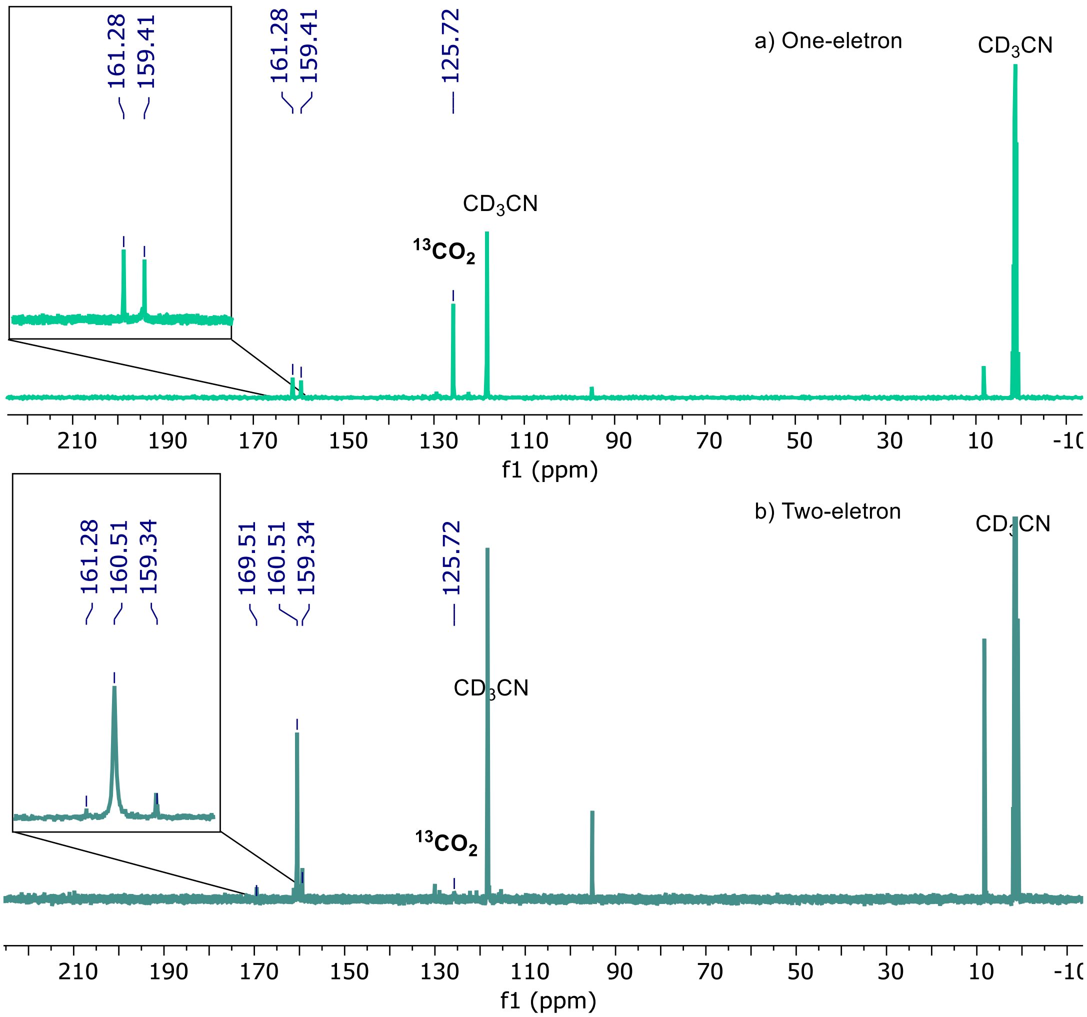

Figure S36. $^{13}C\{^1H\}$ NMR upon $^{13}CO_2$ saturation of a solution of a) mono and b) double reduced species in acetonitrile.

DFT

Density functional theory (DFT) calculations were carried out in Gaussian 16 Revision C.02[1]software, and the results were visualized using Gaussview 6[2] or Avogadro.[3] Geometry optimizations in the ground state were performed in the gas phase at a MN15L[4]/def2tzvp.[5] level of theory. Theoretical carbonyl frequencies were scaled by a factor of 0.9578 [6](see Table S2). Using the optimized geometries, TDDFT calculations were performed at CAM-B3LYP[7]/def2tzvp level of theory. The solvent effect was simulated with the continuum polarized model (CPM).[8]

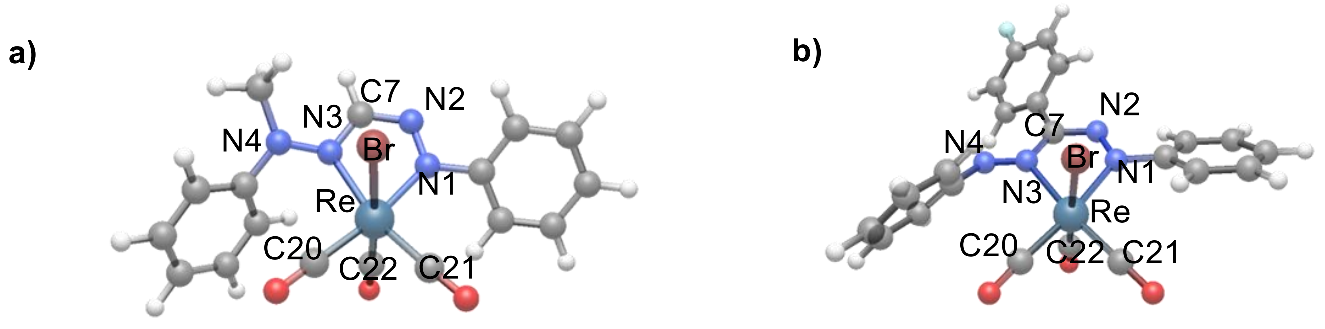

Figure S37. Optimized structures for a)$Me5^{Br}$ and b)$[4^{Br}]^{-}$.

Table S3. Experimental and theoretical bond lengths for $Me5^{Br}$ and $[4^{Br}]^{-}$.

**Bond**	**Bond length [Å]**			
	**$Me5^{Br}$**		**$[4^{Br}]^{-}$**	
	**Theoretical**	**Experimental**	**Theoretical**	**Experimental**
Re1-Br1	2.681	2.6000(14)[a]	2.641	2.6191(3)
Re1-N1	2.162	2.112(4)	2.121	2.124(2)
Re1-N3	2.250	2.192(4)	2.209	2.186(2)
Re1-C20	1.945	1.939(5)	1.965	1.941(3)
Re1-C21	1.916	1.912(5)	1.927	1.915(3)
Re1-C22	1.907	1.887(13)[a]	1.922	1.913(3)
N1-N2	1.308	1.300(6)	1.301	1.292(3)
C7-N2	1.338	1.341(7)	1.355	1.369(4)
C7-N3	1.308	1.370(6)	1.328	1.309(4)
N3-N4	1.301	1.301(7)	1.349	1.354(3)

[a]These bond lengths were restrained in the refinement, values shown are for the major disorder component; the large standard uncertainty in these numbers means that a comparison with the other compounds is not meaningful.

Table S4. Theoretical vs experimental carbonyl stretching frequencies.

Complex	Theoretical	Experimental	Complex	Theoretical	Experimental
Me5^{Br}	2022	2032	[4^{Br}]$^{—}$	1997	2010
	1960	1950		1915	1912
	1924	1919		1911	1884
[Me5^{Br}]$^{•—}$	1986		[4^{Br}]$^{2•—}$	1969	
	1908			1877	
	1880			1861	
Me5$^{•}$	2009	2001	[4]$^{•—}$	1977	1976
	1936	1876(br)		1892	1868(br)
	1920			1889	
Me5$^{MeCN•}$	2011	2019	[4^{MeCN}]$^{•—}$	1991	1992
	1932	1914(br)		1912	1894
	1929			1885	1880
[Me5]$_2$	2003		[4]$_2^{2—}$	1971	
	1991			1956	
	1935			1901	
	1924			1885	
				1877	
				1865	
[Me5]$^{—}$	1969	1977	[4]$^{2—}$	1951	1958
	1883	1869		1860	1830
	1876	1857		1827	
[Me5]$^{2•—}$	1875		[4]$^{3•—}$	1882	
	1796			1772	
	1781			1773	

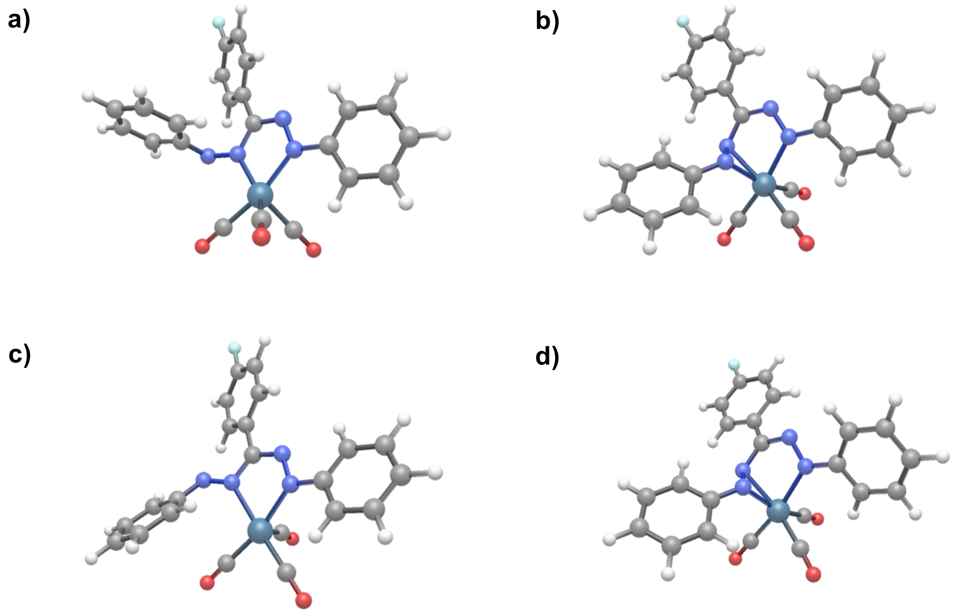

Figure S38. Optimized structures of the doubled-reduced complex $[4]^{2-}$ as five-membered ((a) S = 0 and c) S =1) and six-membered chelate (b) S= 0 and d) S= 1).

Table S5. Difference in free Gibbs energy between singlet and triplet state in $[4]^{2-}$ as a five and six-membered chelate.

	Free Gibbs Energy [kcal/mol]		
**Conformation**	**S = 0**	**S = 1**	**Difference (Triplet – singlet)**
Five-membered ($[4]_a^{2-}$)	-921529.1	-921500.3	28.8
Six-membered ($[4]_b^{2-}$)	-921528.6	-921493.0	35.6
**Difference (b-a) [kcal/mol]**	0.50		

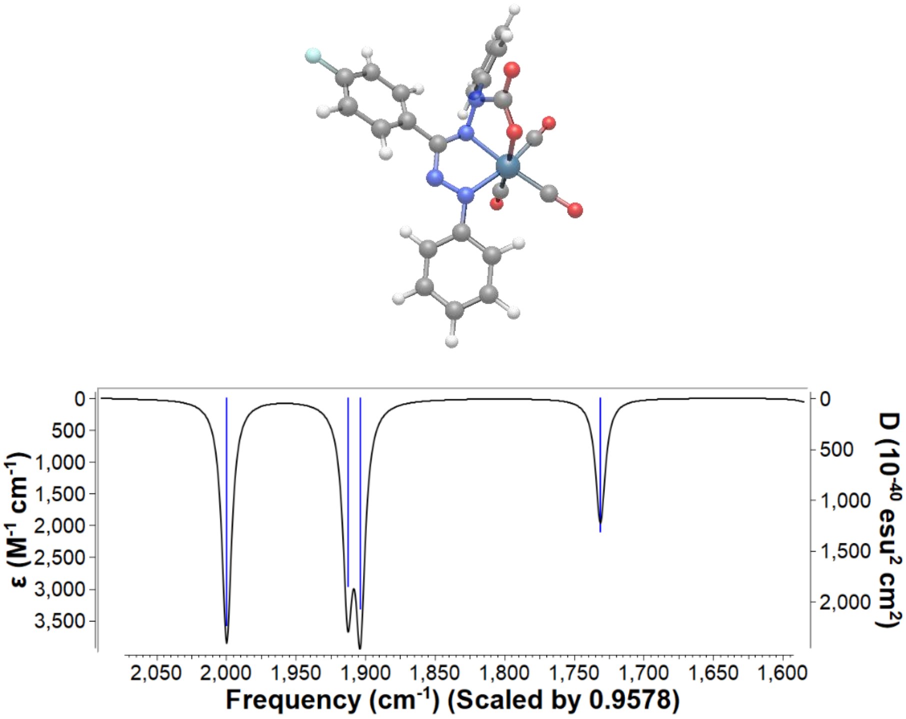

Figure S39. Computed IR spectrum for *fac*-[4$^{N\text{-}CO2}$]•– in the gas phase at MN15L/def2tzvp level of theory. Scaled analytical frequencies for ν(CO) = 1987, 1904, 1896, 1731 cm^{-1}.

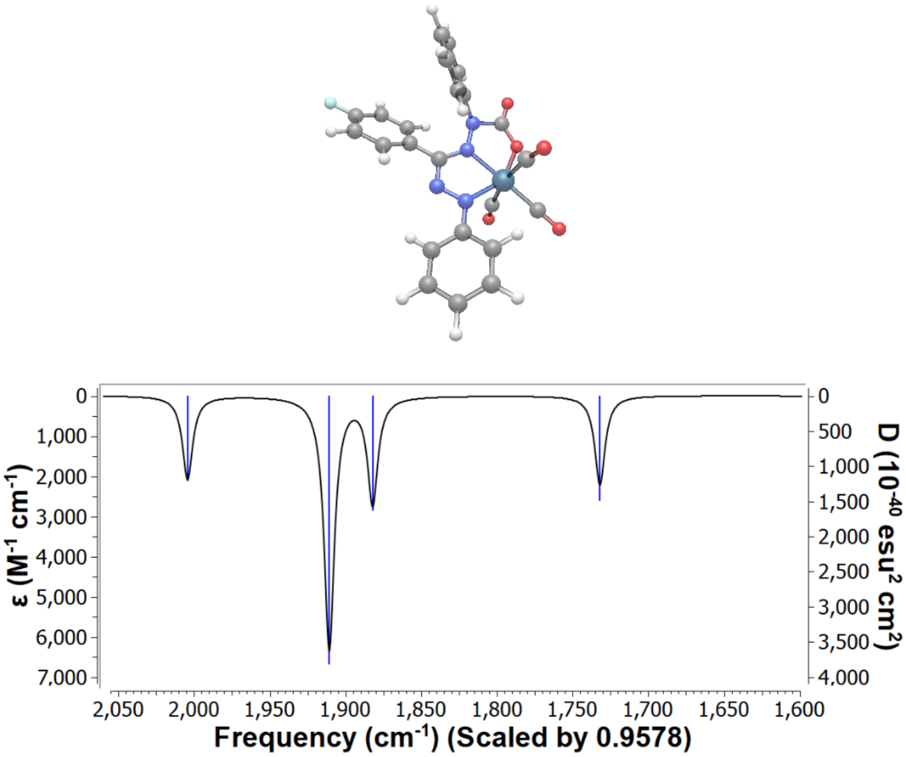

Figure S40. Computed IR spectrum for *mer*-[4$^{N\text{-}CO2}$]•– in the gas phase at MN15L/def2tzvp level of theory. Scaled analytical frequencies for ν(CO) =2000, 1907, 18, 1731 cm^{-1}.

TDDFT

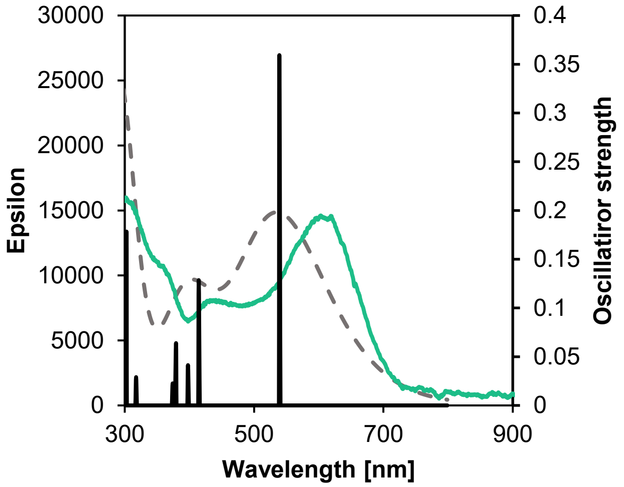

Figure S41. Comparison between experimental (turquoise trace) and theoretical (dash line) spectra for $[4^{Br}]^-$ in THF.

Table S6. Natural transition orbitals (isovalue = 0.05) for the main electronic transitions in $[4^{Br}]^-$.

Excited state	Electronic transitions	Energy (eV)/ λ_{theo}(nm)	λ_{exp}(nm)	Oscillator strength	Hole → Electron	Occupation number
1	H → L<br>H-1 → L	2.3000/539.05	607	f=0.3593	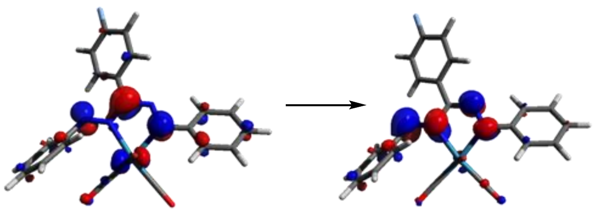	0.99247
2	H-4 → L<br>H-3 → L<br>H-1 → L<br>H → L	2.9920/414.39		f=0.1285	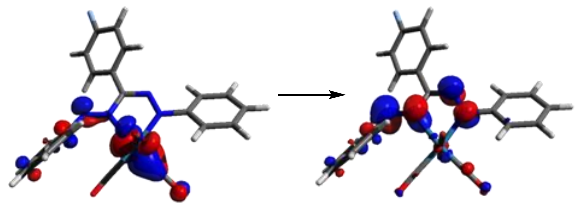	0.98679
3	H-4 → L<br>H-3 → L<br>H-2 → L	3.1171/397.76	440	f=0.0413	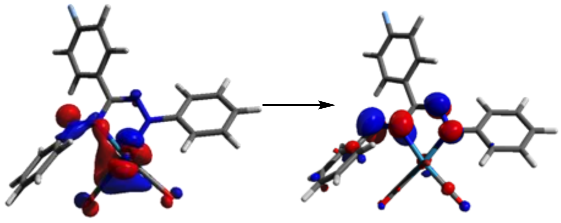	0.98464
4	H-13 → L<br>H-11 → L<br>H-4 → L<br>H-3 → L<br>H-2 → L<br>H-1 → L	3.2689/379.29		f=0.0639		0.97489

	H → L				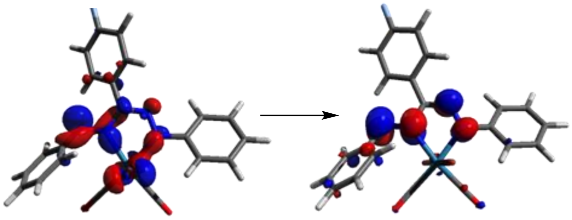	
**5**	H-3 → L<br>H-2 → L<br>H-1 → L	3.3146/374.<br>06		f=0.0226	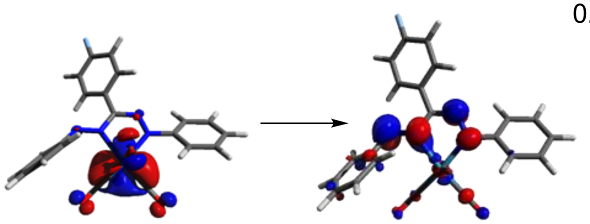	0.98891
**7**	H-2 →<br>L+2<br>H-2 →<br>L+3<br>H → L+1<br>H → L+2<br>H → L+3<br>H→ L + 6	4.0964/302.<br>67		f=0.1783	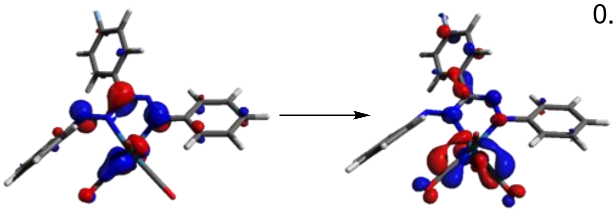	0.85630
**9**	H-2 →<br>L+1<br>H-2 →<br>L+3<br>H → L+1<br>H → L+2<br>H → L + 3<br>H → L+4	4.2329/292.<br>91	350	f=0.2962	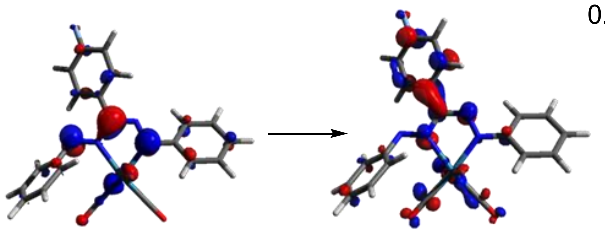	0.80441

References

(1) M. J. Frisch, G. W. Trucks, H. B. Schlegel, G. E. S.; M. A. Robb, J. R. Cheeseman, G. Scalmani, V. B.; G. A. Petersson, H. Nakatsuji, X. Li, M. Caricato, A. V. M.; J. Bloino, B. G. Janesko, R. Gomperts, B. Mennucci, H. P. H.; J. V. Ortiz, A. F. Izmaylov, J. L. Sonnenberg, D. W.-Y.; F. Ding, F. Lipparini, F. Egidi, J. Goings, B. Peng, A. P.; T. Henderson, D. Ranasinghe, V. G. Zakrzewski, J. Gao, N. R.; G. Zheng, W. Liang, M. Hada, M. Ehara, K. Toyota, R. F.; J. Hasegawa, M. Ishida, T. Nakajima, Y. Honda, O. Kitao, H. N.; T. Vreven, K. Throssell, J. A. Montgomery, Jr., J. E. P.; F. Ogliaro, M. J. Bearpark, J. J. Heyd, E. N. Brothers, K. N. K.; V. N. Staroverov, T. A. Keith, R. Kobayashi, J. N.; K. Raghavachari, A. P. Rendell, J. C. Burant, S. S. I.; J. Tomasi, M. Cossi, J. M. Millam, M. Klene, C. Adamo, R. C.; J. W. Ochterski, R. L. Martin, K. Morokuma, O. F.; J. B. Foresman, and D. J. F. Gaussian 16, Revision C.02. Wallingford CT 2019.

(2) Dennington, Roy; Keith, Todd A.; Millam, J. M. GaussView, Version 6. Semichem Inc., Shawnee Mission: KS 2016.

(3) Hanwell, M. D.; Curtis, D. E.; Lonie, D. C.; Vandermeersch, T.; Zurek, E.; Hutchinson, G. R. Avogadro: An Advanced Semantic Chemical Editor, Visualization, and Analysis Platform. *J. Cheminformatics* **2012**, *4*, 1–17. https://doi.org/10.1186/1758-2946-4-17.

(4) Yu, H. S.; He, X.; Truhlar, D. G. MN15-L: A New Local Exchange-Correlation Functional for Kohn-Sham Density Functional Theory with Broad Accuracy for Atoms, Molecules, and Solids. *J. Chem. Theory Comput.* **2016**, *12* (3), 1280–1293. https://doi.org/10.1021/acs.jctc.5b01082.

(5) Weigend, F.; Ahlrichs, R. Balanced Basis Sets of Split Valence, Triple Zeta Valence and Quadruple Zeta Valence Quality for H to Rn: Design and Assessment of Accuracy. *Phys. Chem. Chem. Phys.* **2005**, *7* (18), 3297–3305. https://doi.org/10.1039/b508541a.

(6) Sae-Heng, P.; Tantirungrotechai, J.; Tantirungrotechai, Y. Scale Factors for Carbonyl Vibrational Frequencies: A Study of Partial Hessian Approximation. *Chiang Mai J. Sci.* **2018**, *45* (7), 2797–2808.

(7) Yanai, T.; Tew, D. P.; Handy, N. C. A New Hybrid Exchange-Correlation Functional Using the Coulomb-Attenuating Method (CAM-B3LYP). *Chem. Phys. Lett.* **2004**, *393*, 51–57. https://doi.org/10.1016/j.cplett.2004.06.011.

(8) Miertuš, S.; Scrocco, E.; Tomasi, J. Electrostatic Interaction of a Solute with a Continuum. A Direct Utilization of AB Initio Molecular Potentials for the Prevision of Solvent Effects. *Chem. Phys.* **1981**, *55*, 117–129. https://doi.org/10.1016/0301-0104(81)85090-2.
